# Dichloromethyl(diaryl) Sulfonium Salts as *gem*-Dichlorocyclopropanation Reagents

**DOI:** 10.1021/acs.orglett.4c04717

**Published:** 2025-02-03

**Authors:** Bethany J. Moore, Darren Willcox

**Affiliations:** Department of Chemistry, University of Manchester, Oxford Road, Manchester M13 9PL, United Kingdom

## Abstract

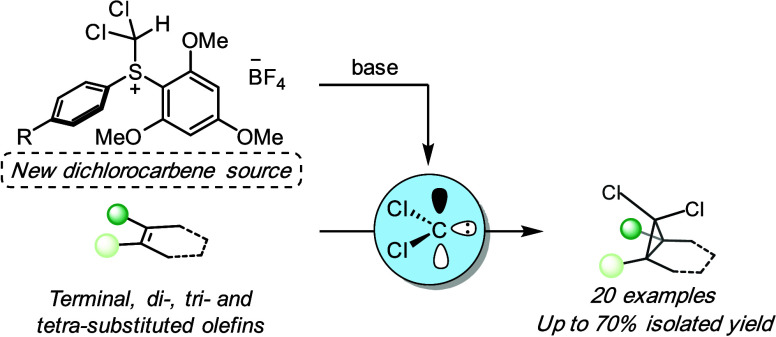

*gem*-Dichlorocyclopropanes are important structural moieties in a range of industrial sectors and have been the focus of considerable interest within the synthetic community over the years. Synthetic methodologies to access *gem*-dichlorocyclopropanes, however, remain limited. To address this challenge, we report the development of new dichloromethyl(diaryl) sulfonium salts as reagents for the *gem*-dichlorocyclopropanation of electron-rich olefins via a free dichlorocarbene pathway under mild conditions in yields of up to 70%. This methodology provides access to previously unreported *gem*-dichlorocyclopropanes.

The *gem*-dichlorocyclopropane fragment is an important motif in agrochemical compounds, such as insecticides (Cycloprothrin)^1^ and fungicides (Carpropamid and WL 28325) (Figure 1a).^2,3^ Moreover, this structural motif has garnered great interest within the scientific community due to their variable reactivity profiles, enabling them to serve as intermediates in ring expansion chemistry, Doering–Moore–Skattebøl-type reactions to access allenes,^4,5^ and hydrodehalogenation to monochlorocyclopropanes using a Grignard reagent and titanium isopropoxide.^6^ Current methodologies to access *gem*-dichlorocyclopropanes involve the generation of dichlorocarbene under relatively harsh conditions, such as α-elimination of hydrogen chloride from chloroform with strong bases under phase transfer catalysis (PTC),^7^ thermal decarboxylation of trichloroacetate salts,^8^ reaction of ethyl trichloroacetate with sodium methoxide,^9^ fluoride-activated trihalomethyl silicon reagents [(Me)_3_SiCCl_2_Br],^10^ use of highly energetic dichlorodiazirines,^11^ or stoichiometric amounts of toxic Seyferth’s reagent.^12^ Other reported methods include lithiation of diethyl trichloromethyl phosphonate,^13^ the reaction of chloroform and Grignard reagents,^14^ and the reaction of carbon tetrachloride with reduced titanium.^15^

**Figure 1:**
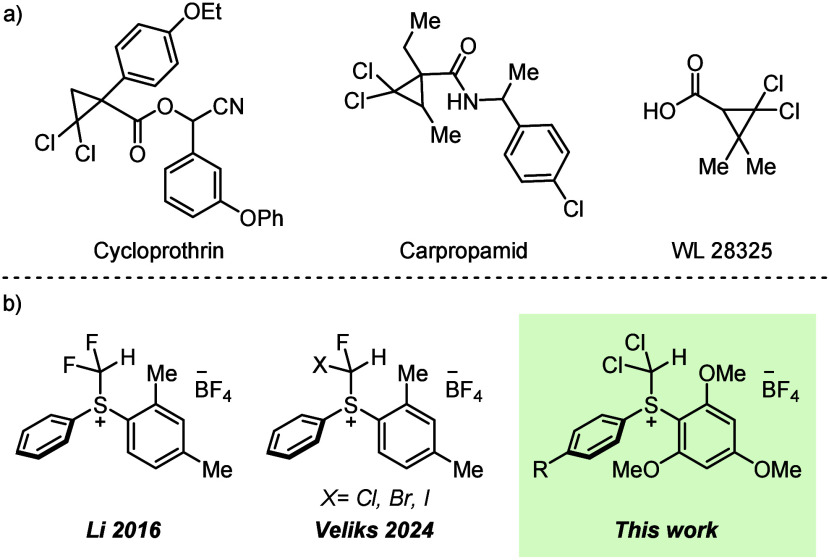
(a) Agrochemical reagents containing the *gem*-dichlorocyclopropane fragment and (b) dihalomethyl(diaryl) sulfonium salts reported for *gem*-dihalocyclopropanation.

One class of reagents that has widespread use in organic synthesis, in particular, cyclopropanation chemistry, is sulfonium salts.^16,17^ Not until recently have dihalomethyl-substituted sulfonium salts emerged as potential reagents for the *gem*-dihalocyclopropanantion reaction. These salts are considered bench-stable but have a rich reactivity profile under certain conditions, i.e., deprotonation. The first example was reported by the group of Li in 2016, where they observed *gem*-difluorocyclopropane formation upon reaction with 2,3-dimethyl-2-butene under basic conditions, suggesting the involvement of difluorocarbene.^18^ More recently, the Veliks group reported the use of unsymmetrical dihalomethyl(diaryl) sulfonium salts as free carbene precursors to yield the corresponding *gem*-dihalocyclopropanes (Figure 1b).^19^

Encouraged by these reports, we envisioned that dichloromethyl(diaryl) sulfonium salts may serve as suitable precursors for accessing dichlorocarbene. However, dichloromethyl(diaryl) sulfonium salts are unprecedented. Herein, we report the synthesis of the first dichloromethyl(diaryl) sulfonium salts and show that these compounds can be utilized in olefin *gem*-dichlorocyclopropanation under mild conditions. To begin our studies, we needed to develop a synthetic route to access dichloromethyl(diaryl) sulfonium salts. Readily available methyl(aryl) sulfoxide (**1a**–**1d**) was dichlorinated with *N*-chlorosuccinimide (2.0 equiv) to generate compounds **2a**–**2d** as pale yellow oils at room temperature in good yields (50–85%). Compounds **2a**–**2d** underwent an interrupted Pummerer reaction with 1,3,5-trimethoxybenzene followed by an anion exchange with NaBF_4_ to furnish the air- and moisture-stable dichloromethyl(diaryl) sulfonium salts **3a**–**3d** in good to excellent yields (30–90%) (Scheme 1a).

**Scheme 1:**
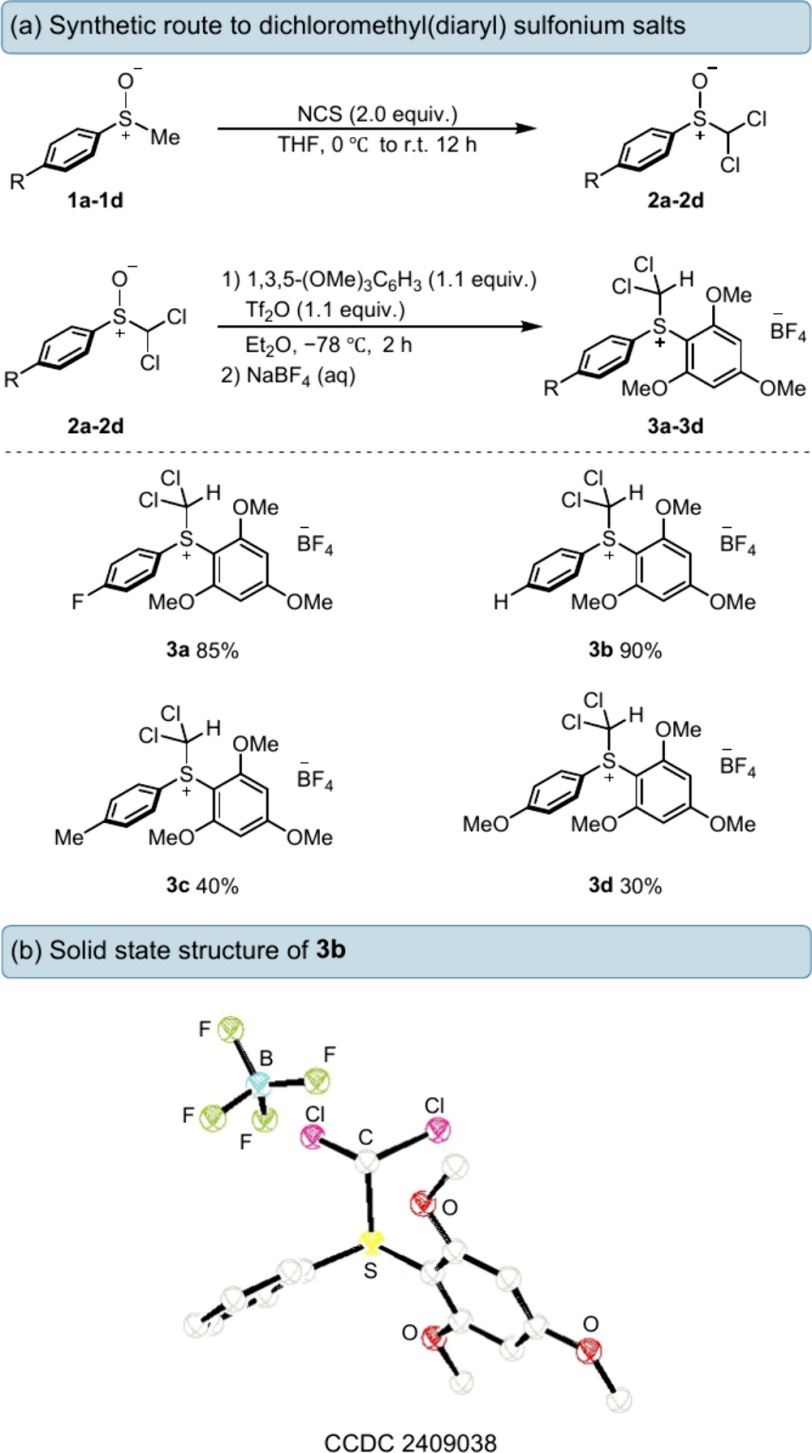
(a) Synthesis of Dichloromethyl(diaryl) Sulfonium Salts from Their Sulfoxide and (b) Solid-State Structure of Compound **3b** with Selected Atomic Labeling and Ellipsoids Drawn at the 50% Probability Level

The use of 1,3,5-trimethoxybenzene as the nucleophilic arene in the interrupted Pummerer reaction was important for isolating the corresponding sulfonium salts as easy to handle solids. When *m*-xylene was used as the nucleophilic arene, a sticky, low-melting solid was obtained. Single crystals of compound **3b**, suitable for X-ray diffraction (XRD), were obtained via an anti-solvent recrystallization of 3:2 *n*-hexane and dichloromethane, confirming the formation of the dichloromethyl(diaryl) sulfonium salt (Scheme 1b). The S–CH(Cl)_2_ bond length was found to be 1.840(7) Å, which is typical of a carbon–sulfur bond and similar to the difluoromethyl(diaryl) sulfonium salt variant, which has a S–CH(F)_2_ bond length of 1.869(2) Å.^20^ This synthetic protocol can be readily scaled to the gram scale without a decrease in the isolated yields. With the sulfonium salts in hand, we investigated their efficacy as a suitable reagent in the *gem*-dichlorocyclopropanation of electron-rich olefins. To investigate the efficacy of these reagents, we selected the *gem*-dichlorocyclopropanation of α-methylstyrene as a model reaction, as this reaction gives moderate yields using CHCl_3_ and phase-transfer catalysis.^21^

We hypothesized that the leaving group ability of the sulfide fragment, (Ar)_2_S, would affect the rate of α-elimination and, hence, dichlorocarbene formation. After extensive exploration of various reaction conditions, the combination of an electron-deficient arene on the sulfonium salt and a bulky organic base, i.e., *N*,*N*-diisopropylethylamine (DIPEA), gave the most promising results. When the model substrate was employed, the desired *gem*-dichlorocyclopropane product **5a** was obtained in 80% yield using sulfonium salt **3a** as the carbene precursor, DIPEA as the base, and dichloromethane as the solvent at ambient temperature (entry 1 in Table 1). Control experiments showed that the sulfonium salt and base were both essential to this transformation (entries 2 and 3); dichloromethane also remained innocent under basic reaction conditions. Decreasing the equivalents of olefin led to decreased yields of compound **5a** despite full consumption of compound **3a** being observed by ^1^H nuclear magnetic resonance (NMR) spectroscopy (entry 4). Similar decreases in the yield were observed when lower equivalents of base were used, as full deprotonation of compound **3a** was not achieved (entry 5). Lowering the temperature from ambient to 0 °C resulted in diminished yields, presumably due to the slower deprotonation of sulfonium salt **3a** (entry 6). The slow addition of compound **3a** via a syringe pump to a mixture of DIPEA and α-methylstyrene was trialed to determine if slow formation of carbene was favorable for higher yields; a lower yield of compound **5a** was obtained as 70% (entry 7).

**Table 1:**
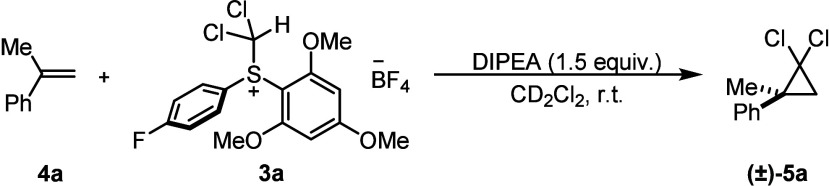
Selected Reaction Optimization with Substrate α-Methylstyrene

entry^a^	deviation from standard conditions	yield of compound **5a** (%)^b^
1	none	80
2	without sulfonium salt	0
3	without base	0
4	1.5 equiv of α-methylstyrene	49
5	1.0 equiv of DIPEA	61
6	0 °C instead of room temperature	55
7	slow addition of compound **3a**	70
8	other sulfonium salts instead of compound **3a**	listed below
9	other bases instead of DIPEA	listed below
10	other solvents instead of CH_2_Cl_2_	listed below

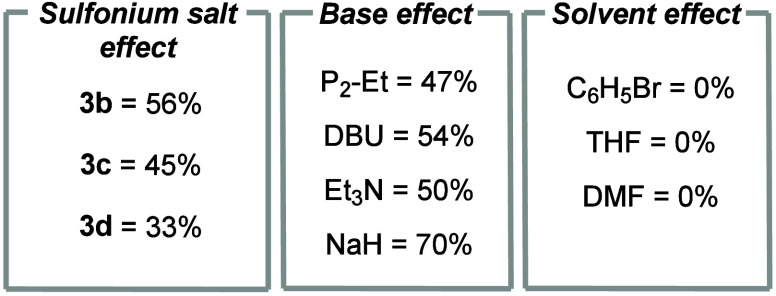

a Standard conditions: compound **3a** (0.0108 mmol), α-methylstyrene (0.0215 mmol), and base (0.0162 mmol) at ambient temperature in 0.5 mL of CD_2_Cl_2_. The reaction time was <5 min.

b NMR yields were obtained using 1.0 equiv of 1,3,5-trimethoxybenzene as an external standard. See the Supporting Information for a complete table.

Sulfonium salt **3a** was the superior carbene precursor; when compounds **3b**–**3d** were used in place of compound **3a**, the yields of compound **5a** significantly decreased to 33–56% (entry 8). We attribute this to the CCl_2_–H bond in compound **3a** being more acidic with respect to compounds **3b**–**3d**. Monitoring the deprotonation of compounds **3a**–**3d** with DIPEA (1.5 equiv) as the base using ^1^H NMR spectroscopy revealed incomplete deprotonation of compounds **3b**–**3d** (17–50% remaining), whereas compound **3a** lead to full conversion to sulfide. DIPEA was found to be the optimal organic base for this reaction; other organic bases furnished lower yields of compound **5a** under the same reaction conditions. It is also evident that the diminished nucleophilicity of the base is important, as triethylamine (Et_3_N) gives a lower yield of compound **5a**. Et_3_N also yields 30% of a quaternary ammonium salt, presumably via a S_N_2 attack at −S–CCl_2_H carbon or by reacting with dichlorocarbene.^22^ Inorganic bases, such as NaH, were found to furnish good yields of compound **5a**; however, 4.0 equiv was required due to the poor solubility of NaH in dichloromethane (entry 9). Moving away from dichloromethane as the solvent resulted in lower yields of compound **5a**. Changing the solvent to bromobenzene led to solubility issues, with sulfonium salt **3a** being completely insoluble. Sulfonium salt **3d**, however, gave 43% of compound **5a** in bromobenzene. Similarly, tetrahydrofuran (THF) and dimethylformamide (DMF) resulted in no reaction being observed (entry 10). From ^1^H NMR spectroscopy, it was evident that some of THF had undergone polymerization. This has been previous reported when using highly electrophilic sulfonium salts in THF.^20^

With optimal conditions in hand, the scope of the *gem*-dichlorocyclopropanation of electron-rich olefins was investigated (Scheme 2). Terminal olefins were efficiently converted to the corresponding *gem*-dichlorocyclopropanes in moderate to good yields; styrene derivatives bearing both electron-donating and -withdrawing groups generally gave products **5b**–**5h** in good yields. For the majority of styrene derivatives, the use of NaH furnished higher yields of the corresponding *gem*-dichlorocyclopropane compared to DIPEA. A different order of addition was trialed, such that sulfonium salt was added last to the base and alkene; however, the yield was not improved. For compound **5c**, −OMe in the *ortho* position decreased the yield in comparison to the *para* and *meta* positions (**5b** and **5d**), potentially due to steric effects. For compound **5b**, changing −OMe for electron-withdrawing −Cl in the *para* position (**5e**) decreased the yield by 20%. *para*-Acetoxystyrene was converted to compound **5h** in good yield (68%), and such substrates are usually unreported with current dichlorocarbene methodologies due to harsh reaction conditions. Our reaction conditions also tolerated a range of di-, tri-, and tetrasubstituted olefins, which were efficiently converted into the corresponding *gem*-dichlorocyclopropanes **5a** and **5i**–**5p** in moderate to high yields (up to 94%). Disubstituted 1,1- and 1,2-monoaryl olefins efficiently participated in the reaction (**5a**, **5k**, and **5l**). *trans*-β-Methylstyrene and *cis*-β-methylstyrene underwent *gem*-dichlorocyclopropanation with compounds **5k** and **5l** being returned with full retention of stereochemistry giving a single diastereomer. Olefins containing saturated nitrogen heterocycles delivered the spirocyclic dichlorocyclopropanes **5i** and **5j** in moderate yields.

**Scheme 2:**
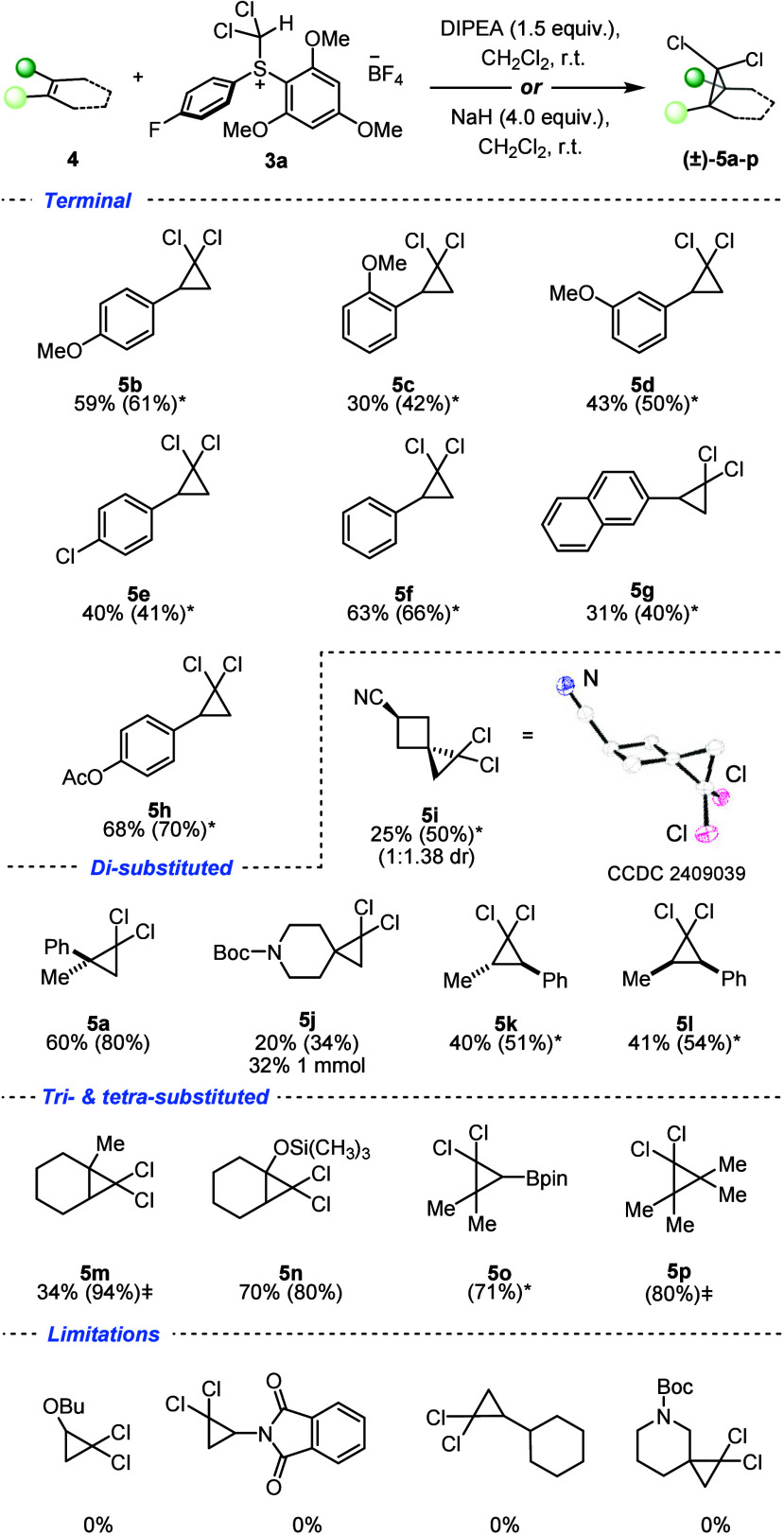
Scope of Olefin *gem*-Dichlorocyclopropanation Yields of compounds **5a**–**5p** in parentheses are NMR yields calculated from ^1^H NMR spectroscopy using 1,3,5-trimethoxybenzene (1.0 equiv) as the internal standard in CD_2_Cl_2_. Compounds were synthesized with NaH (4.0 equiv) as the base. Compounds were too volatile to obtain full characterization and isolated yields, but NMR yields were obtained, with the solid-state structure of compound **5i** with selected atomic labeling; ellipsoids are drawn at the 50% probability level.

When 3-methylenecyclobutene-1-carbonitrile was subjected to the reaction conditions, with NaH (4.0 equiv) as the base, compound **5i** was obtained as a mixture of diastereomers [1:1.38 diastereomeric ratio (dr)] with only the *trans*-diastereomer being isolated in a 25% yield. The relative stereochemistry was confirmed by both nuclear Overhauser effect spectroscopy (NOESY) NMR spectroscopy and single-crystal XRD (see the Supporting Information for details). This further supports the dichlorocarbene mechanism, because the olefin is planar and there is little facial differentiation. Furthermore, silyl enol ethers are also compatible substrates for *gem*-dichlorocyclopropanation, furnishing good yields of product **5n** (70%). Of particular note is that olefins containing the Bpin moiety can be tolerated (**5o**); however, decomposition (either hydrolysis of the pinacol group or protodeboronation) was observed upon purification when a 10% (w/w) AgNO_3_ column was used, leading to unsuccessful isolation.^23^ Olefins yielding cyclopropanes **5m** and **5p** gave high NMR yields; however, volatility of products resulted in unsuccessful isolations. This protocol, however, is not without limitations; for example, *N*-vinyl phthalimide, vinyl butyl ether, and aliphatic monosubstituted olefins failed to undergo *gem*-dichlorocyclopropanation (Scheme 2). As a proof-of-concept reaction, the dichlorocyclopropanation of α-methyl styrene was performed utilizing a flow chemistry setup, which gave an unoptimized yield of 50% (^1^H NMR spectroscopy) using just 1.0 equiv of α-methyl styrene (see the Supporting Information for details).

To probe whether the *gem*-dichlorocyclopropanation proceeds via a concerted singlet carbene mechanism or a stepwise addition mechanism, a competition experiment was performed where both electron-rich and -deficient olefins reacted in the presence of sulfonium salt **3a**. Electron-rich olefin gave 72% conversion to the corresponding *gem*-dichlorocyclopropane, whereas *trans*-β-nitrostyrene (electron-poor olefin) returned unreacted (Scheme 3a). Coupled with this, the full retention of stereochemistry in products **5k** and **5l** further demonstrates a concerted addition of singlet dichlorocarbene to olefin. To probe this mechanism, we conducted density functional theory (DFT) calculations using the m062x/Def2SVP level of theory with the implicit solvent (dichloromethane) modeled. These calculations show that a very thermodynamically favorable α-elimination process occurs from ylide to deliver free dichlorocarbene,^19^ which is in good agreement with our experimental evidence (Scheme 3b). These observations strongly suggest a singlet dichlorocarbene reactivity pathway due to its electrophilic nature and subsequent tendency to react with nucleophilic olefins.

**Scheme 3:**
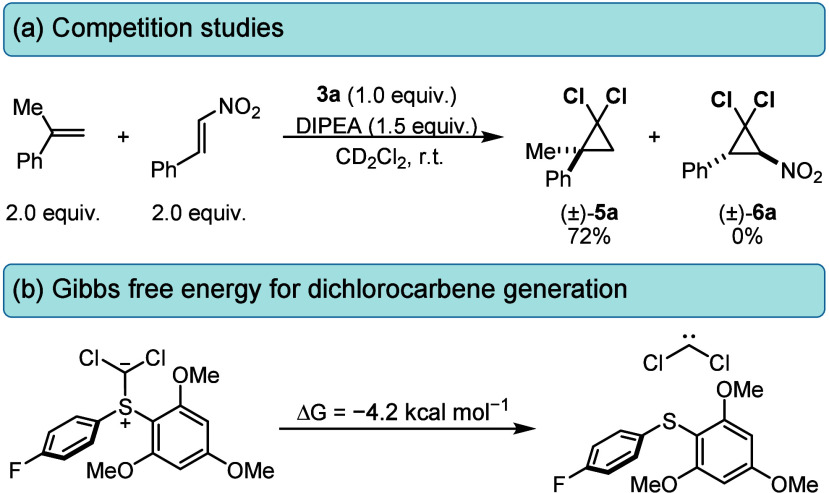
Preliminary Mechanstic Studies: (a) Competition Experiment between α-Methyl Styrene and *trans*-β-Nitrostyrene and (b) Gibbs Free Energies of Dichlorocarbene Formation, with Optimization Using the m062x/Def2SVP Method

Herein, we have designed and developed dichloromethyl(diaryl) sulfonium salts as new precursors for accessing dichlorocarbenes under mild conditions. Not only are these sulfonium salts reactive toward electron-rich olefins to deliver the corresponding *gem*-dichlorocyclopropanes (**5a**–**5p**) under basic conditions, but these salts are also air- and moisture-stable and can be stored for weeks without any sign of degradation. With the new dichloromethyl(diaryl) sulfonium salts, a new protocol was designed to allow access to a variety of *gem*-dichlorocyclopropanes in moderate to good yields. Additionally, our methodology allowed access to *gem*-dichlorocyclopropanes, which could not be synthesized using the pre-existing dichlorocarbene methodologies. Highly substituted olefins were tolerated well under the reaction conditions as well as functionalities, such as nitriles, carbamates, and silyl ethers.

## Data Availability

The data underlying this study are available in the published article and its Supporting Information.

## Supporting Information Available

Experimental procedures, characterization of products, NMR spectra, single-crystal X-ray data, and DFT calculations (PDF)
