# Supplementary material for: Dichloromethyl(diaryl) Sulfonium Salts as gem-Dichlorocyclopropanation Reagents
Source: Org Lett. 2025 Feb 3;27(6):1402–6. doi: 10.1021/acs.orglett.4c04717 (PMC11834146; doi:10.1021/acs.orglett.4c04717)
Supplement: Supplementary file 1 — ol4c04717_si_001.pdf [file ol4c04717_si_001.pdf]

Supporting Information

Dichloromethyl(diaryl) Sulfonium Salts as *gem*-Dichlorocyclopropanation Reagents

Bethany Jane Moore[a] **and Darren Willcox**[a*]

[a]**Department of Chemistry, University of Manchester, Oxford Road, M13 9PL**

[*]**darren.willcox@manchester.ac.uk**

Contents

General Considerations 2
General Crystallographic Methods 3
XRD Experimental Parameters 4
General Experimental Procedures 5
Flow Chemistry Experiments 8
Competition Experiment 9
Scale-up Reaction 9
Density Functional Theory (DFT) Calculations 10
Optimised Geometries 10
Characterisation Data of *gem*-Dichlorocyclopropanes 14
References 26
NMR Spectra of Compounds 27

General Considerations

Starting materials sourced from commercial suppliers were used as supplied unless stated otherwise. Compounds **1b** and **1c** were purchases and used as supplied; **1b** was sourced from Thermo Scientific Alfa Aesar and **1c** was sourced from Apollo Scientific. Dry solvents, where necessary, were obtained from the Solvent Purification System (SPS), which were obtained by passing through a column of anhydrous alumina. The removal of solvents *in vacuo* was achieved using a Büchi rotary evaporator with a bath temperature of 30 °C. Reactions requiring anhydrous conditions were run under an atmosphere of dry nitrogen, from a cylinder. Needles and glassware were dried in an oven (200 °C) overnight and glassware was flame-dried under vacuum prior to use. Liquid reagents, solutions or solvents were added *via* syringe using rubber septa and solid reagents were added under a flow of nitrogen. Thin layer chromatography (TLC) was performed using commercially available Macherey-Nagel POLYGRAM® SIL G/UV254 0.2 mm silica gel plates. Visualisation of TLC plates was achieved by staining with a potassium permanganate solution and a heat gun, or by UV fluorescence (254 nm). Purification by flash column chromatography was performed on silica gel (SUPELCO 40-63 µM, 230-400 mesh particle size). For preparative TLC, 20 x 20 cm glass supported plates were used with a layer thickness of 500 µM, 20-40 µM particle size and 60 Å pore medium pore size. 10% w/w $AgNO_3$ doped silica gel was prepared in accordance with literature procedures.[1] Infrared spectra were recorded using a Perkin Elmer Spectrum One FT-IR Spectrometer as solids or neat films in the range of 4000-600 cm^{-1}. NMR spectra were recorded using a Bruker AV-III HD 500 (Prodigy Cryoprobe, 500 MHz) NMR spectrometer or an AV-III HD 400 (Prodigy Cryoprobe, 400 MHz) NMR spectrometer. Chemical shifts (δ) are reported in parts per million (ppm) from high to low frequency and are given to the nearest 0.01 ppm. Coupling constants (*J*) are given in Hz to the nearest 0.10 Hz. The signals' multiplicity are abbreviated as s (singlet), d (doublet), t (triplet), q (quartet) or m (multiplet). ^{1}H and ^{13}C NMR are referenced to the appropriate residual solvent peak and all spectra were obtained at 298 K unless stated otherwise. ^{1}H and ^{13}C NMR spectra were assigned, were possible, using COSY, HSQC and HMBC experiments. Mass spectra were determined by the University of Manchester mass spectrometry service using a Thermo Q Exactive Focus Orbitrap System with a quadrupole and ion trap. Samples were ran using either ASAP, APCI or HESI. For EI, samples were ran using Shimadzu GCMS-QP210 SE with a single quadrupole. All calculations were performed using the GaussianG09W (version g09, rev.d01) software package. All geometry optimisations and frequency calculations were performed without any restrictions at the m062x def2svp level of theory for all atoms. The stationary points were verified to be real by the absence of imaginary frequencies.[2-4]

General Crystallographic Methods

Single crystal X-ray diffraction data for **5i** crystals were collected using a Rigaku XtaLAB Synergy-S dual wavelength microfocus diffractometer using rotating-anode X-ray tube of CuKα (λ = 1.54146 Å) radiation, equipped with a 4-circle quarter-χ goniometer, PhotonJet-S microfocus optics, a Hypix-6000HE detector and an Oxford Cryosystems 800 plus nitrogen flow gas system, at a temperature of 100K. This data can be obtained free of charge from the Cambridge Crystallographic Data Centre *via* deposition number 2409039.

Single crystal X-ray diffraction data for **3b** crystals were collected on the Rigaku Oxford Diffraction Supernova, four-circle diffractometer equipped with CCD plate detector, using micro-focus sealed X-ray tube of Mo Kα (λ = 0.71073 Å) radiation and an Oxford Cryosystems 800 plus nitrogen flow gas system, at a temperature of 100K. This data can be obtained free of charge from the Cambridge Crystallographic Data Centre *via* deposition number 2409038.

Data were collected and reduced using CrysAlisPro v43.[5] The crystal structure was solved by Intrinsic Phasing using the ShelXT program. The absorption correction is performed using Empirical absorption correction using spherical harmonics implemented in SCALE3 ABSPACK scaling algorithm and Analytical numeric absorption correction by identifying crystal faces. The crystal structures were solved and refined against all $F^2>2\sigma$ values using the SHELX and Olex2 suite of programmes.[6,7]

XRD Experimental Parameters

Identification code	**3b**	**5i**
Empirical formula	$BC_{16}Cl_2F_4O_3SH_{17}$	$C_7H_7Cl_2N$
Formula weight	447.07	176.04
Temperature/K	99.9(3)	99.99(11)
Crystal system	monoclinic	orthorhombic
Space group	C c	$Pna2_1$
a/Å	12.1482(7)	12.4160(17)
b/Å	17.6199(8)	5.8100(5)
c/Å	9.7028(5)	11.0105(15)
α/°	90	90
β/°	108.664(5)	90
γ/°	90	90
Volume/$Å^3$	1967(19)	794.26(17)
Z	4	4
$\rho_{calc}g/cm^3$	1.509	1.472
μ/mm^{-1}	0.486	6.691
F(000)	912.0	360.0
Crystal size/mm^3	0.408 × 0.341 × 0.238	0.259 × 0.086 × 0.018
Radiation	Mo Kα (λ = 0.71073)	Cu Kα (λ = 0.71073)
2Θ range for data collection/°	6.898 to 52.76	14.268 to 172.15
Index ranges	15 ≤ h ≤ 12, -22 ≤ k ≤ 19, -12 ≤ l ≤ 11	-15 ≤ h ≤ 15, -4 ≤ k ≤ 7, -14 ≤ l ≤ 14
Reflections collected	4716	5325
Independent reflections	2687 [R_{int} = 0.0294, R_{sigma} = 0.0506]	1689 [R_{int} = 0.0428, R_{sigma} = 0.0556]
Data/restraints/parameters	2687/156/281	1689/1/91
Goodness-of-fit on F^2	1.049	1.085
Final R indexes [I>=2σ (I)]	R_1 = 0.0535, wR_2 = 0.1275	R_1 = 0.0730, wR_2 = 0.2014
Final R indexes [all data]	R_1 = 0.0589, wR_2 = 0.1342	R_1 = 0.0815, wR_2 = 0.2241
Largest diff. peak/hole / e $Å^{-3}$	1.59/-0.38	0.77/-0.46

General Experimental Procedures

General Procedure A: Synthesis of Methyl(aryl) Sulfoxides

Methyl(aryl) sulfoxides were synthesised according to a literature procedure. Sulfide (1.0 equiv.) was dissolved in anhydrous CH_2Cl_2 (0.5 M), and *m*-CPBA (1.05 equiv.) was added at 0 °C under an atmosphere of N_2. The resulting reaction mixture was stirred overnight at room temperature. The organic layer was washed with 1 M aq. NaOH (3 x 20 mL) and H_2O (3 x 20 mL). The combined organic extracts were washed with brine, dried over anhydrous $MgSO_4$, filtered and concentrated *in vacuo*. The compounds were used without any further purification.[8]

General Procedure B: Synthesis of Dichloromethyl(aryl) Sulfoxides

Dichloromethyl(aryl) sulfoxides were synthesised according to a literature procedure. *N*-Chlorosuccinimide (2.05 equiv.) was added to a solution of methyl(aryl) sulfoxide (1.0 equiv.) in anhydrous THF (0.3 M) at 0 °C under an atmosphere of N_2. The resulting reaction mixture was stirred overnight at room temperature. CH_2Cl_2 was added to the reaction mixture, which was washed with H_2O (3 x 20 mL). The combined organic extracts were washed with brine, dried over anhydrous $MgSO_4$, filtered, and concentrated *in vacuo*. The residue was purified by silica gel column chromatography to give the desired dichloromethyl(aryl) sulfoxide.[8]

General Procedure C: Synthesis of Dichloromethyl(diaryl) Sulfonium Salts

Sulfonium salts were synthesised according to a general literature procedure. To a stirring solution of dichloromethyl(aryl) sulfoxide (1.0 equiv.) and arene (1.1 equiv.) in a Schlenk flask was added trifluoromethanesulfonic acid anhydride (Tf_2O, 1.1 equiv.) in anhydrous Et_2O (0.1 M) at -78 °C, and under an atmosphere of N_2. The reaction mixture was stirred for a further 2 hours at -78 °C. After 2 hours, the mixture was warmed to room temperature and stirred for 30 minutes. The reaction mixture formed two layers, with the sulfonium salt settling at the bottom of the Schlenk flask. The upper layer of the reaction mixture was removed *via* a syringe, further Et_2O was added, and the mixture was stirred again. This procedure was repeated three times. CH_2Cl_2 was added to the remaining oil and extracted with aqueous $NaBF_4$ solution (1 M, 20 mL × 4). The organic layer was dried over $MgSO_4$, filtered and concentrated *in vacuo*. It was necessary to remove the CH_2Cl_2 quickly and aggressively to ensure a fluffy solid formed.[9]

General Procedure D: Synthesis of *gem*-Dichlorocyclopropanes using DIPEA

Sulfonium salt (1.0 equiv.) and olefin (2.0 equiv.) were dissolved in CH_2Cl_2 (0.04 M) in a vial. DIPEA (1.5 equiv.) was added to the mixture resulting in a yellow solution. The reaction mixture was quenched with H_2O (5 mL) after 5 minutes and the organic layer was dried over anhydrous $MgSO_4$. The solution

was filtered and concentrated with sparging to leave a residue, which was purified by silica gel column chromatography (5.0 cm of silica in a glass pipette) to give the desired *gem*-dichlorocyclopropane.

General Procedure E: Synthesis of *gem*-Dichlorocyclopropanes using NaH

Sulfonium salt (1.0 equiv.) and olefin (2.0 equiv.) were dissolved in CH_2Cl_2 (0.04 M) in a vial. NaH (4.0 equiv., 60% dispersion in mineral oil) was added to the solution resulting in a yellow solution. The reaction mixture was quenched with H_2O (5 mL) after 5 minutes and the organic layer was dried over anhydrous $MgSO_4$. The solution was filtered and concentrated with sparging to leave a residue, which was purified by silica gel column chromatography (5.0 cm of silica in a glass pipette) to give the desired *gem*-dichlorocyclopropane.

Table of Optimisations

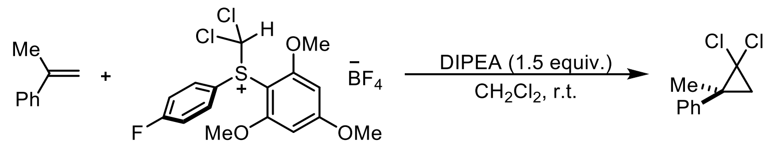

4a **3a** **(±)-5a**

Entry[a]	*Salt*	*Base*	*Base (equiv.)*	*Olefin (equiv.)*	*Temp (°C)*	*Solvent*	*Yield of* ***5a*** *(%)*[b]
1	**3a**	DBU	1.0	1.0	r.t.	CH_2Cl_2	44
2	**3a**	DBU	1.0	2.0	r.t.	CH_2Cl_2	54
3	**3a**	Et_3N	1.0	2.0	r.t.	CH_2Cl_2	50
4	**3a**	NaH	4.0	2.0	r.t.	CH_2Cl_2	70
5	**3a**	P_2-Et	1.0	2.0	r.t.	CH_2Cl_2	47
6	**3a**	DIPEA	1.0	2.0	r.t.	CH_2Cl_2	61
7	**3a**	DIPEA	1.0	1.5	r.t.	CH_2Cl_2	48
8	**3a**	DIPEA	1.0	2.0	r.t.	THF	0
9	**3a**	DIPEA	1.0	2.0	r.t.	C_6H_5Br	0[c]
10	**3a**	DIPEA	1.0	2.0	r.t.	DMF	0
10	**3a**	DIPEA	1.2	2.0	r.t.	CH_2Cl_2	67
**11**	**3a**	**DIPEA**	**1.5**	**2.0**	**r.t.**	$\mathbf{CH_2Cl_2}$	**80**
12	**3a**	DIPEA	1.5	1.5	r.t.	CH_2Cl_2	49
13	**3a**	DIPEA	1.5	2.5	r.t.	CH_2Cl_2	64
14	**3a**	DIPEA	1.5	2.0	0	CH_2Cl_2	55
15	**3a**	DIPEA	1.5	2.0	-78	CH_2Cl_2	40
16	**3a**	DIPEA	1.5	2.0	40	CH_2Cl_2	68
17	None	DIPEA	1.5	2.0	r.t.	CH_2Cl_2	0
18	**3a**	None	None	2.0	r.t.	CH_2Cl_2	0
19	**3a**	DIPEA	1.5	None	r.t.	CH_2Cl_2	0
20	**3a**	DIPEA	1.5	2.0	r.t.	CH_2Cl_2	70[d]
21	**3b**	DIPEA	1.5	2.0	r.t.	CH_2Cl_2	56
22	**3c**	DIPEA	1.5	2.0	r.t.	CH_2Cl_2	51
23	**3d**	DIPEA	1.5	2.0	r.t.	CH_2Cl_2	33

Table S1. Full optimisation table. [a] Standard conditions: **3a** (0.0108 mmol) α-methyl styrene (0.0215 mmol), DIPEA (0.0162 mmol) at ambient temperature in 0.5 mL of CD_2Cl_2.Reaction time was <5 min. [b] NMR yields were obtained using 1.0 equiv. of 1,3,5-trimethoxybenzene as an external standard. [c] Sulfonium salt **3a** did not dissolve in C_6H_5Br. [d] Sulfonium salt was added to a mixture of olefin and base *via* syringe pump over 2 hours.

Flow Chemistry Experiments

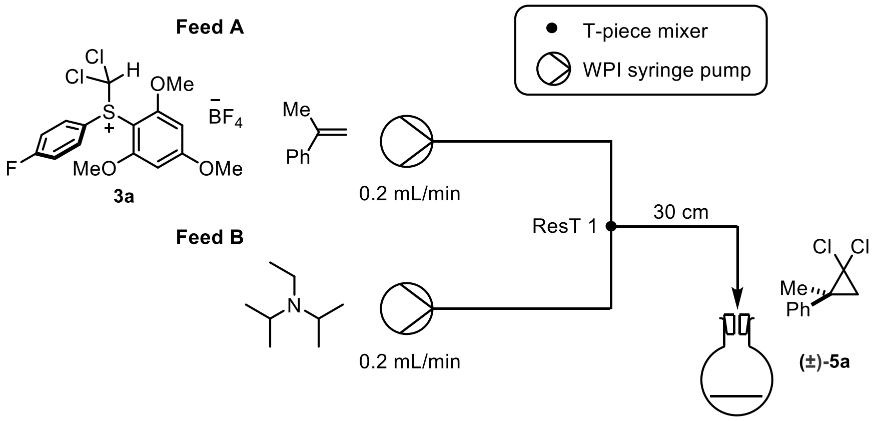

A WPI syringe pump was implemented to pump **3a** (1.0 equiv., 20.7 mg, 0.0430 mmol, 0.02 M) and α-methyl styrene (1.0 equiv., 5.08 mg, 0.0430 mmol, 0.02 M) as **feedstock A** and DIPEA (1.5 equiv., 8.34 mg, 0.0645 mmol, 0.03 M) as **feedstock B** at flow rates of 0.2 mL/min. 2 mL syringes were fitted for the syringe pump. A T-piece mixer was used for ResT 1 (residence time 1) which allowed instantaneous mixing of the two reactant feeds. PTFE tubing was used with an internal diameter of 0.18 mm. Once the reaction had occurred in the T-piece mixer, the *gem*-dichlorocyclopropane **5a** was collected into a vial. A 400 µL aliquot was taken and analysed by ^{1}H NMR to give a 50% crude yield.

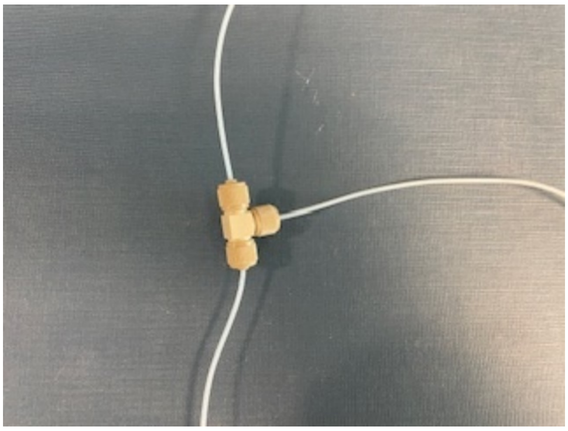

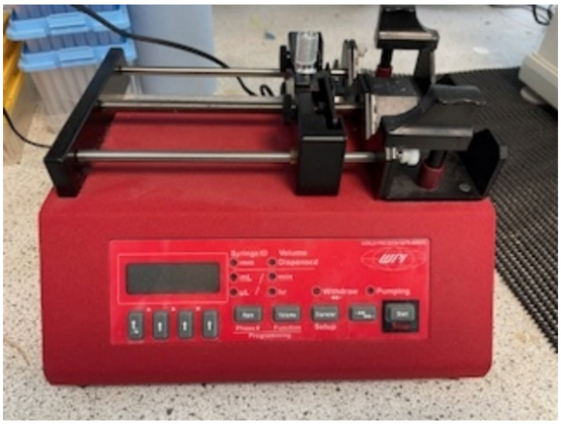

Scheme S1. a) the set-up of the T-piece mixer connecting **feeds A** and **B** which was connected to the output tubing. b) the WPI syringe pump used to deliver **feedstock A** and **feedstock B.**

Competition Experiment

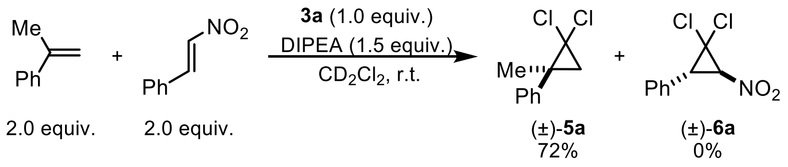

Dichloromethyl(diaryl) sulfonium salt **3a** (5.00 mg, 0.0108 mmol, 1 equiv.) was added to α-methyl styrene (2.54 mg, 0.0215 mmol, 2.80 µL, 2 equiv.) and *trans*-β-nitrostyrene (3.21 mg, 0.0215 mmol, 2 equiv.) in CD_2Cl_2 (0.02 M, 0.5 mL). DIPEA (2.09 mg, 0.0162 mmol, 2.82 µL) was added in one portion. The crude mixture was analysed by ^{1}H NMR and yields were determined by the addition of 1,3,5-trimethoxybenzene (1.82 mg, 0.0108 mmol, 1 equiv.) as an external standard. The ^{1}H NMR yield of **5a** was determined as 72%, whereas no conversion to **6a** was observed.

Scale-up Reaction

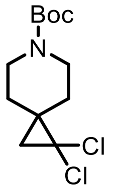

5j

General Procedure D: compound **3a** (0.465 g, 1.00 mmol, 1 equiv.) and *tert*-butyl 4-methylenepiperidine-1-carboxylate (0.395 g, 2.00 mmol, 0.395 mL, 2 equiv.) were dissolved in CH_2Cl_2 (0.04 M, 25 mL). DIPEA (0.194 g, 1.50 mmol, 0.261 mL, 1.5 equiv.) was then added in one portion. The crude mixture was purified by preparative TLC to yield the title compound **5j** as a white solid (92 mg, 32%); R_f 0.6 (7:1 pentane: Et_2O); m.p. 86 – 88 °C; ^{1}H NMR (700 MHz, CD_2Cl_2) δ 3.57 (ddd, *J* = 13.0, 7.2, 3.9 Hz, 2H), 3.39 (ddd, *J* = 13.2, 7.9, 3.6 Hz, 2H), 1.79 (ddd, *J* = 13.7, 7.9, 3.7 Hz, 2H), 1.63 (ddd, *J* = 13.6, 7.2, 3.6 Hz, 2H), 1.45 (s, 9H), 1.31 (s, 2H); ^{13}C NMR (176 MHz, CD_2Cl_2) δ 155.0, 79.7, 67.1, 43.5, 43.0, 32.9, 32.0, 31.5, 28.5 HRMS: (APCI) Calculated for $[C_{12}H_{19}Cl_2O_2N+H]^+$: 280.0854. Found $[M+H]^+$: 280.0866

Density Functional Theory (DFT) Calculations

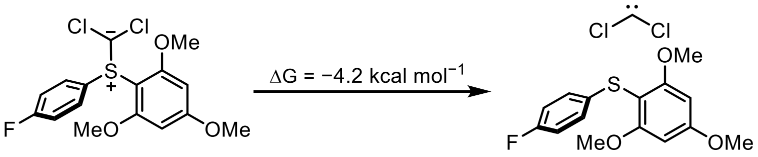

All calculations were performed using the GaussianG09W (version g09, rev.d01) software package. All geometry optimisations and frequency calculations were performed without any restrictions at the m062x def2svp level of theory for all atoms and with implicit solvent (dichloromethane). The stationary points were verified to be real by the absence of imaginary frequencies. The Gibbs free energy of the carbene formation was calculated by comparing the energy of the (diaryl)sulfide and the dichlorocarbene with that of dichloromethyl(diaryl) sulfonium ylide. Dissociation of the C-S bond was observed to form thermodynamically more stable carbene (–4.2 kcal mol^{-1}).[2-4]

Optimised Geometries

Dichloromethyl(diaryl) Sulfonium Ylide

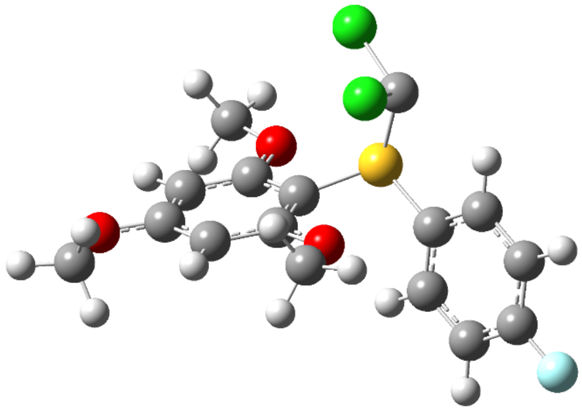

Zero-point correction= 0.282230 (Hartree/Particle)
Thermal correction to Energy= 0.305255
Thermal correction to Enthalpy= 0.306200
Thermal correction to Gibbs Free Energy= 0.228653
Sum of electronic and zero-point Energies= -2260.911703
Sum of electronic and thermal Energies= -2260.888677
Sum of electronic and thermal Enthalpies= -2260.887733
Sum of electronic and thermal Free Energies= -2260.965279

S	0.52068500	-1.27453100	0.52096400
Cl	-0.73322200	-3.38695100	-0.89856900
O	0.43270900	1.43756800	-0.84251900

O	-4.22007000	2.18982600	0.08732400
C	-0.78499400	-0.06549700	0.47024100
C	-0.74272400	1.11174900	-0.30129500
C	0.49517200	2.44332800	-1.83108500
H	1.52041800	2.42655400	-2.21835100
H	-0.20922600	2.23432400	-2.65131400
H	0.28728700	3.43744300	-1.40597700
C	2.03276100	-0.31715400	0.58074300
C	-3.07564400	1.49930700	0.17929200
C	2.16395900	0.60624500	1.61884600
H	1.35011900	0.76889800	2.32907000
C	-1.89004400	1.90103600	-0.44603500
H	-1.85109300	2.81131300	-1.03721400
C	3.06464300	-0.54392500	-0.32608900
H	2.94831000	-1.27955900	-1.12122400
C	0.65566400	-2.23185900	-0.97965900
C	-3.13481700	0.33124300	0.95489900
H	-4.08190900	0.06596800	1.41931200
O	-1.95865500	-1.58974700	1.79945200
C	-1.99407700	-0.45170400	1.09561400
C	-4.25772800	3.35776800	-0.70757800
H	-5.28187300	3.74151900	-0.64428300
H	-3.56183400	4.12223900	-0.32841000
H	-4.02054500	3.13230500	-1.75904100
C	4.36145900	1.11448900	0.81972400
C	-3.15995200	-2.10008800	2.34131700
H	-2.89918800	-3.04554300	2.82927200
H	-3.58556100	-1.41201700	3.08768600
H	-3.90021600	-2.28972100	1.54884600
C	4.24551400	0.18665200	-0.20579600
H	5.07386800	0.04278200	-0.90024800
C	3.34143600	1.33690200	1.73824800
H	3.47925200	2.07204200	2.53179800
Cl	0.43989100	-1.20988200	-2.44084700
F	5.49246700	1.81533100	0.93248200

Diarylsulfide

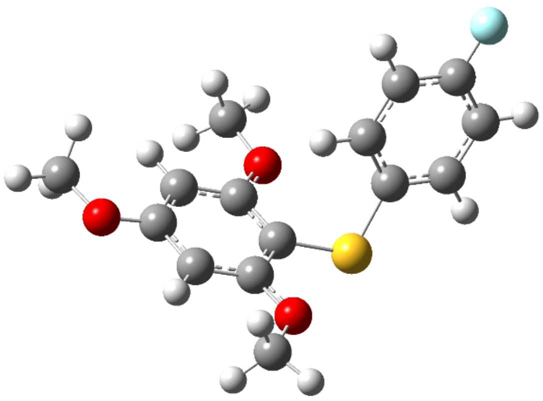

Zero-point correction= 0.274775 (Hartree/Particle)
Thermal correction to Energy= 0.293699
Thermal correction to Enthalpy= 0.294643
Thermal correction to Gibbs Free Energy= 0.226373
Sum of electronic and zero-point Energies= -1302.863263
Sum of electronic and thermal Energies= -1302.844339
Sum of electronic and thermal Enthalpies= -1302.843395
Sum of electronic and thermal Free Energies= -1302.911665

S	-0.73400300	1.28465400	-1.35999300
O	0.05839300	-1.52779300	-1.35708100
O	4.23801700	-0.91725100	0.95409100
C	0.72416500	0.59250300	-0.64345400
C	0.99515000	-0.78477100	-0.74982600
C	0.22965600	-2.92573400	-1.44119600
H	-0.65630300	-3.31352600	-1.95620300
H	1.12769000	-3.18793600	-2.02239800
H	0.29478700	-3.38236900	-0.44092800
C	-2.04867200	0.55229600	-0.40948700
C	3.08268600	-0.48708100	0.41648900
C	-1.85410300	-0.13074300	0.79520100
H	-0.84834900	-0.25402900	1.20094000
C	2.16699200	-1.33676900	-0.21241700
H	2.35770700	-2.40322900	-0.29030300
C	-3.34840600	0.70590800	-0.91116700
H	-3.51115000	1.23050500	-1.85551000
C	2.84131800	0.88804100	0.52579300
H	3.58861300	1.50076100	1.02462800
O	1.36924400	2.73250600	0.07051200
C	1.66568800	1.42579200	0.00063700
C	4.56025400	-2.28793500	0.87155200
H	5.53499300	-2.40893100	1.35783000
H	3.81667400	-2.90769900	1.39741800
H	4.63615200	-2.62049800	-0.17577100
C	-4.21944100	-0.48774800	0.97224000
C	2.28752200	3.60564600	0.69091500
H	1.85470200	4.60991400	0.62067800
H	2.43579100	3.34871700	1.75171500
H	3.26001200	3.59599100	0.17399700
C	-4.44104000	0.18962000	-0.21944200
H	-5.45837600	0.30071000	-0.59716400
C	-2.94253800	-0.65728400	1.48902000
H	-2.80956800	-1.19297300	2.43014400
F	-5.26666100	-0.99333600	1.63905500

Dichlorocarbene

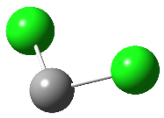

Zero-point correction= 0.004353 (Hartree/Particle)
Thermal correction to Energy= 0.007718
Thermal correction to Enthalpy= 0.008662
Thermal correction to Gibbs Free Energy= -0.021965
Sum of electronic and zero-point Energies= -958.020634
Sum of electronic and thermal Energies= -958.017269
Sum of electronic and thermal Enthalpies= -958.016325
Sum of electronic and thermal Free Energies= -958.046953

Cl	1.39991800	-0.14608700	0.00000000
C	0.00000000	0.83196800	0.00000000
Cl	-1.39991800	-0.14754900	0.00000000

Characterisation Data of *gem*-Dichlorocyclopropanes

(1a): 1-fluoro-4-(methylsulfinyl)benzene

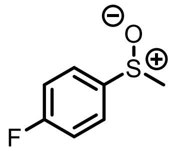

1a

General Procedure A: (4-fluorophenyl)(methyl)sulfane (5.00 g, 35.0 mmol, 4.28 mL, 1 equiv.) and *m*CPBA (6.35 g, 36.8 mmol, 1.05 equiv.) in CH_2Cl_2 (0.5 M, 70 mL). The title compound **1a** was yielded without further purification as a pale-yellow oil (3.60 g, 65%). ^{1}H NMR (500 MHz, CD_2Cl_2) δ 7.69 – 7.61 (m, 2H), 7.27 – 7.20 (m, 2H), 2.68 (s, 3H); ^{13}C NMR (101 MHz, CD_2Cl_2) δ 164.5 (d, *J* = 249.4 Hz), 142.2 (d, *J* = 2.9 Hz), 126.1 (d, *J* = 8.8 Hz), 116.8 (d, *J* = 22.5 Hz), 44.5; ^{19}F NMR (471 MHz, CD_2Cl_2) δ -109.54 – -109.64 (m, 1F). The data were in accordance with the ones reported in literature.[10]

(1d): 1-methoxy-4-(methylsulfinyl)benzene

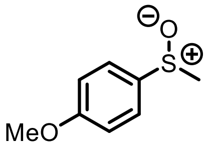

1d

General Procedure A: (4-methoxyphenyl)(methyl)sulfane (1.00 g, 6.48 mmol, 1 equiv.) and *m*CPBA (1.17 g, 6.80 mmol, 1.05 equiv.) in CH_2Cl_2 (0.5 M, 13 mL). The title compound **1d** was yielded without further purification as a pale-yellow oil (0.460 g, 42%); ^{1}H NMR (400 MHz, CD_2Cl_2) δ 7.64 – 7.53 (m, 2H), 7.04 (d, *J* = 8.8 Hz, 2H), 3.84 (s, 3H), 2.65 (s, 3H); ^{13}C NMR (101 MHz, CD_2Cl_2) δ 162.3, 137.5, 125.7, 115.1, 55.9, 44.4. The data were in accordance with the ones reported in literature.[10]

(2a): 1-((dichloromethyl)sulfinyl)-4-fluorobenzene

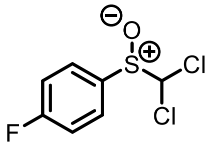

2a

General Procedure B: sulfoxide **1a** (3.52 g, 22.3 mmol, 1 equiv.) and *N*-chlorosuccinimide (5.97 g, 44.7 mmol, 2.01 equiv.) in THF (0.3 M, 72 mL). The crude mixture was purified by silica gel chromatography to yield the title compound **2a** as a yellow oil (3.54 g, 70%); R_f 0.6 (7:3 Et_2O:petroleum ether); ^{1}H NMR (500 MHz, CD_2Cl_2) δ 7.76 – 7.68 (m, 2H), 7.26 – 7.17 (m, 2H), 6.13 (s, 1H); ^{13}C NMR (126 MHz, CD_2Cl_2)

δ 166.1 (d, *J* = 253.6 Hz), 134.1 (d, *J* = 2.9 Hz), 129.6 (d, *J* = 9.3 Hz), 116.8 (d, *J* = 22.8 Hz), 83.5 (d, *J* = 2.9 Hz); ^{19}F NMR (471 MHz, CD_2Cl_2) δ -106.06 (dq, *J* = 7.8, 4.1 Hz). IR ν_{max}/cm^{-1}: 3099, 3069, 2054, 1490, 1083, 834, 815. HRMS: (APCI) Calculated for $[C_7H_5Cl_2FOS+H]^+$: 226.9495. Found $[M+H]^+$: 226.9492.

(2b): ((dichloromethyl)sulfinyl)benzene

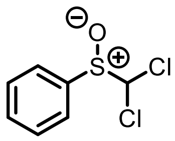

2b

General Procedure B: (methylsulfinyl)benzene (0.800 g, 5.06 mmol, 1 equiv.) and *N*-chlorosuccinimide (1.35 g, 10.1 mmol, 2.01 equiv.) in THF (0.3 M, 17 mL). The crude mixture was purified by silica gel chromatography to yield the title compound **2b** as a yellow oil (0.901 g, 85%); R_f 0.6 (7:3 Et_2O:petroleum ether); ^{1}H NMR (500 MHz, $CDCl_3$) δ 7.82 – 7.77 (m, 2H), 7.67 – 7.57 (m, 1H), 7.61 – 7.54 (m, 2H), 6.17 (s, 1H); ^{13}C NMR (126 MHz, $CDCl_3$) δ 138.2, 133.2, 129.1, 126.8, 83.2. The data were in accordance with the ones reported in literature.[8]

(2c): 1-((dichloromethyl)sulfinyl)-4-methylbenzene

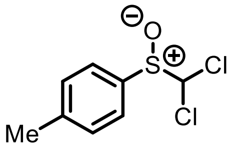

2c

General Procedure B: 1-methyl-4-(methylsulfinyl)benzene (1.00 g, 6.64 mmol, 1 equiv.) and *N*-chlorosuccinimide (1.77 g, 13.3 mmol, 2.01 equiv.) in THF (0.3 M, 22 mL). The title compound **2c** was yielded without further purification as a pale-yellow oil (0.780 g, 53%); ^{1}H NMR (500 MHz, CD_2Cl_2) δ 7.66 (d, *J* = 8.0 Hz, 2H), 7.40 (d, *J* = 8.0 Hz, 2H), 6.17 (s, 1H), 2.45 (s, 3H); ^{13}C NMR (126 MHz, CD_2Cl_2) δ 144.5, 135.6, 130.1, 126.9, 83.8, 21.8. The data were in accordance with the ones reported in literature.[8]

(2d): 1-((dichloromethyl)sulfinyl)-4-methoxybenzene

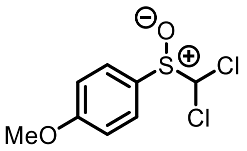

2d

General Procedure B: sulfoxide **1d** was employed (0.460 g, 2.69 mmol, 1 equiv.) and *N*-chlorosuccinimide (0.736 g, 5.51 mmol, 2.01 equiv.) in THF (0.3 M, 9 mL). The title compound **2d** was yielded without further purification as a pale-yellow oil (0.32 g, 50%); ^{1}H NMR (400 MHz, CD_2Cl_2) δ 7.71 (d, *J* = 8.9 Hz, 2H), 7.11 – 7.05 (m, 2H), 6.17 (s, 1H), 3.88 (s, 3H); ^{13}C NMR (101 MHz, CD_2Cl_2) δ 164.1, 128.9 (2C), 114.9, 83.8, 56.1. The data were in accordance with the ones reported in literature.[8, 11]

(3a): (dichloromethyl)(4-fluorophenyl)(2,4,6-trimethoxyphenyl)sulfonium tetrafluoroborate

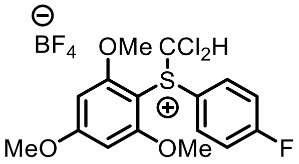

3a

General Procedure C: 1-((dichloromethyl)sulfinyl)-4-fluorobenzene **2a** (1.59 g, 7.00 mmol, 1 equiv.), 1,3,5-trimethoxybenzene (1.30 g, 7.70 mmol, 1.1 equiv.) and Tf_2O (2.17 g, 7.70 mmol, 1.3 mL, 1.1 equiv.) in Et_2O (0.1 M, 70 mL). The title compound **3a** was yielded without further purification as a pale-pink solid (2.77 g, 85%); m.p. 114 – 116 °C; ^{1}H NMR (400 MHz, CD_2Cl_2) δ 8.08 (s, 1H), 8.07 – 8.03 (m, 2H), 7.47 – 7.38 (m, 2H), 6.32 (s, 2H), 4.06 (s, 6H), 3.95 (s, 3H); ^{13}C NMR (101 MHz, CD_2Cl_2) δ 170.5, 167.3 (d, *J* = 260.2 Hz), 163.3, 135.3 (d, *J* = 10.2 Hz), 119.6 (d, *J* = 3.3 Hz), 119.3 (d, *J* = 23.4 Hz), 93.2, 86.8, 78.3, 57.9, 57.1; ^{19}F NMR (376 MHz, CD_2Cl_2) δ -99.48 (tt, *J* = 8.0, 4.7 Hz, 1F), -151.71 (s, 1F), -153. (s, 3F). IR ν_{max}/cm^{-1}: 2221, 1164, 819, 796. HRMS: (APCI) Calculated for $[C_{16}H_{16}O_3Cl_2SF\text{-}BF_4]^+$: 377.0176. Found $[M\text{-}BF_4]^+$: 377.0168.

(3b): (dichloromethyl)(phenyl)(2,4,6-trimethoxyphenyl)sulfonium tetrafluoroborate

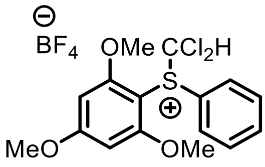

3b

General Procedure C: ((dichloromethyl)sulfinyl)benzene **2b** (0.260 g, 1.25 mmol, 1 equiv.), 1,3,5-trimethoxybenzene (0.231 g, 1.38 mmol, 1.1 equiv.) and Tf_2O (0.389 g, 1.38 mmol, 0.232 mL, 1.1 equiv.) in Et_2O (0.1 M, 13 mL). The title compound **3b** was yielded without further purification as a pale-purple solid (0.521 g, 90%). An anti-solvent recrystallisation in 3:2 *n*-hexane and CH_2Cl_2 gave single crystals suitable for single crystal XRD. M.p. 122 – 125 °C; ^1H NMR (500 MHz, CD_2Cl_2) δ 8.11 (s, 1H), 8.01 – 7.95 (m, 2H), 7.84 – 7.76 (m, 1H), 7.74 – 7.66 (m, 2H), 6.34 (s, 2H), 4.05 (s, 6H), 3.96 (s, 3H); ^{13}C NMR (126 MHz, CD_2Cl_2) δ 170.4, 163.3, 135.8, 132.0, 131.6, 124.3, 93.2, 86.8, 78.0, 57.8, 57.1; ^{19}F NMR (376 MHz, CD_2Cl_2) δ -153.02 (s, 1F), -153.07 (s, 3F). IR ν_{max}/cm^{-1}: 2167, 2109, 1592, 1574, 822, 795. HRMS: (ESI) Calculated for $[C_{16}H_{17}O_3Cl_2S\text{-}BF_4]^+$: 359.0270. Found $[M\text{-}BF_4]^+$: 359.0266.

(3c): (dichloromethyl)(*p*-tolyl)(2,4,6-trimethoxyphenyl)sulfonium tetrafluoroborate

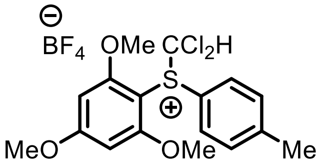

3c

General Procedure C: 1-((dichloromethyl)sulfinyl)-4-methylbenzene **2c** (0.197 g, 0.881 mmol, 1 equiv.), 1,3,5-trimethoxybenzene (0.163 g, 0.969 mmol, 1.1 equiv.) and Tf_2O (0.273 g, 0.969 mmol, 0.163 mL, 1.1 equiv.) in Et_2O (0.1 M, 9 mL). The title compound **3c** was yielded without further purification as a pink solid (0.162 g, 40%); m.p. 94 – 96 °C; ^1H NMR (500 MHz, CD_2Cl_2) δ 7.98 (s, 1H), 7.87 – 7.81 (m, 2H), 7.50 (d, *J* = 8.3 Hz, 2H), 6.33 (s, 2H), 4.05 (s, 6H), 3.96 (s, 3H), 2.48 (s, 3H); ^{13}C NMR (101 MHz, CD_2Cl_2) δ 169.9, 162.8, 147.7, 131.9, 131.6, 120.2, 92.8, 86.7, 77.7, 57.4, 56.7, 21.5; ^{19}F NMR (376 MHz, CD_2Cl_2) δ -152.96 (s, 1F), -153.01 (s, 3F). IR ν_{max}/cm^{-1}: 2167, 2109, 1592, 1574, 812, 810. HRMS: (APCI) Calculated for $[C_{17}H_{19}O_3Cl_2S\text{-}BF_4]^+$: 373.0418. Found $[M\text{-}BF_4]^+$: 373.0426.

(3d): (dichloromethyl)(4-methoxyphenyl)(2,4,6-trimethoxyphenyl)sulfonium tetrafluoroborate

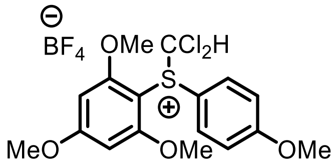

3d

General Procedure C: 1-((dichloromethyl)sulfinyl)-4-methoxybenzene **2d** (0.181 g, 0.750 mmol, 1 equiv.), 1,3,5-trimethoxybenzene (0.139 g, 0.825 mmol, 1.1 equiv.) and Tf_2O (0.233 g, 0.825 mmol, 0.138 mL, 1.1 equiv.) in Et_2O (0.1 M, 8 mL). The title compound **3d** was yielded without further purification as a red solid (0.101 g, 30%); m.p. 101 – 103 °C; ^{1}H NMR (400 MHz, CD_2Cl_2) δ 7.95 – 7.89 (m, 3H), 7.20 – 7.14 (m, 2H), 6.33 (s, 2H), 4.06 (s, 6H), 3.95 (s, 3H), 3.91 (s, 3H); ^{13}C NMR (101 MHz, CD_2Cl_2) δ 170.0, 166.0, 163.0, 134.7, 117.2, 113.4, 93.2, 87.8, 78.4, 57.8, 57.0, 56.6; ^{19}F NMR (376 MHz, CD_2Cl_2) δ -152.86 (s, 1F), -152.91 (s, 3F). IR ν_{max}/cm^{-1}: 2221 2105, 816, 794. HRMS: (APCI) Calculated for $[C_{17}H_{19}O_4Cl_2S\text{-}BF_4]^+$: 389.0376. Found $[M\text{-}BF_4]^+$: 389.0369.

(5a): (2,2-dichloro-1-methylcyclopropyl)benzene

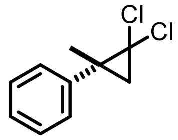

5a

General Procedure D: compound **3a** (18.0 mg, 0.0389 mmol, 1 equiv.), α-methyl styrene (9.18 mg, 0.0777 mmol, 10.10 µL, 2 equiv.) and DIPEA (7.54 mg, 10.2 µL, 0.0584 mmol, 1.5 equiv.) in CH_2Cl_2 (0.04 M, 1 mL). The crude mixture was purified by silica gel chromatography to yield the title compound **5a** as a colourless oil (4.69 mg, 60%); R_f 0.5 (100% *n*-pentane); ^{1}H NMR (500 MHz, $CDCl_3$) δ 7.38 – 7.28 (m, 5H), 1.96 (d, *J* = 7.2 Hz, 1H), 1.67 (s, 3H), 1.59 (d, *J* = 7.2 Hz, 1H); ^{13}C NMR (101 MHz, $CDCl_3$) δ 141.7, 129.0, 128.9, 127.7, 66.4, 36.9, 32.3, 26.0. The data were in accordance with the ones reported in literature.[12]

(5b): 1-(2,2-dichlorocyclopropyl)-4-methoxybenzene

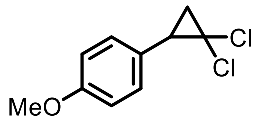

5b

General Procedure E: compound **3a** (50.0 mg, 0.108 mmol, 1 equiv.), 4-vinylanisole (29.0 mg, 0.216 mmol, 28.9 µL, 2 equiv.) and NaH (60% in mineral oil, 16.9 mg, 0.432 mmol, 4 equiv.) in CH_2Cl_2 (0.04 M, 2.5 mL). The crude mixture was purified by preparative TLC to yield the title compound **5b** as a colourless oil (13.8 mg, 59%); R_f 0.4 (9:1 pentane: Et_2O); ^1H NMR (400 MHz, CD_2Cl_2) δ 7.22 – 7.13 (m, 2H), 6.93 – 6.83 (m, 2H), 3.79 (s, 3H), 2.88 (dd, *J* = 10.8, 8.4 Hz, 1H), 1.95 (dd, *J* = 10.7, 7.5 Hz, 1H), 1.82 (dd, *J* = 8.4, 7.5 Hz, 1H); ^{13}C NMR (101 MHz, CD_2Cl_2) δ 162.8, 137.0, 130.6, 114.3, 60.6, 55.9, 35.5, 26.3. The data were in accordance with the ones reported in literature.[12]

(5c): 1-(2,2-dichlorocyclopropyl)-2-methoxybenzene

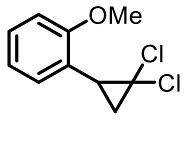

5c

General Procedure E: compound **3a** (11.6 mg, 0.0250 mmol, 1 equiv.), 2-vinylanisole (6.72 mg, 0.0500 mmol, 6.72 µL, 2 equiv.) and NaH (60% in mineral oil, 2.35 mg, 0.100 mmol, 4 equiv.) in CH_2Cl_2 (0.04 M, 0.6 mL). The crude mixture was purified by 10% w/w $AgNO_3$ doped silica gel chromatography to yield the title compound **5c** as a colourless oil (1.62 mg, 30%); R_f 0.2 (100% *n*-pentane); ^1H NMR (500 MHz, CD_2Cl_2) δ 7.34 – 7.26 (m, 1H), 7.02 – 6.89 (m, 3H), 3.92 (s, 3H), 2.96 (dd, *J* = 10.6, 8.7 Hz, 1H), 1.94 (dd, *J* = 10.6, 7.4 Hz, 1H), 1.84 (dd, *J* = 8.7, 7.4 Hz, 1H); ^{13}C NMR (126 MHz, CD_2Cl_2) δ 165.9, 129.3, 128.7, 124.2, 120.5, 110.7, 62.5, 56.1, 31.9, 25.3. HRMS: (APCI) Calculated for $[C_{10}H_{10}Cl_2O+H]^+$: 217.0181. Found $[M+H]^+$: 217.0184.[13] *N.B. quaternary carbons were difficult to see, but presence confirmed by HMBC cross peaks*.

(5d): 1-(2,2-dichlorocyclopropyl)-3-methoxybenzene

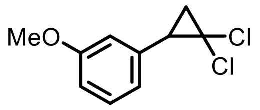

5d

General Procedure E: compound **3a** (12.3 mg, 0.0264 mmol, 1 equiv.), 3-vinylanisole (7.08 mg, 0.0528 mmol, 7.34 µL, 2 equiv.) and NaH (60% in mineral oil, 2.48 mg, 0.106 mmol, 4 equiv.) in CH_2Cl_2 (0.04 M, 0.7 mL) The crude mixture was purified by 10% w/w $AgNO_3$ doped silica gel chromatography to yield the title compound **5d** as a colourless oil (2.44 mg, 43%); R_f 0.2 (100% pentane); ^1H NMR (500 MHz, $CDCl_3$) δ 7.24 – 7.14 (m, 1H), 6.91 – 6.76 (m, 3H), 3.82 (s, 3H), 2.89 (dd, *J* = 10.6, 8.4 Hz, 1H), 1.97 (dd, *J* = 10.6, 7.4 Hz, 1H), 1.85 (dd, *J* = 8.4, 7.4 Hz, 1H); ^{13}C NMR (101 MHz, $CDCl_3$) δ 159.3, 136.0, 129.1, 121.0, 114.6, 112.7, 60.5, 55.1, 35.2, 25.6. HRMS: (APCI) Calculated for $[C_{10}H_{10}Cl_2O+H]^+$: 217.0181. Found $[M+H]^+$: 217.0184.[14]

(5e): 1-chloro-4-(2,2-dichlorocyclopropyl)benzene

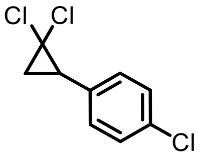

5e

General Procedure E: compound **3a** (50.0 mg, 0.108 mmol, 1 equiv.), *p*-chlorostyrene (29.9 mg, 0.216, 25.9 µL mmol, 2 equiv.) and NaH (60% in mineral oil, 16.9 mg, 0.432 mmol, 4 equiv.) in CH_2Cl_2 (0.04 M, 2.5 mL). The crude mixture was purified by preparative TLC to yield the title compound **5e** a colourless oil (9.57 mg, 40%); R_f 0.5 (100% pentane); ^1H NMR (400 MHz, CD_2Cl_2) δ 7.37 – 7.31 (m, 2H), 7.23 – 7.18 (m, 2H), 2.91 (dd, *J* = 10.7, 8.4 Hz, 1H), 2.01 (dd, *J* = 10.7, 7.6 Hz, 1H), 1.86 (dd, *J* = 8.4, 7.6 Hz, 1H); ^{13}C NMR (101 MHz, CD_2Cl_2) δ 133.8, 133.7, 130.7, 128.8, 61.1, 35.2, 26.2. The data were in accordance with the ones reported in literature.[12]

(5f): (2,2-dichlorocyclopropyl)benzene

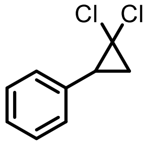

5f

General Procedure E: compound **3a** (50.0 mg, 0.108 mmol, 1 equiv.), styrene (22.5 mg, 0.216 mmol, 24.7 µL, 2 equiv.) and NaH (60% in mineral oil, 16.9 mg, 0.432 mmol, 4 equiv.) in CH_2Cl_2 (0.04 M, 2.5 mL). The crude mixture was purified by preparative TLC to yield the title compound **5f** as a colourless oil (12.7 mg, 63%); R_f 0.6 (100% pentane); ^{1}H NMR (500 MHz, CD_2Cl_2) δ 7.40 – 7.33 (m, 2H), 7.33 – 7.28 (m, 1H), 7.28 – 7.23 (m, 2H), 2.94 (dd, *J* = 10.7, 8.5 Hz, 1H), 1.99 (dd, *J* = 10.7, 7.5 Hz, 1H), 1.89 (dd, *J* = 8.4, 7.5 Hz, 1H); ^{13}C NMR (126 MHz, CD_2Cl_2) δ 135.1, 129.2, 128.7, 127.9, 61.4, 35.8, 26.0. The data were in accordance with the ones reported in literature.[12]

(5g): 2-(2,2-dichlorocyclopropyl)naphthalene

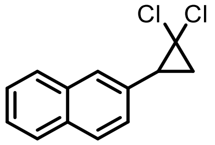

5g

General Procedure E: compound **3a** (20.0 mg, 0.0430 mmol, 1 equiv.), vinyl-naphthalene (13.3 mg, 0.0860 mmol, 2 equiv.) and NaH (60% in mineral oil, 4.03 mg, 0.172 mmol, 4 equiv.) in CH_2Cl_2 (0.04 M, 1 mL). The crude mixture was purified by silica gel chromatography doped with 10% w/w $AgNO_3$ to yield the title compound **5g** as colourless oil (3.16 mg, 31%); R_f 0.3 (100% pentane); ^{1}H NMR (400 MHz, CD_2Cl_2) δ 7.84 (t, *J* = 8.0 Hz, 3H), 7.68 (d, *J* = 1.7 Hz, 1H), 7.51 – 7.41 (m, 3H), 3.10 (dd, *J* = 10.4, 8.6 Hz, 1H), 2.10 – 2.01 (m, 2H); ^{13}C NMR (126 MHz, CD_2Cl_2) δ 133.6, 133.1, 132.8, 128.2, 128.1, 128.0, 127.9, 127.4, 126.7, 126.5, 61.5, 36.1, 26.1. HRMS: (APCI) Calculated for $[C_{13}H_{10}Cl_2+H]^+$: 237.0232. Found $[M+H]^+$: 237.0232.

(5h): 4-(2,2-dichlorocyclopropyl)phenyl acetate

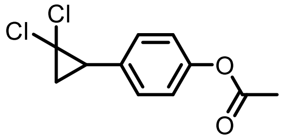

5h

General Procedure E: compound **3a** (50.0 mg, 0.108 mmol, 1 equiv.), *p*-acetoxystyrene (35 mg, 0.216 mmol, 33 µL, 2 equiv.) and NaH (60% in mineral oil, 16.9 mg, 0.432 mmol, 4 equiv.) in CH_2Cl_2 (0.04 M, 2.5 mL). The crude mixture was purified by preparative TLC to yield **5h** as a yellow oil (18mg, 68%); R_f 0.4 (5:1 pentane:Et_2O); ^{1}H NMR (500 MHz, CD_2Cl_2) δ 7.31 – 7.24 (m, 2H), 7.11 – 7.05 (m, 2H), 2.93 (dd, *J* = 10.7, 8.3 Hz, 1H), 2.27 (s, 3H), 2.01 (dd, *J* = 10.7, 7.6 Hz, 1H), 1.86 (dd, *J* = 8.4, 7.6 Hz, 1H); ^{13}C NMR (126 MHz, CD_2Cl_2) δ 169.7, 150.6, 132.7, 130.3, 121.9, 61.2, 35.3, 26.3, 21.2. HRMS: (APCI) Calculated for $[C_{11}H_{10}Cl_2O_2+H]^+$: 245.0131. Found $[M+H]^+$: 245.0129.

(5i): *trans*-1,1-dichlorospiro[2.3]hexane-5-carbonitrile

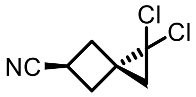

5i

General Procedure E: compound **3a** (14.7 mg, 0.0315 mmol, 1 equiv.), 3-methylenecyclobutene-1-carbonitrile (5.87 mg, 0.0630 mmol, 6.43 µL, 2 equiv.) and NaH (60% in mineral oil, 2.95 mg, 0.126 mmol, 4 equiv.) in CH_2Cl_2 (0.04 M, 0.8 mL). The title compound **5i** was yielded as a mixture of diastereomers (1:1.38 *dr*). The crude mixture was purified by silica gel chromatography to yield **5i** as a white solid (*trans* CN and CCl_2 fragment) (1.40 mg, 25%). Slow evaporation of CH_2Cl_2 gave single crystals suitable for single crystal XRD; R_f 0.4 (9:1 pentane:EtOAc); m.p. 90 – 93 °C; ^{1}H NMR (500 MHz, $CDCl_3$) δ 3.20 (tt, *J* = 9.5, 6.8 Hz, 1H), 2.82 – 2.72 (m, 2H), 2.54 – 2.44 (m, 2H), 1.44 (s, 2H); ^{13}C NMR (101 MHz, $CDCl_3$) δ 122.1, 62.8, 31.8, 31.5, 31.3, 16.8. HRMS: (ESI) Calculated for $[C_7H_7Cl_2N+H]^+$: 176.0028. Found $[M+H]^+$: 176.0024.

(5j): *tert*-butyl 1,1-dichloro-6-azaspiro[2.5]octane-6-carboxylate

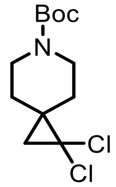

5j

General Procedure D: compound **3a** (30.5 mg, 0.0655 mmol, 1 equiv.), *tert*-butyl 4-methylenepiperidine-1-carboxylate (25.8 mg, 0.131 mmol, 25.9 µL, 2 equiv.) and DIPEA (12.7 mg, 17.1 µL, 0.0983 mmol, 1.5 equiv.) in CH_2Cl_2 (0.04 M, 1.6 mL). The crude mixture was purified by a silica gel chromatography doped with 10% w/w $AgNO_3$ to yield the title compound **5j** as a white solid (3.68 mg, 20%); R_f 0.4 (5:2 pentane: Et_2O); m.p. 86 – 88 °C; ^{1}H NMR (700 MHz, CD_2Cl_2) δ 3.57 (ddd, *J* = 13.0, 7.2, 3.9 Hz, 2H), 3.39 (ddd, *J* = 13.2, 7.9, 3.6 Hz, 2H), 1.79 (ddd, *J* = 13.7, 7.9, 3.7 Hz, 2H), 1.63 (ddd, *J* = 13.6, 7.2, 3.6 Hz, 2H), 1.45 (s, 9H), 1.31 (s, 2H); ^{13}C NMR (176 MHz, CD_2Cl_2) δ 155.0, 79.7, 67.1, 43.5, 43.0, 32.9, 32.0, 31.5, 28.5. HRMS: (APCI) Calculated for $[C_{12}H_{19}Cl_2O_2N+H]^+$: 280.0854. Found $[M+H]^+$: 280.0866.[15]

(5k): (*trans*-2,2-dichloro-3-methylcyclopropyl)benzene

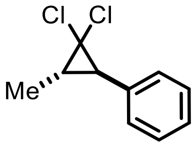

5k

General Procedure E: compound **3a** (19.3 mg, 0.0416 mmol, 1 equiv.), *trans*-β-methyl styrene (9.83 mg, 0.0832 mmol, 10.8 µL, 2 equiv.) and NaH (60% in mineral oil, 3.90 mg, 0.166 mmol, 4 equiv.) in CH_2Cl_2 (0.04 M, 1 mL). The crude mixture was purified by silica gel chromatography to yield the title compound **5k** as a colourless oil (3.34 mg, 40%); R_f 0.5 (100% *n*-pentane); ^{1}H NMR (400 MHz, $CDCl_3$) δ 7.41 – 7.31 (m, 3H), 7.27 – 7.23 (m, 2H), 2.43 (d, *J* = 8.4 Hz, 1H), 1.99 (dq, *J* = 8.3, 6.2 Hz, 1H), 1.49 (d, *J* = 6.1 Hz, 3H); ^{13}C NMR (101 MHz, $CDCl_3$) δ 135.2, 128.8, 128.4, 127.6, 66.7, 41.8, 29.9, 15.0. GCMS: (EI) Calculated for $[C_{10}H_{10}Cl_2]^{+\bullet}$: 200.09. Found $[M]^{+\bullet}$: 200.00. The data were in accordance with the ones reported in literature.[16]

(5l): (*cis*-2,2-dichloro-3-methylcyclopropyl)benzene

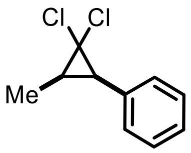

5l

General Procedure E: compound **3a** (20.6 mg, 0.0442 mmol, 1 equiv.), *cis*-β-methyl styrene (10.6 mg, 0.0884 mmol, 11.4 µL) and NaH (60% in mineral oil, 4.15 mg, 0.177 mmol, 4 equiv.) in CH_2Cl_2 (0.04 M, 1 mL). The crude mixture was purified by silica gel chromatography to yield the title compound **5l** a colourless oil (3.64 mg, 41%); R_f 0.6 (100% pentane); ^{1}H NMR (400 MHz, CD_2Cl_2) δ 7.38 – 7.32 (m, 2H), 7.29 (d, *J* = 7.2 Hz, 3H), 2.88 (d, *J* = 11.2 Hz, 1H), 2.09 (dq, *J* = 13.2, 6.6 Hz, 1H), 1.16 (dd, *J* = 6.6, 1.1 Hz, 3H); ^{13}C NMR (126 MHz, CD_2Cl_2) δ 133.5, 130.9, 128.6, 127.5, 65.9, 36.4, 29.9, 11.4. GCMS: (EI) Calculated for $[C_{10}H_{10}Cl_2]^{+\bullet}$: 200.09. Found $[M]^{+\bullet}$: 200.05. The data were in accordance with the ones reported in literature.[16]

(5m): 7,7-dichloro-1-methylbicyclo[4.1.0]heptane

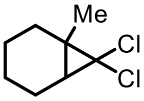

5m

General Procedure D: compound **3a** (25.0 mg, 0.0530 mmol, 1 equiv.), 1-methylcyclohex-1-ene (10.2 mg, 0.106 mmol, 12.8 µL, 2 equiv.) and DIPEA (10.3 mg, 13.8 µL, 0.0795 mmol, 1.5 equiv.) in CH_2Cl_2 (0.04 M, 1.3 mL). The crude mixture was purified by silica gel chromatography to yield the title compound **5m** as a colourless oil (2.53 mg, 34%); R_f 0.3 (100% pentane); ^{1}H NMR (400 MHz, $CDCl_3$) δ 1.98 – 1.76 (m, 2H), 1.69 (dtd, *J* = 12.7, 6.3, 2.3 Hz, 2H), 1.43 – 1.27 (m, 8H). *N.B.* the title compound was extremely volatile so a ^{13}C spectrum was not able to be obtained and chromatography solvent was difficult to remove without compound loss. The data were in accordance with the ones reported in literature.[17]

(5n): ((7,7-dichlorobicyclo[4.1.0]heptan-1-yl)oxy)trimethylsilane

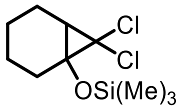

5n

General Procedure D: compound **3a** (50.0 mg, 0.108 mmol, 1 equiv.), (cyclohex-1-en-1-yloxy)trimethylsilane (36.6 mg, 0.216 mmol, 41.9 µL, 2 equiv.) and DIPEA (20.9 mg, 28 µL, 0.162 mmol, 1.5 equiv.) in CH_2Cl_2 (0.04 M, 2.5 mL). The crude mixture was purified by a silica gel chromatography to yield the title compound **5n** as a colourless oil (19.1 mg, 70%); R_f 0.2 (100% pentane); ^{1}H NMR (400 MHz, CD_2Cl_2) δ 2.20 (dt, *J* = 14.6, 6.0 Hz, 1H), 2.12 – 1.94 (m, 3H), 1.71 – 1.56 (m, 2H), 1.50 – 1.19 (m, 3H), 0.21 (s, 9H); ^{13}C NMR (101 MHz, CD_2Cl_2) δ 66.3, 61.5, 34.7, 29.0, 21.6, 20.5, 19.8, 1.2. HRMS: (ESI) Calculated for $[C_{10}H_{18}Cl_2OSi+H]^+$: 253.0577. Found $[M+H]^+$: 253.0574.[18]

References

1. Williams, C. M., Mander, L. N. Chromatography with silver nitrate. *Tetrahedron*, **2001**, *57*, 425-447.

2. Kohn, W., Sham, L. J. Self-Consistent Equations Including Exchange and Correlation Effects. *Phys. Rev*., **1965**, *140*, A1133–A1138.

3. Hohenberg, P., Kohn, W. Inhomogeneous Electron Gas. *Phys. Rev.,* **1964**, B864–B871.

4. Peng, C., Ayala, P. Y., Schlegel, H. B. Frisch, M. J. Using redundant internal coordinates to optimize equilibrium geometries and transition states. J. Comp. Chem., **1996**, *1*, 49–56.

5. Rigaku Oxford Diffraction, CrysAlisPro Software system, Rigaku Corporation, Oxford, UK, **2020**.

6. Sheldrick, G. M. SHELXT – Integrated space-group and crystal structure determination. *Acta Cryst*., **2015**, *A71,* 3-8.; Sheldrick, G. M. Crystal structure refinement with SHELXL. *Acta Cryst*., **2015**, *C71*, 3-8.

7. Dolomanov, O. V., Bourhis, L. J., Gildea, R. J., Howard, J. A. K., Puschmann, H. OLEX2: a complete structure solution, refinement and analysis program. *J. Appl. Cryst.,* **2009**, 339–341.

8. Noguchi, T., Miyagawa, T., Satoh, T. Resolution of racemic aryl dichloromethyl sulfoxides with (−)-menthone. *Tetrahedron: Asymmetry*, **2009**, *20*, 2073–2076.

9. Lu, S., Li, X., Qin, W., Liu, J., Huang, Y., Wong, H., Liu, G. Air- and Light-Stable *S*-(Difluoromethyl)sulfonium Salts: *C*-SelectiveElectrophilic Difluoromethylation of β-Ketoesters and Malonates. Org. Lett., 2018, 20, 6925–6929.

10. Wu, C., Li, X., Li, T., Shao, M., Niu, L., Lu, X., Kan, J., Geng, Y.**,** Dong, Y. Photochemical Synthesis of Covalent Organic Frameworks. *J. Am. Chem. Soc.,* **2022**, *144*, 18750–18755.

11. Gahalawat, S., Addepalli, Y., Fink S. P., Kasturi, L., Markowitz, S. D., Ready, J. M. Enzymatic Resolution and Decarboxylative Functionalization of α-Sulfinyl Esters. *Chem. Eur. J.* 2024, *7*, e202302996.

12. Lee, J. W., Jang, Y. S., Park, J. M, Park, C. P. Synthesis of *gem*-Dichlorocyclopropanes Using Liquid–Liquid Slug Flow. *Bull. Korean Chem. Soc.*, **2021**, *42*, 1089–1092.

13. Phillips, D.K. US3948973A, **1976**; Halocyclopropyl substituted phenoxyalkanoic acids.

14. Kostikov, R.R. *Russ. J. Org. Chem.*, **1977**, *9*, 1712–1721.

15. Kudou, T., Tanima, D., Masuzawa, Y, Yano, T. EP2336104A1, **2011**; Ortho-substituted haloalkylsulfonanilide derivative and herbicide.

16. Kusuyama, K., Kubo, T., Iyo, M., Kagosaku, T., Tokami, K. Substituent Effects in the Solvolysis of p-(2-Substituted Cyclopropyl)-α-Methylbenzyl Chlorides. *Bull. Chem. Soc. Jpn.,* **1991**, *10*, 2954–2960.

17. Tanner, D. D., Zhang, L., Hu, L. Q., Kandanarachchi, P. Formation of Three-Membered Rings by SHi Displacement. Reverse of Cyclopropyl Ring Opening. *J. Org. Chem*., **1996,** *20*, 6818–6824.

18. Amice, P., Blanco, L., Conia, J. M. Enol Silyl Ethers and their Use for the Synthesis of α-Halo-α,β-unsaturated Carbonyl Compounds. *Synthesis*, **1976**, *3***,** 196–197.

NMR Spectra of Compounds

(1a): 1-fluoro-4-(methylsulfinyl)benzene

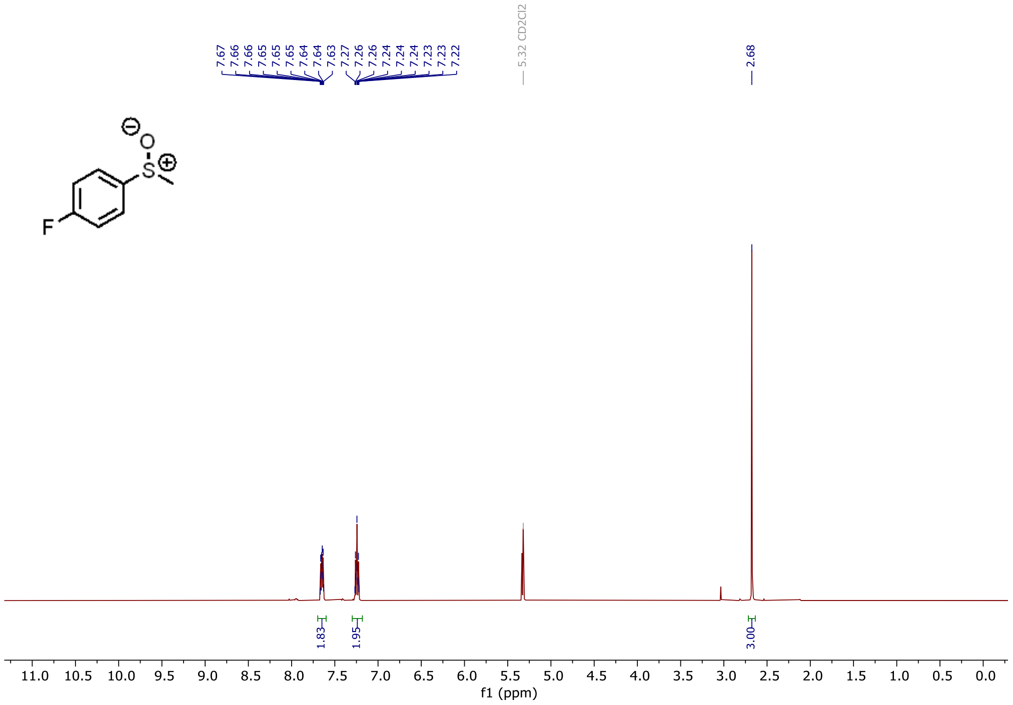

Figure S1: The ^{1}H NMR (500 MHz, CD_2Cl_2) spectra for compound **1a.**

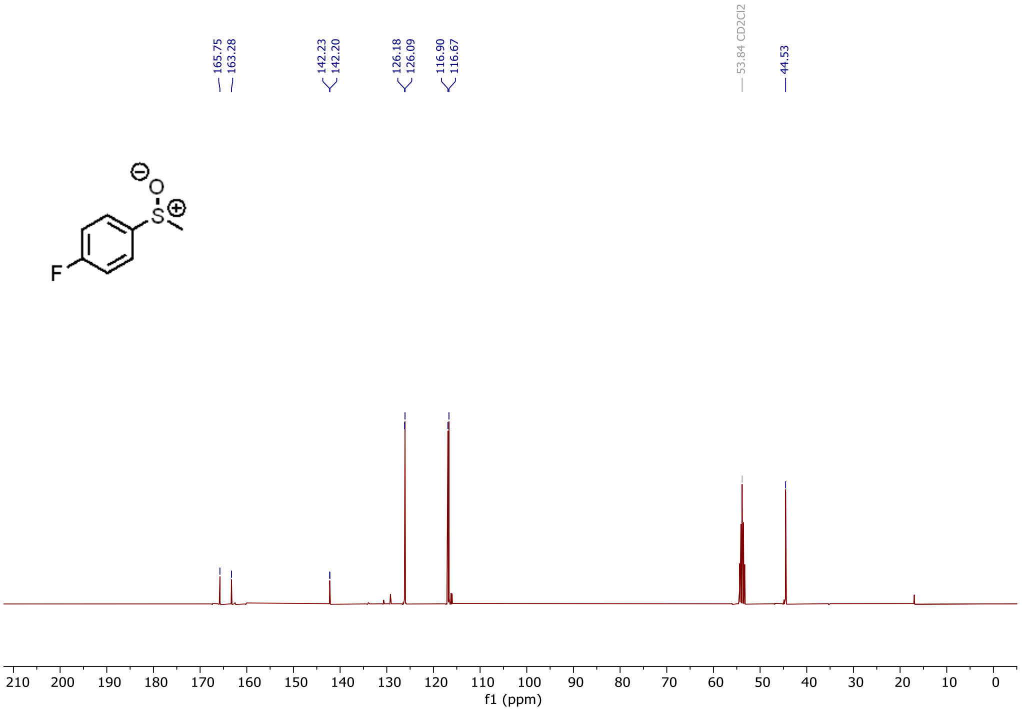

Figure S2: The ^{13}C NMR (101 MHz, CD_2Cl_2) spectra for compound **1a.**

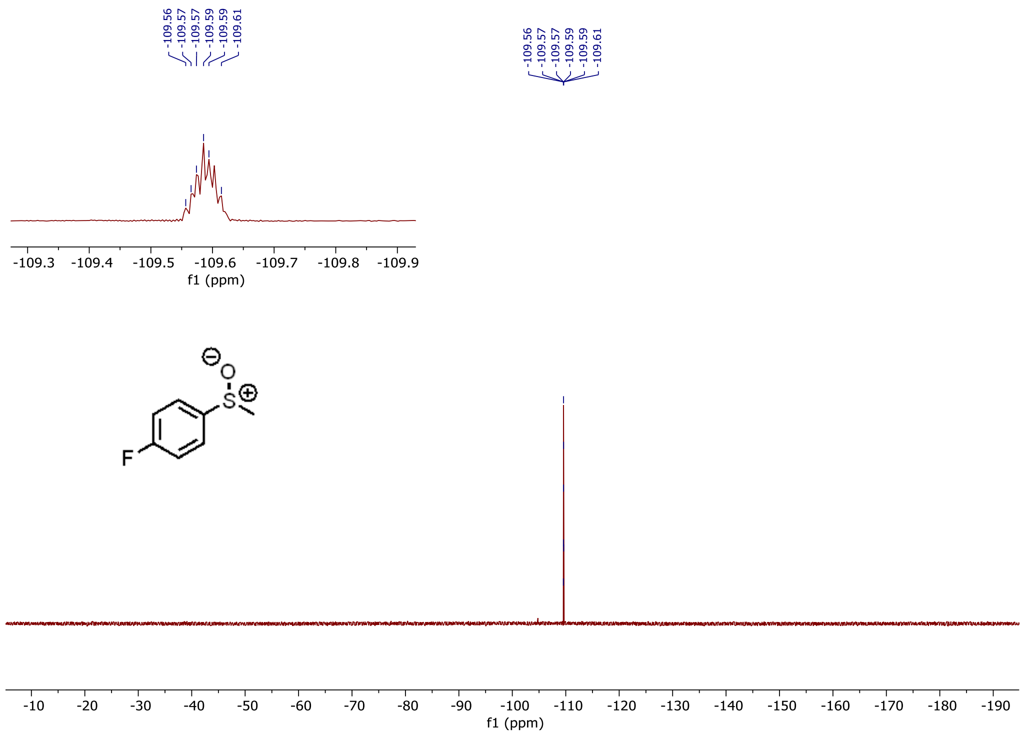

Figure S3: The ^{19}F NMR (471 MHz, CD_2Cl_2) spectra for compound **1a**.

(1d): 1-methoxy-4-(methylsulfinyl)benzene

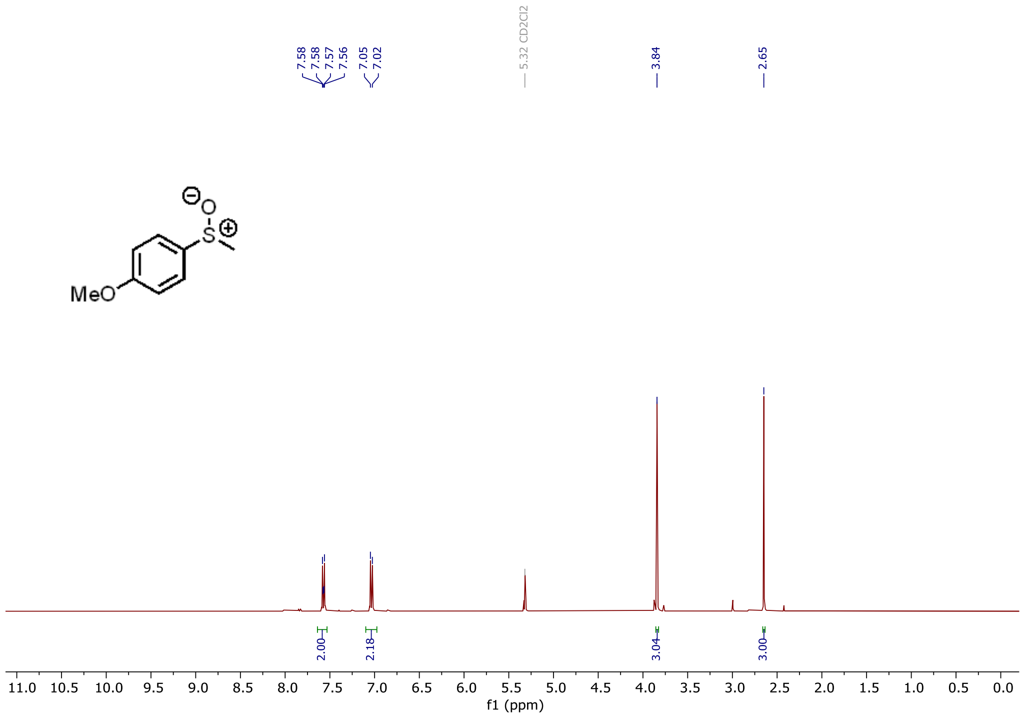

Figure S4: The ^{1}H NMR (400 MHz, CD_2Cl_2) spectra for compound **1d.**

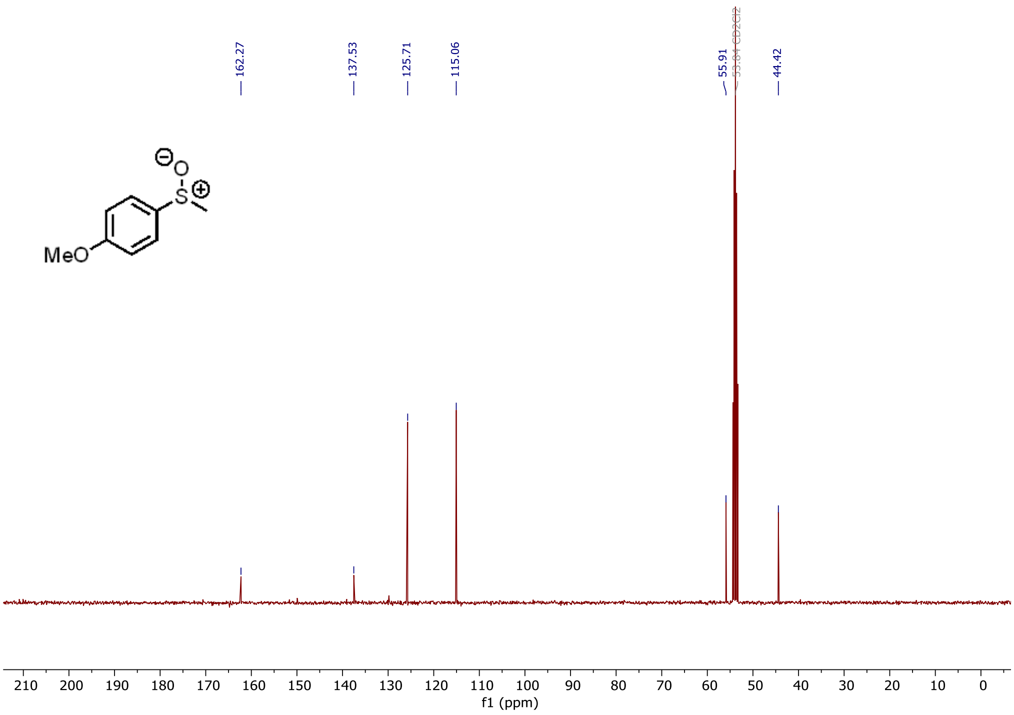

Figure S5: The ^{13}C NMR (101 MHz, CD_2Cl_2) spectra for compound **1d**.

(2a): 1-((dichloromethyl)sulfinyl)-4-fluorobenzene

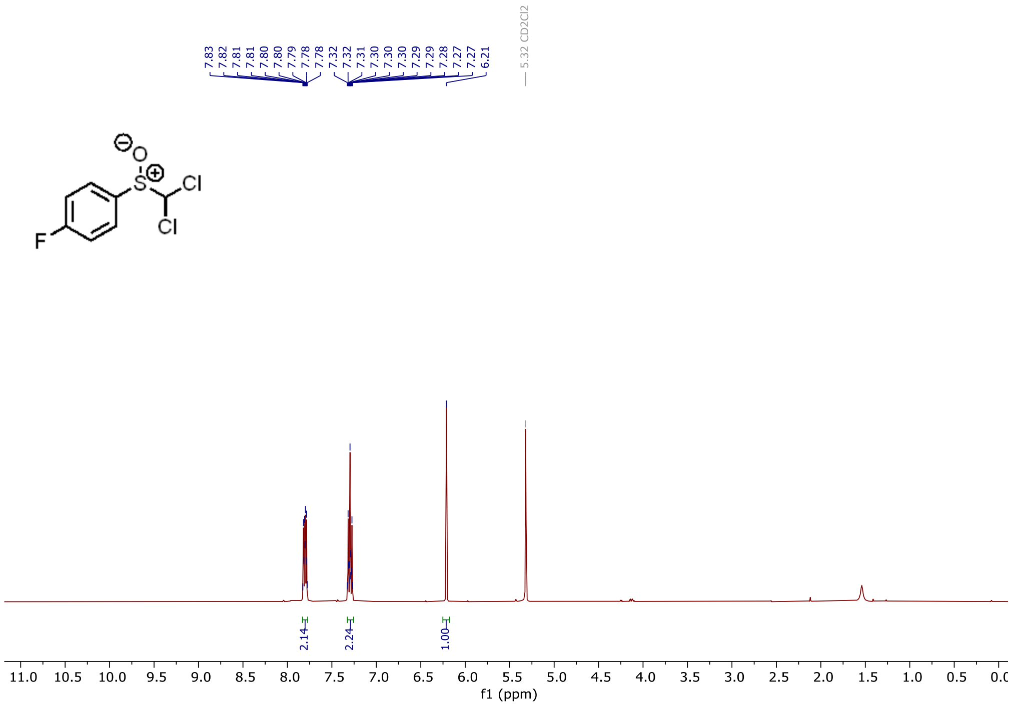

Figure S6: The ^{1}H NMR (500 MHz, CD_2Cl_2) spectra for compound **2a.**

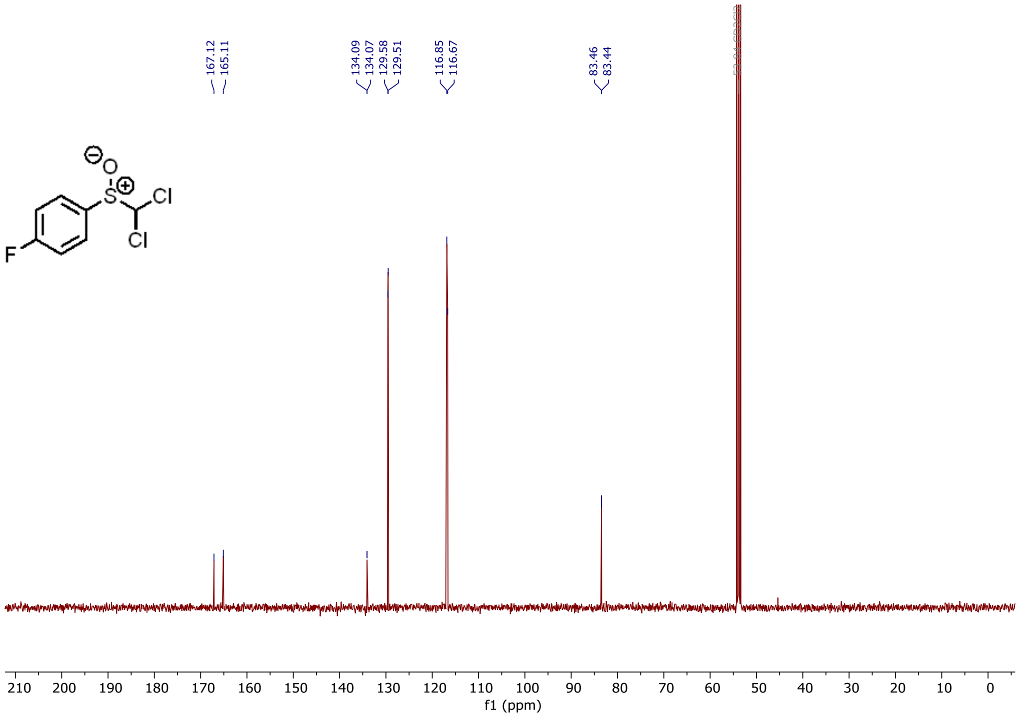

Figure S7: The ^{13}C NMR (126 MHz, CD_2Cl_2) spectra for compound **2a**.

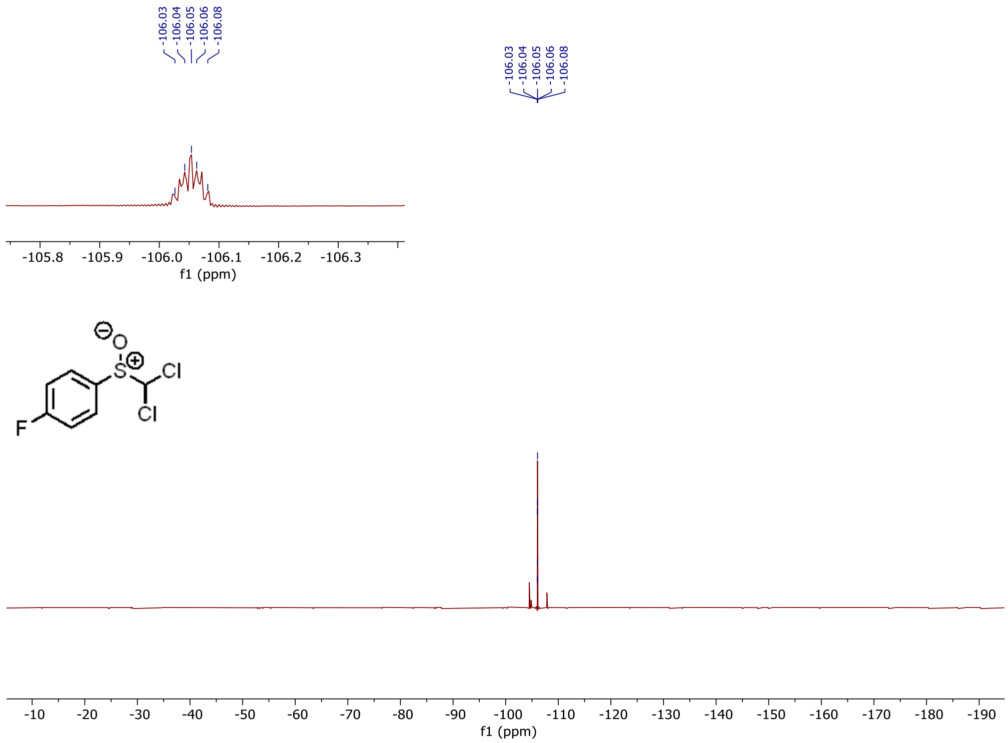

Figure S8: The ^{19}F NMR (471 MHz, CD_2Cl_2) spectra for compound **2a**.

(2b): ((dichloromethyl)sulfinyl)benzene

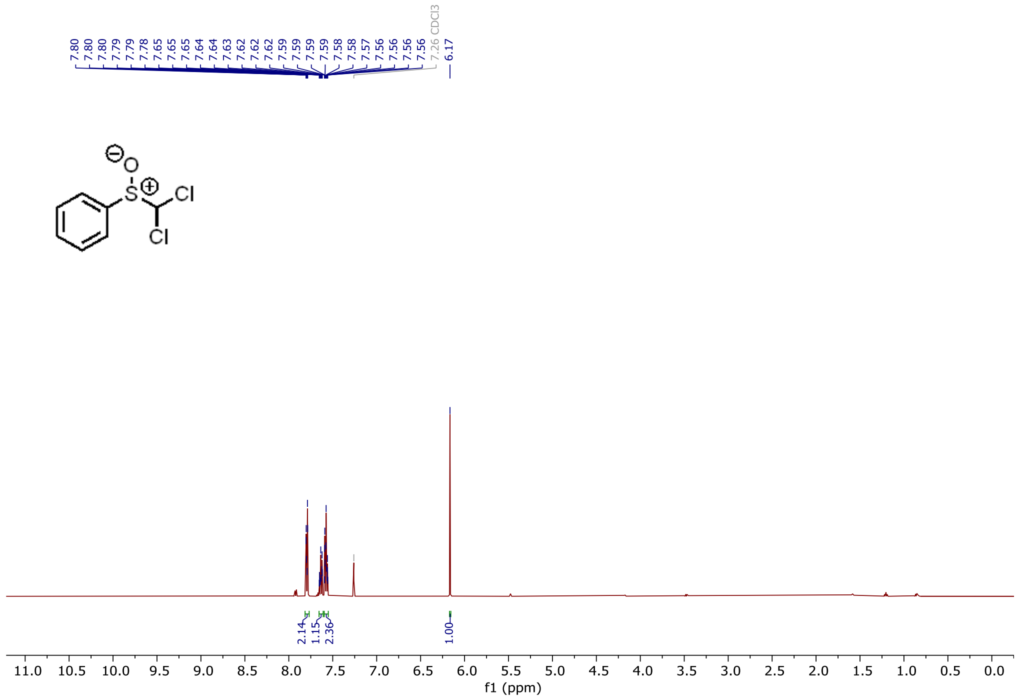

Figure S9: The ^{1}H NMR (500 MHz, $CDCl_3$) spectra for compound **2b**.

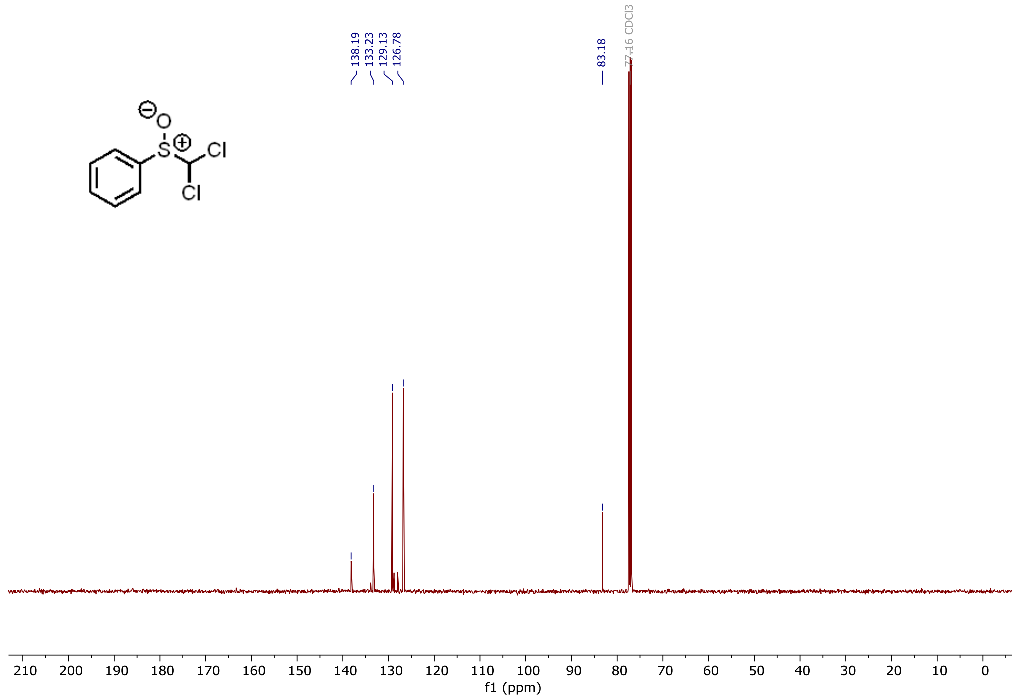

Figure S10: The ^{13}C NMR (126 MHz, $CDCl_3$) spectra for compound **2b**.

(2c): 1-((dichloromethyl)sulfinyl)-4-methylbenzene

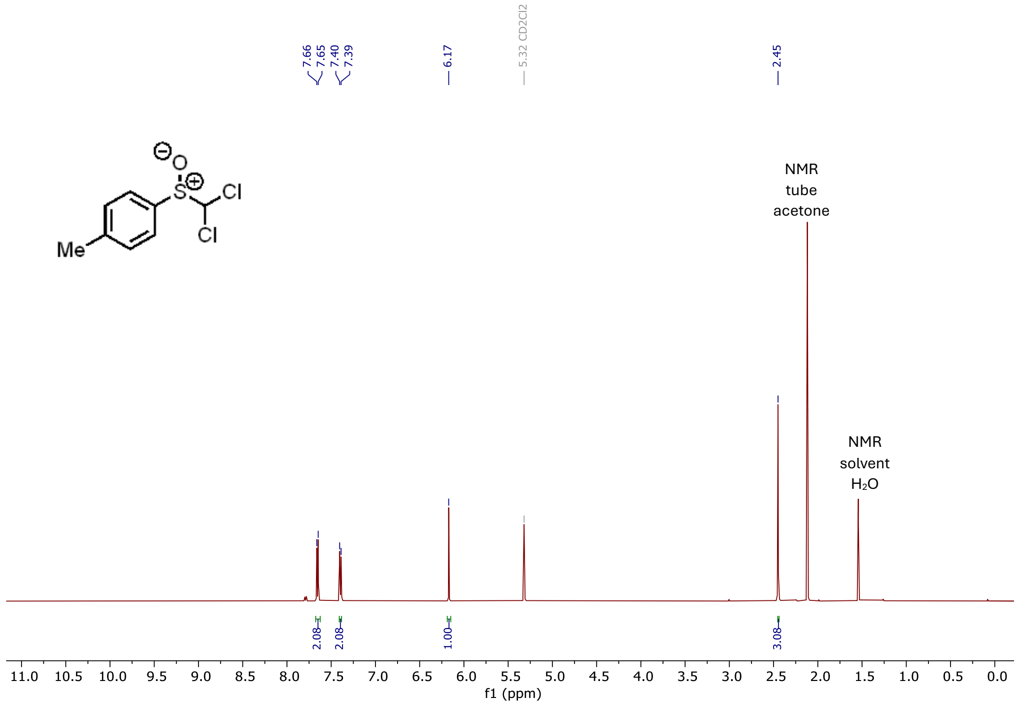

Figure S11: The ^{1}H NMR (500 MHz, CD_2Cl_2) spectra for compound **2c**.

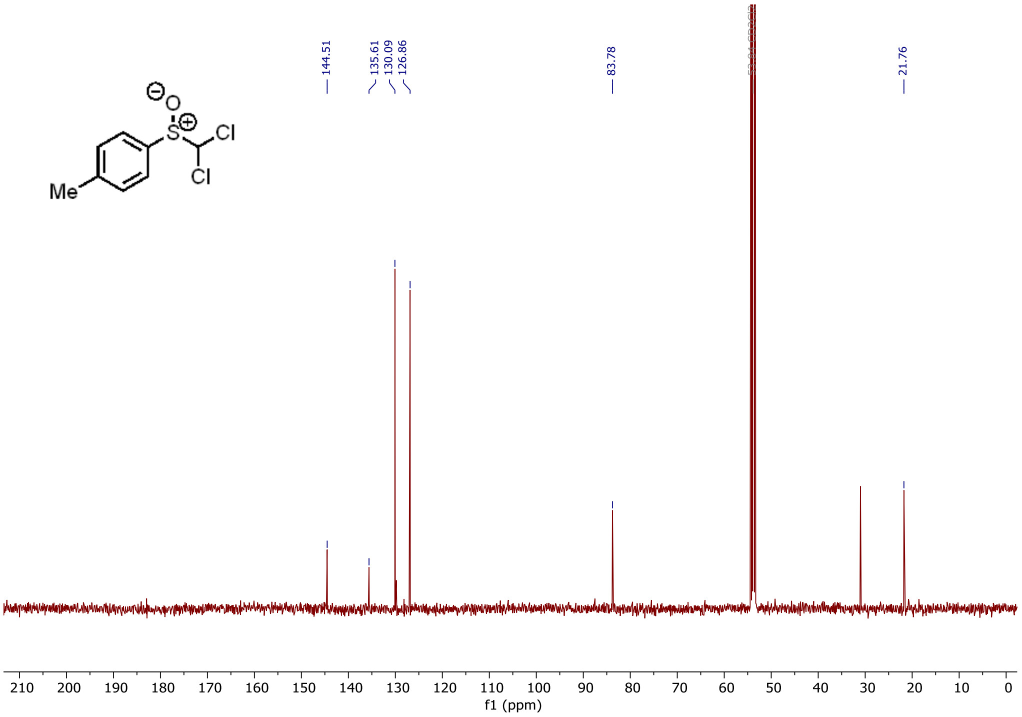

Figure S12: The ^{13}C NMR (126 MHz, CD_2Cl_2) spectra for compound **2c**.

(2d): 1-((dichloromethyl)sulfinyl)-4-methoxybenzene

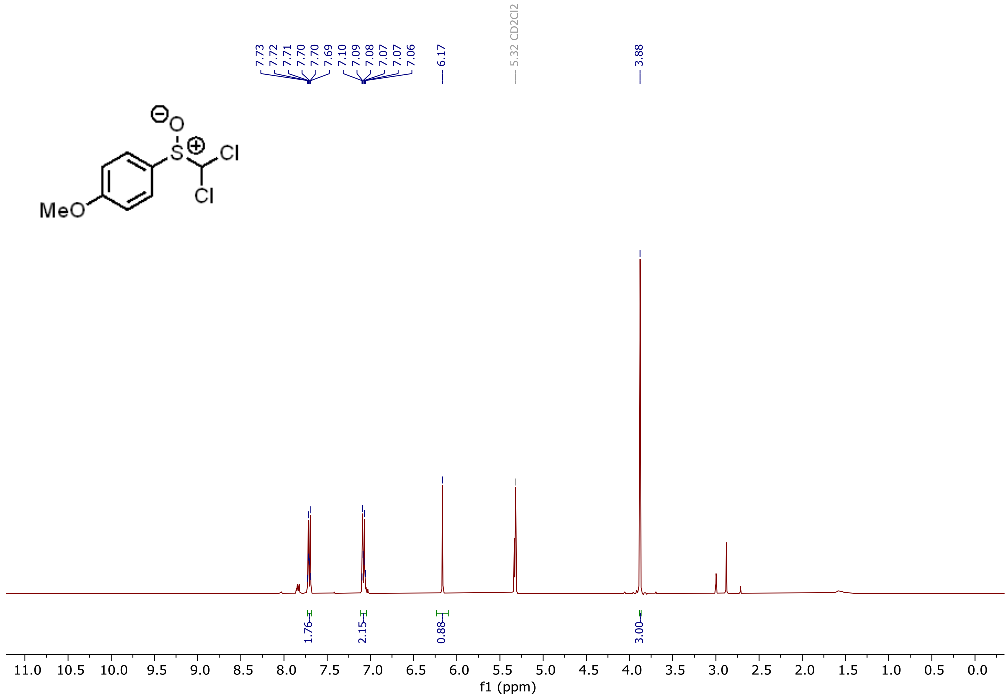

Figure S13: The ^{1}H NMR (400 MHz, CD_2Cl_2) spectra for compound **2d**.

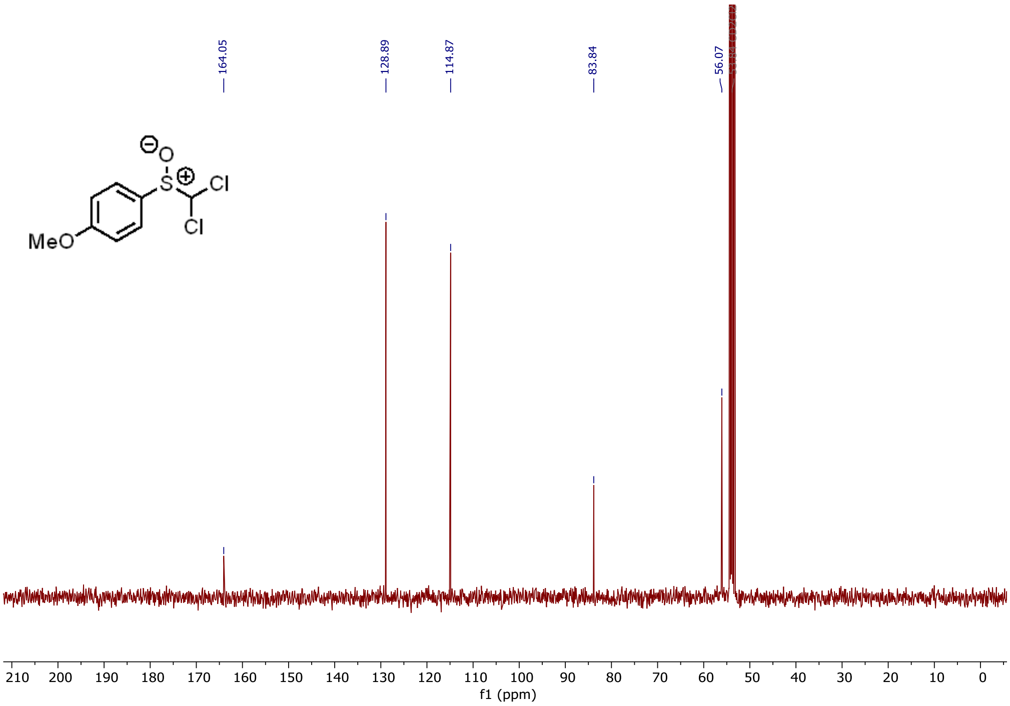

Figure S14: The ^{13}C NMR (101 MHz, CD_2Cl_2) spectra for compound **2d**.

(3a): (dichloromethyl)(4-fluorophenyl)(2,4,6-trimethoxyphenyl)sulfonium tetrafluoroborate

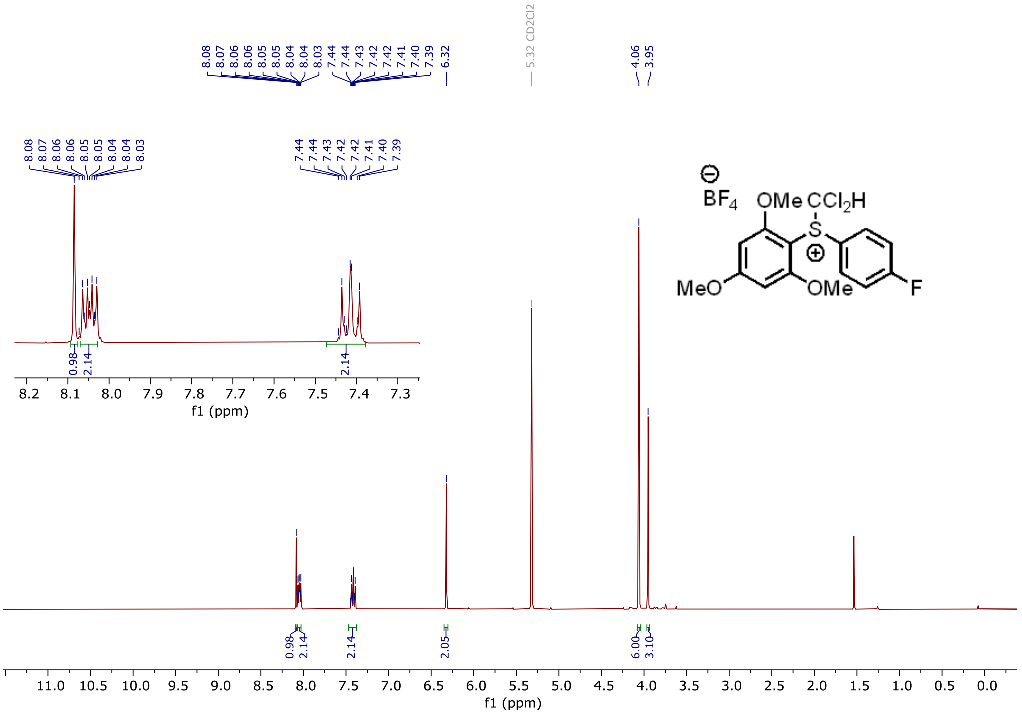

Figure S15: The ^{1}H NMR (400 MHz, CD_2Cl_2) spectra for compound **3a**.

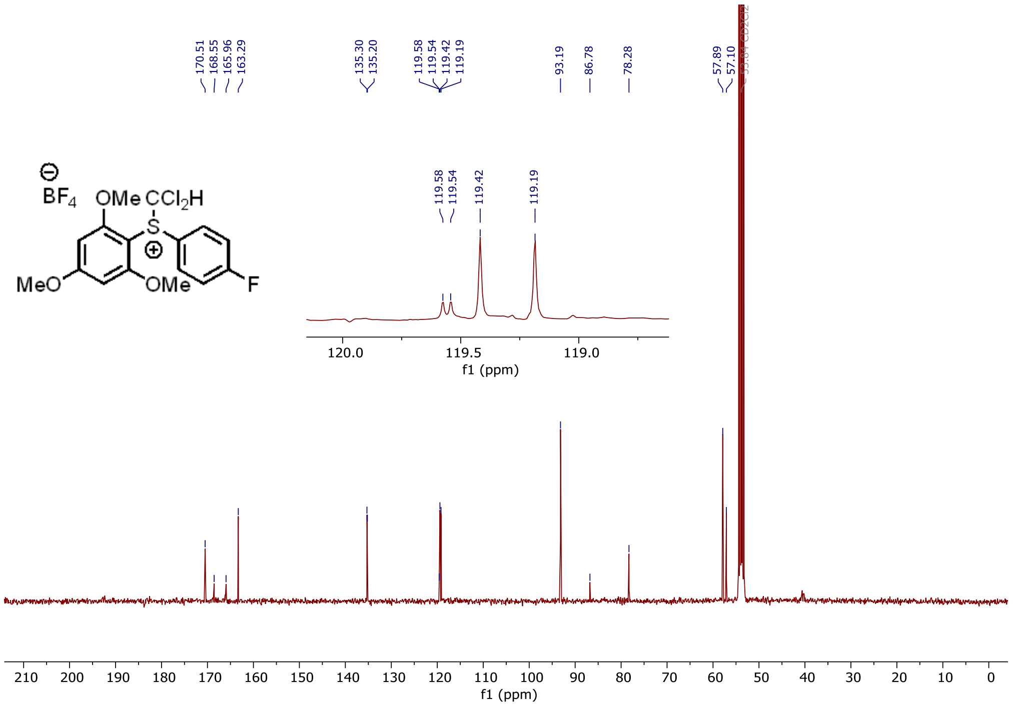

Figure S16: The ^{13}C NMR (101 MHz, CD_2Cl_2) spectra for compound **3a**.

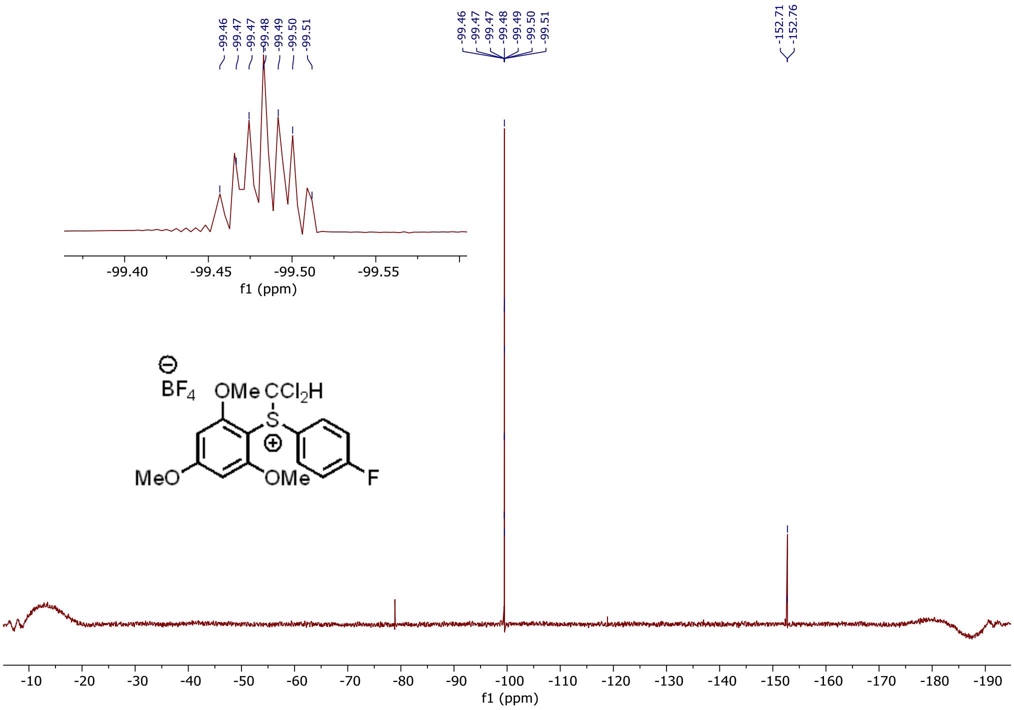

Figure S17: The ^{19}F NMR (376 MHz, CD_2Cl_2) spectra for compound **3a**.

(3b): (dichloromethyl)(phenyl)(2,4,6-trimethoxyphenyl)sulfonium tetrafluoroborate

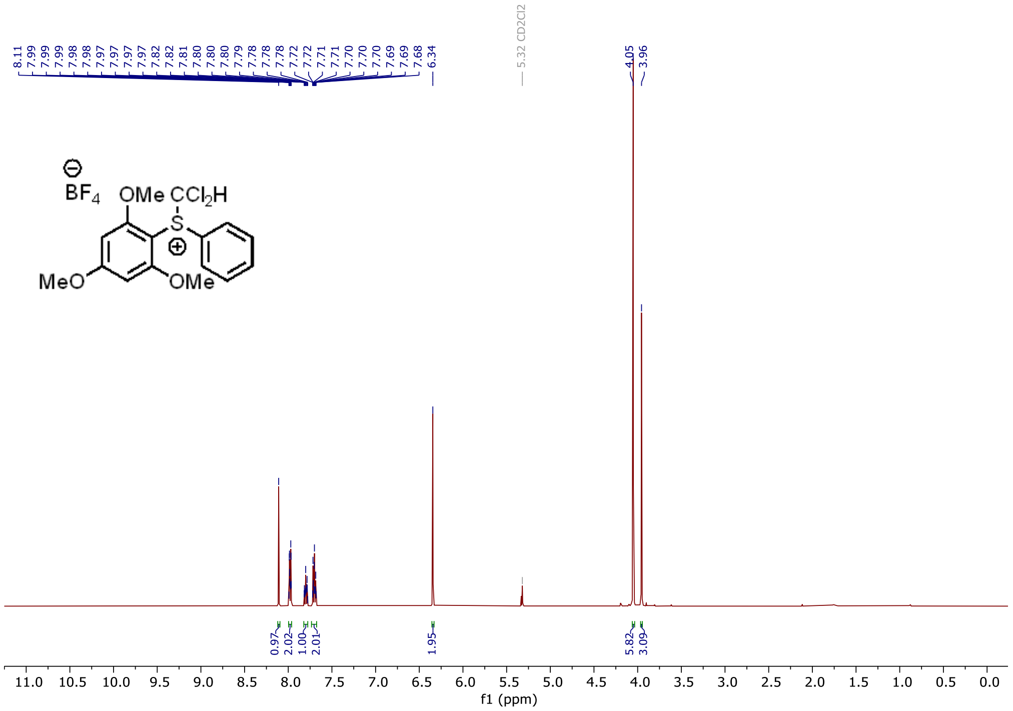

Figure S18: The ^{1}H NMR (500 MHz, CD_2Cl_2) spectra for compound **3b**.

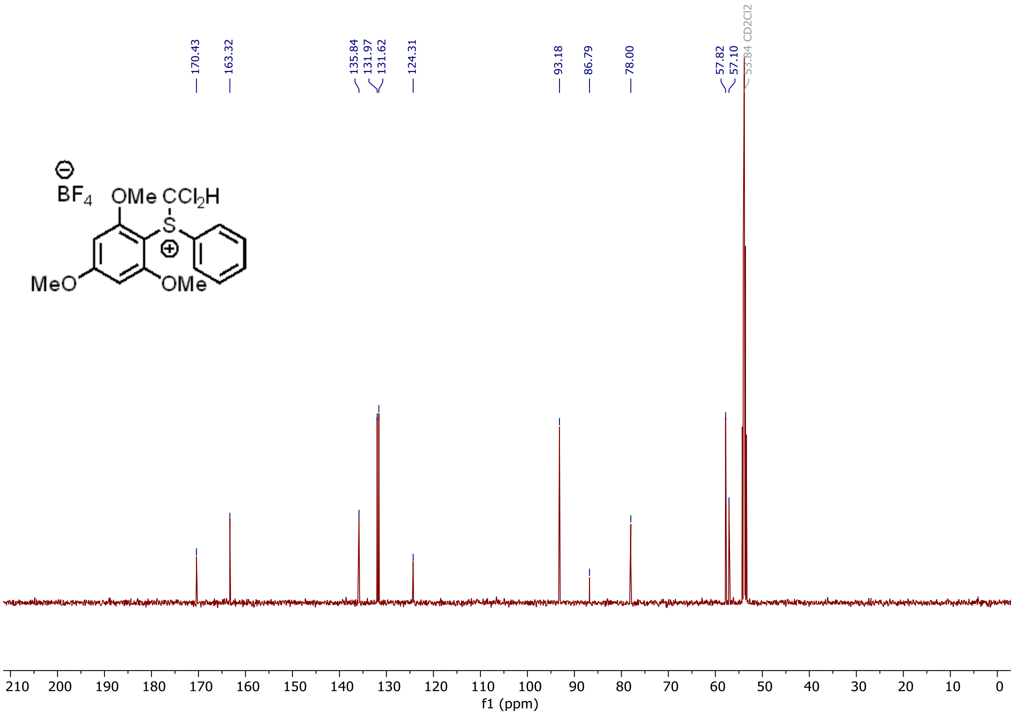

Figure S19: The ^{13}C NMR (126 MHz, CD_2Cl_2) spectra for compound **3b**.

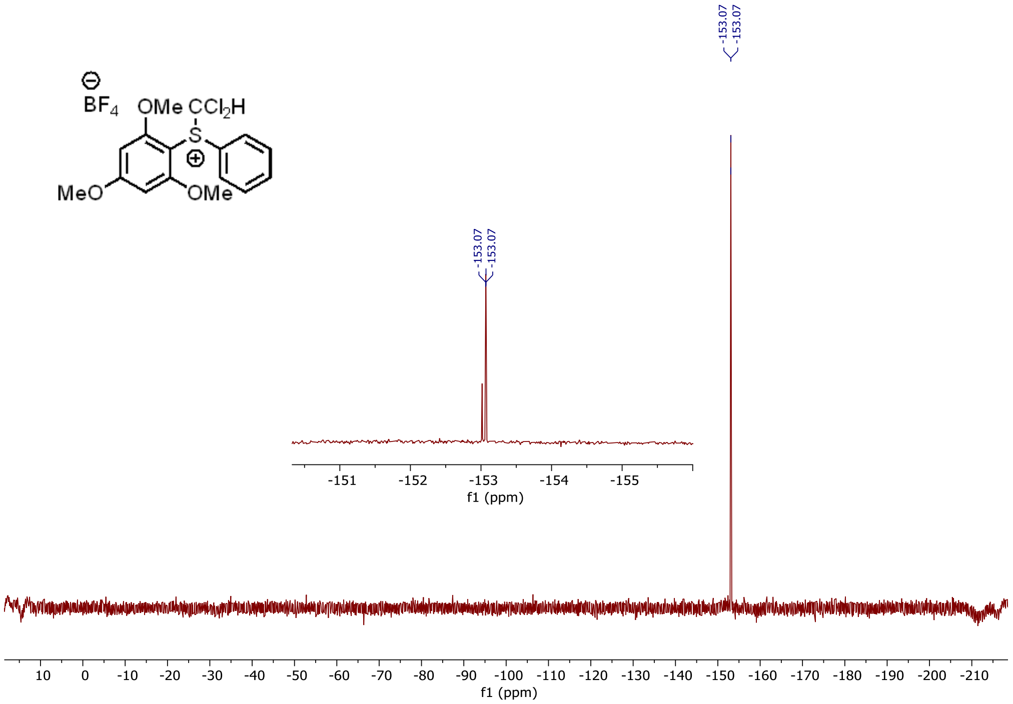

Figure S20: The ^{19}F NMR (376 MHz, CD_2Cl_2) spectra for compound **3b**.

(3c): (dichloromethyl)(*p*-tolyl)(2,4,6-trimethoxyphenyl)sulfonium tetrafluoroborate

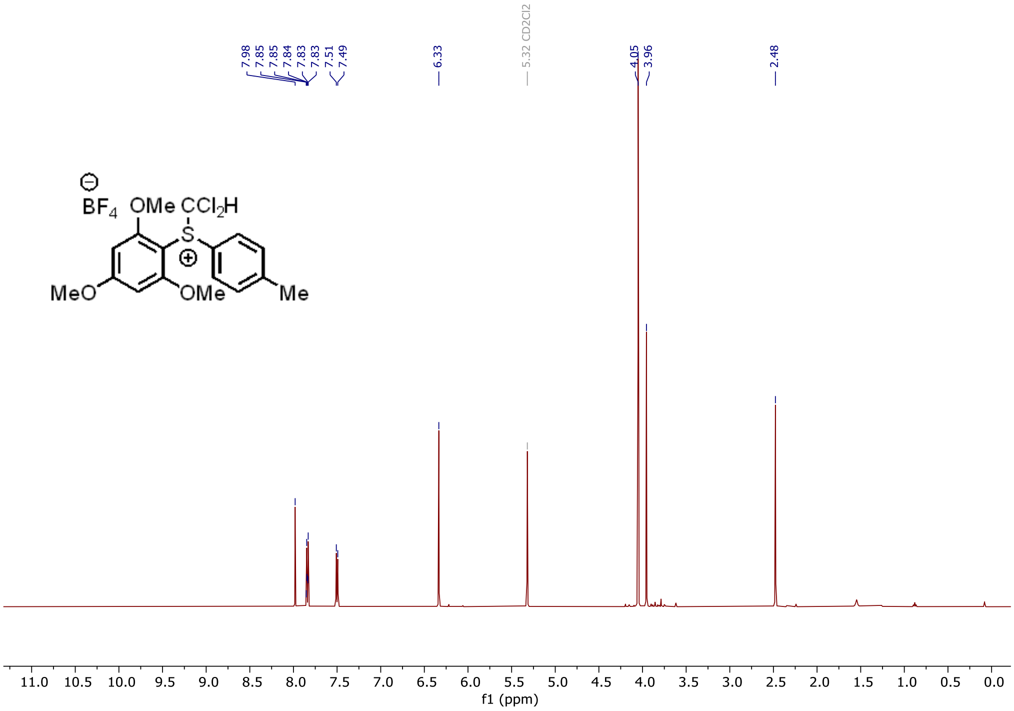

Figure S21: The ^{1}H NMR (500 MHz, CD_2Cl_2) spectra for compound **3c**.

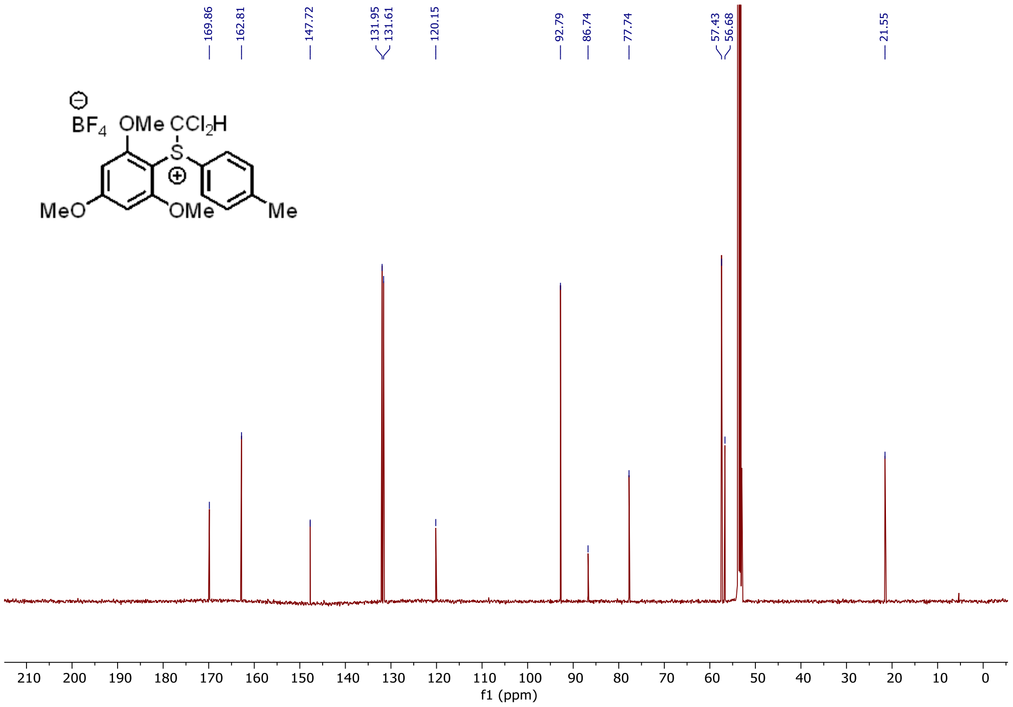

Figure S22: The ^{13}C NMR (101 MHz, CD_2Cl_2) spectra for compound **3c**.

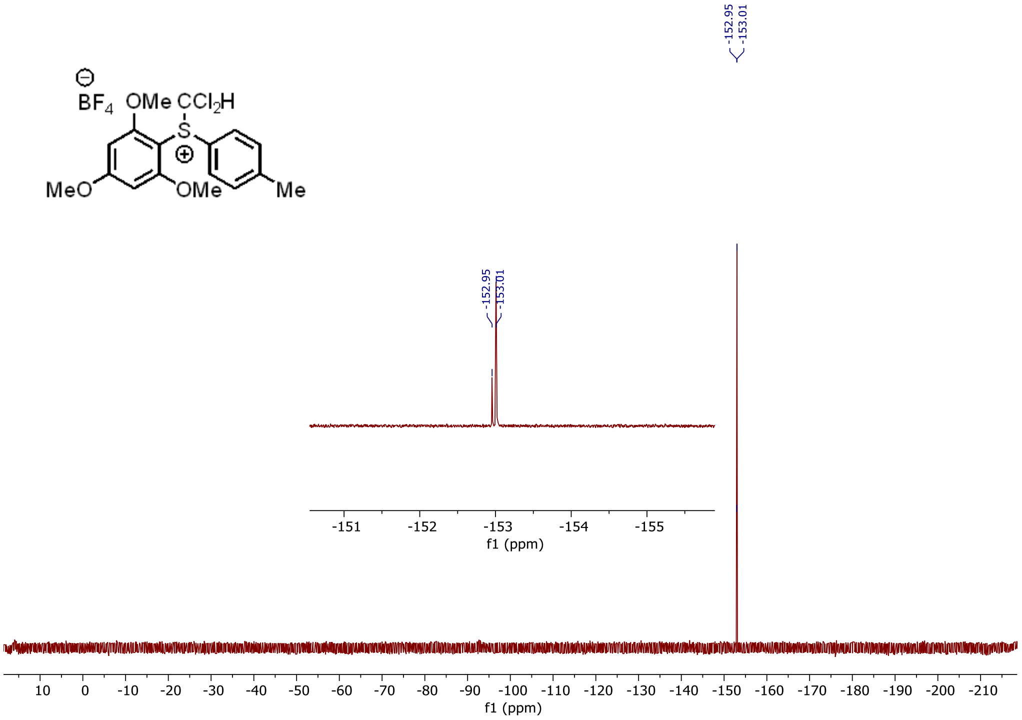

Figure S23: The ^{19}F NMR (376 MHz, CD_2Cl_2) spectra for compound **3c**.

(3d): (dichloromethyl)(4-methoxyphenyl)(2,4,6-trimethoxyphenyl)sulfonium tetrafluoroborate

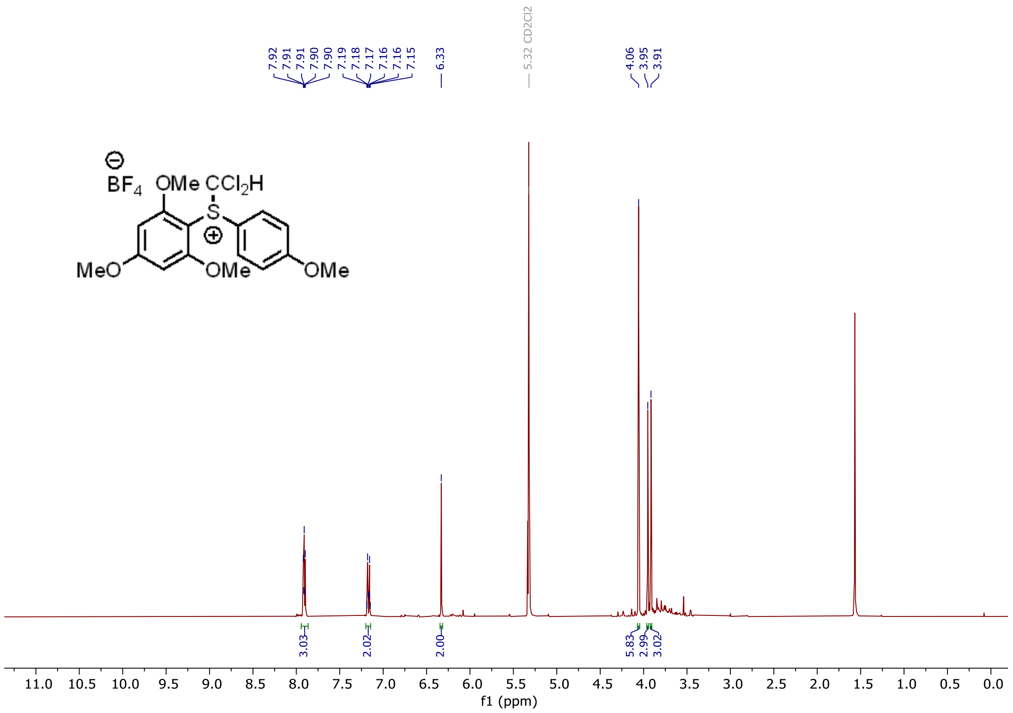

Figure S24: The ^{1}H NMR (400 MHz, CD_2Cl_2) spectra for compound **3d**.

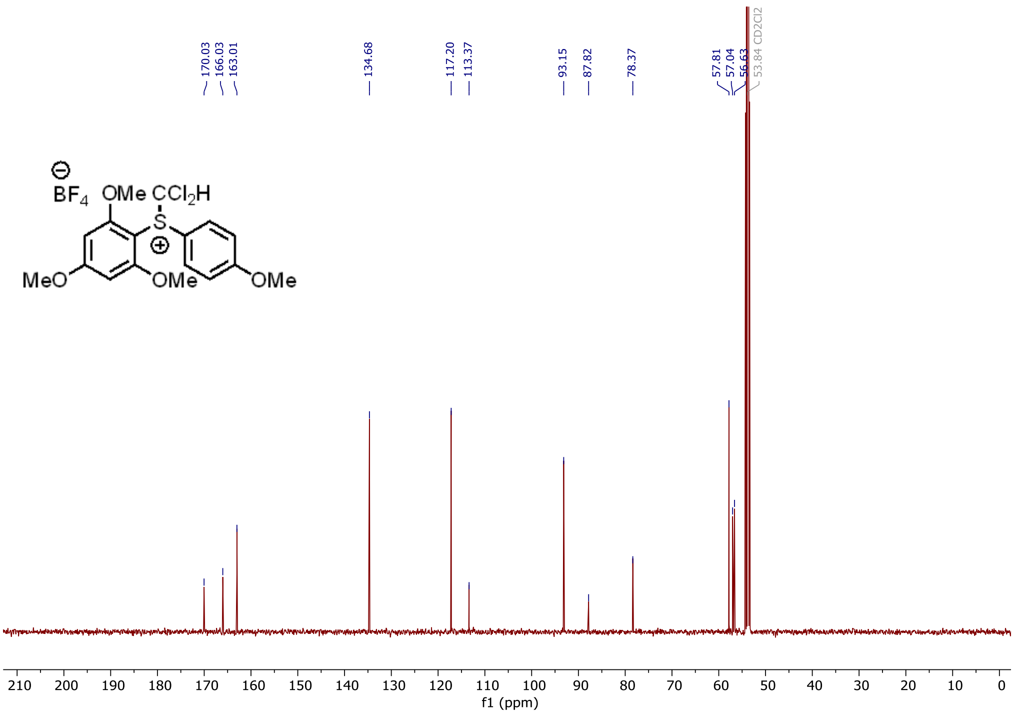

Figure S25: The ^{13}C NMR (101 MHz, CD_2Cl_2) spectra for compound **3d**.

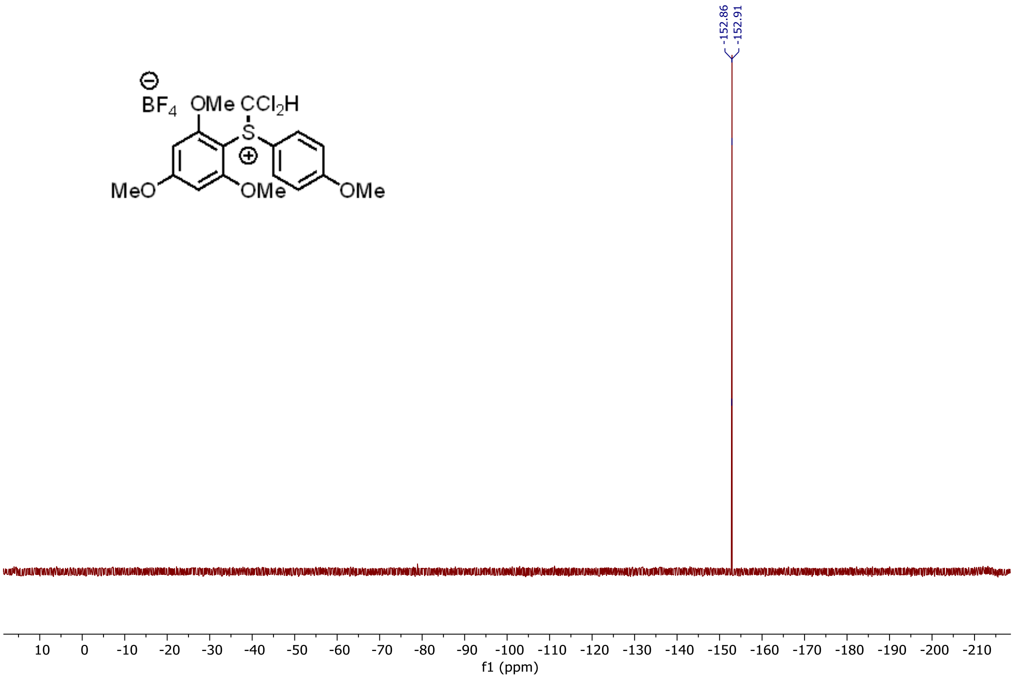

Figure S26: The ^{19}F NMR (376 MHz, CD_2Cl_2) spectra for compound **3d**.

(5a): (2,2-dichloro-1-methylcyclopropyl)benzene

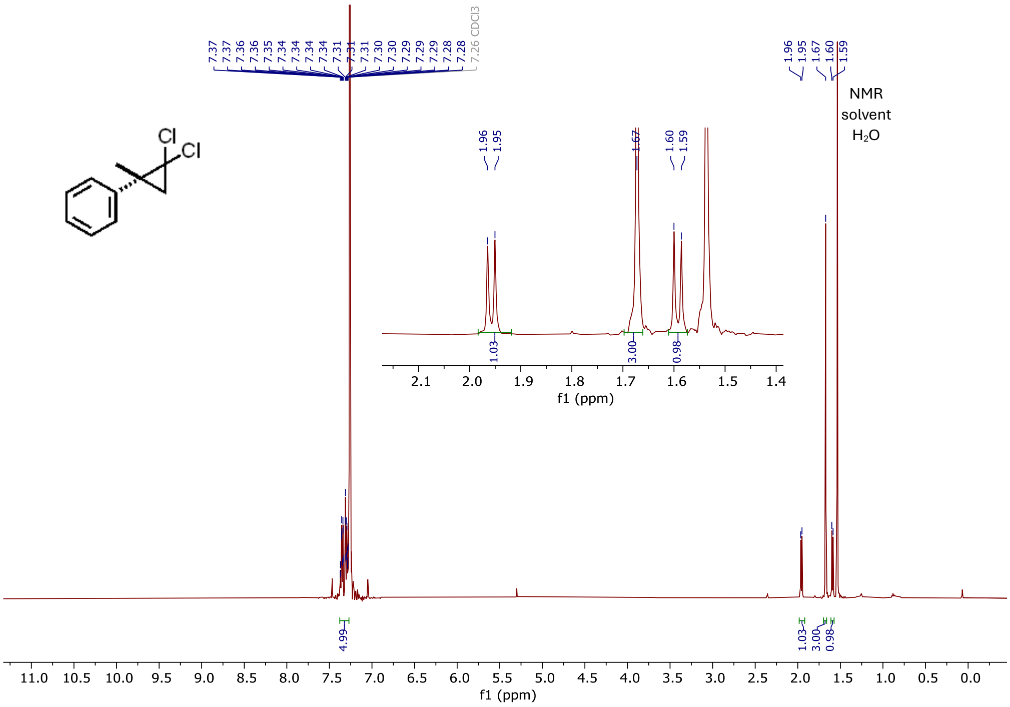

Figure S27: The ^{1}H NMR (500 MHz, $CDCl_3$) spectra for compound **5a**.

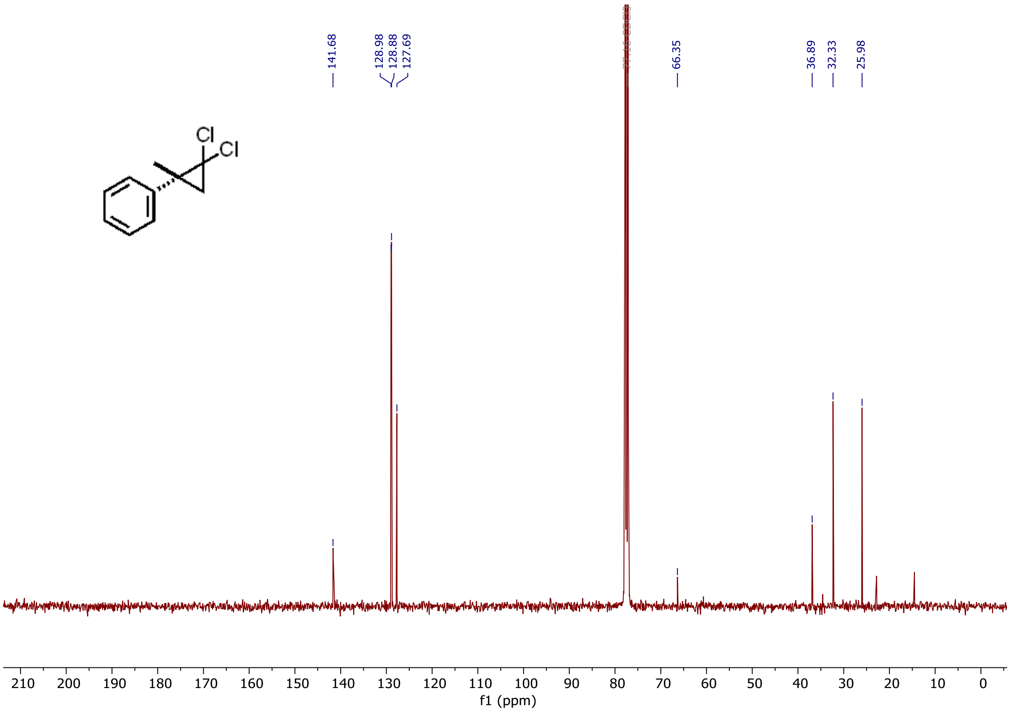

Figure S28: The ^{13}C NMR (101 MHz, $CDCl_3$) spectra for compound **5a**.

(5b): 1-(2,2-dichlorocyclopropyl)-4-methoxybenzene

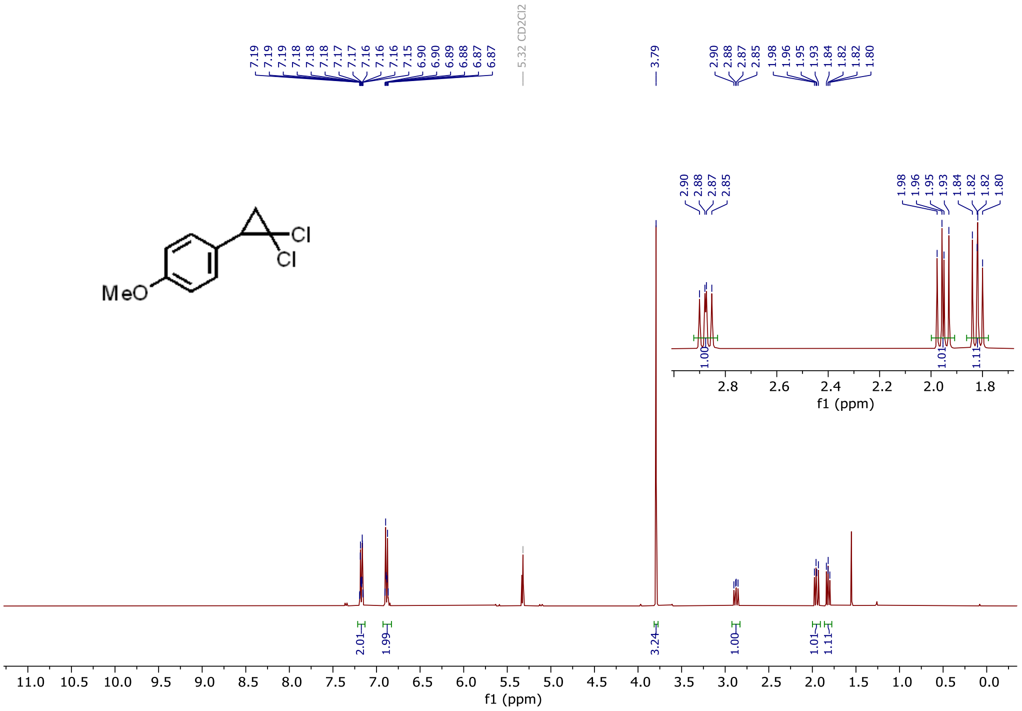

Figure S29: The ^{1}H NMR (400 MHz, CD_2Cl_2) spectra for compound **5b**.

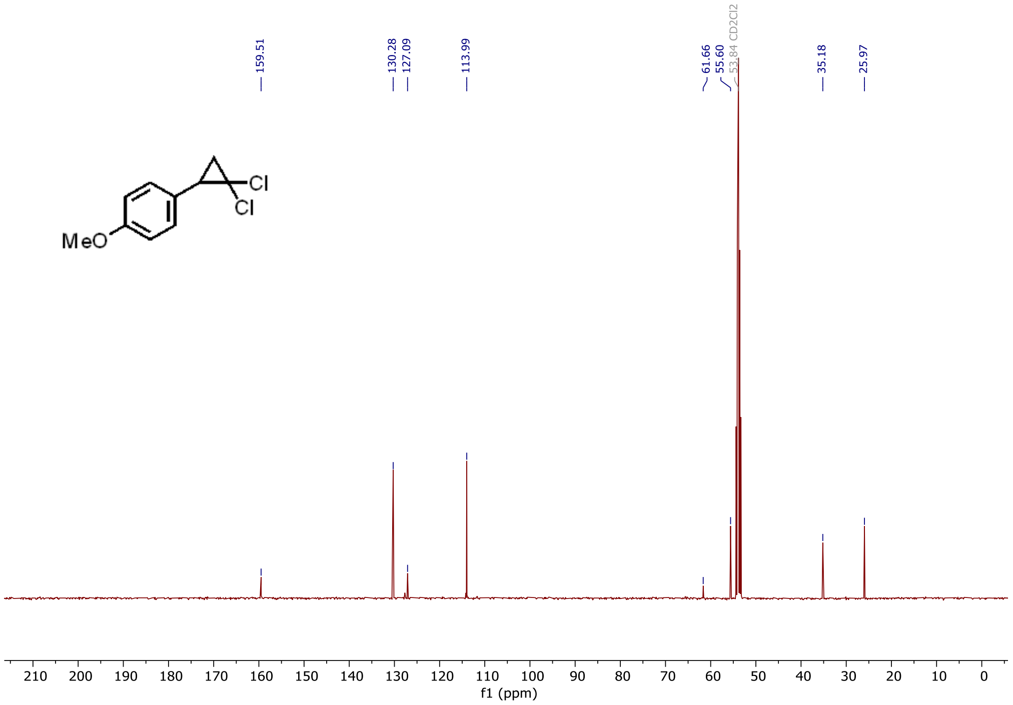

Figure S30: The ^{13}C NMR (101 MHz, CD_2Cl_2) spectra for compound **5b**.

(5c): 1-(2,2-dichlorocyclopropyl)-2-methoxybenzene

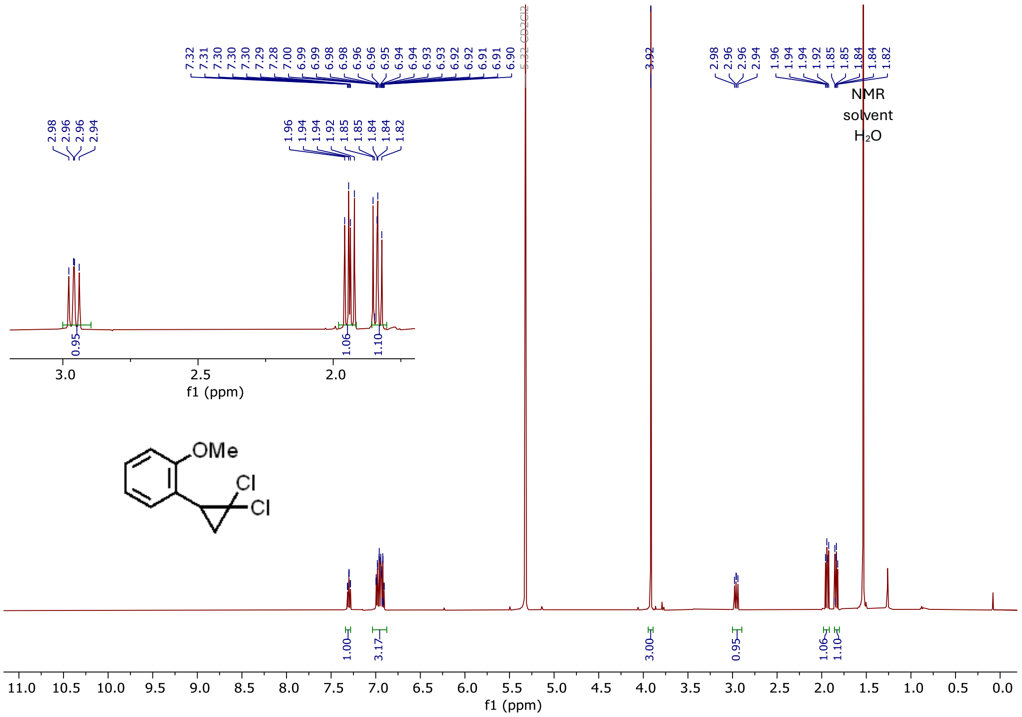

Figure S31: The ^{1}H NMR (500 MHz, CD_2Cl_2) spectra for compound **5c.**

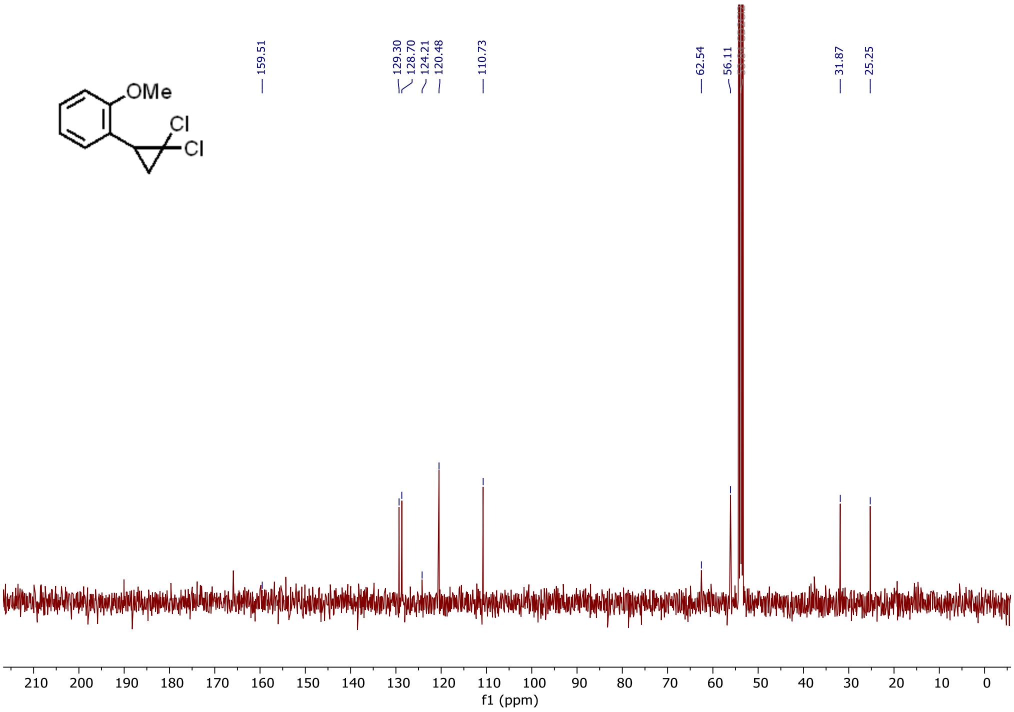

Figure S32: The ^{13}C NMR (126 MHz, CD_2Cl_2) spectra for compound **5c**.

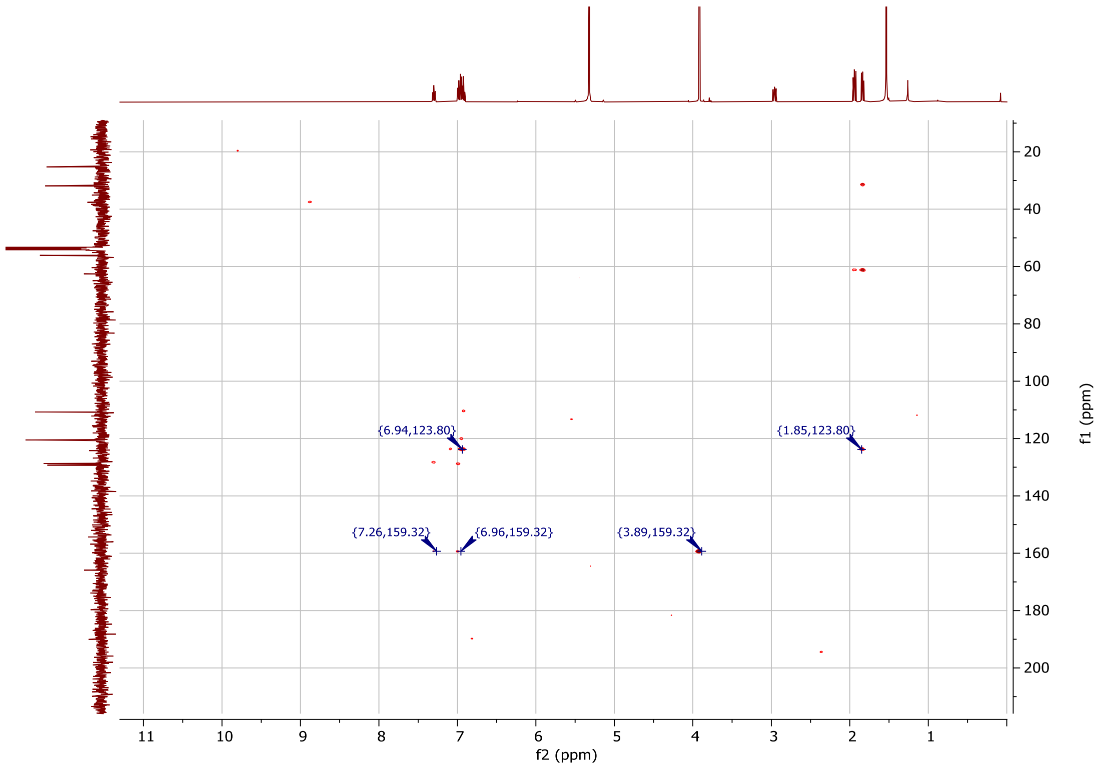

Figure S33: The HMBC for compound **5c** with CD_2Cl_2 as the deuterated solvent.

(5d): 1-(2,2-dichlorocyclopropyl)-3-methoxybenzene

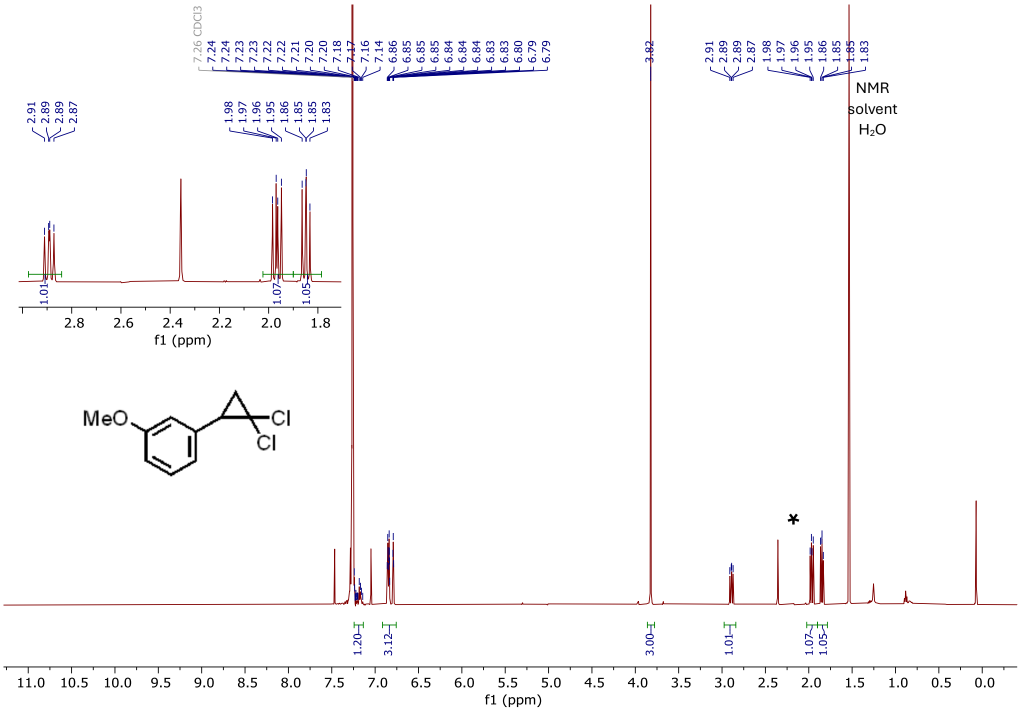

Figure S34: The ^{1}H NMR (500 MHz, $CDCl_3$) spectra for compound **5d**. *Impurity in $CDCl_3$.

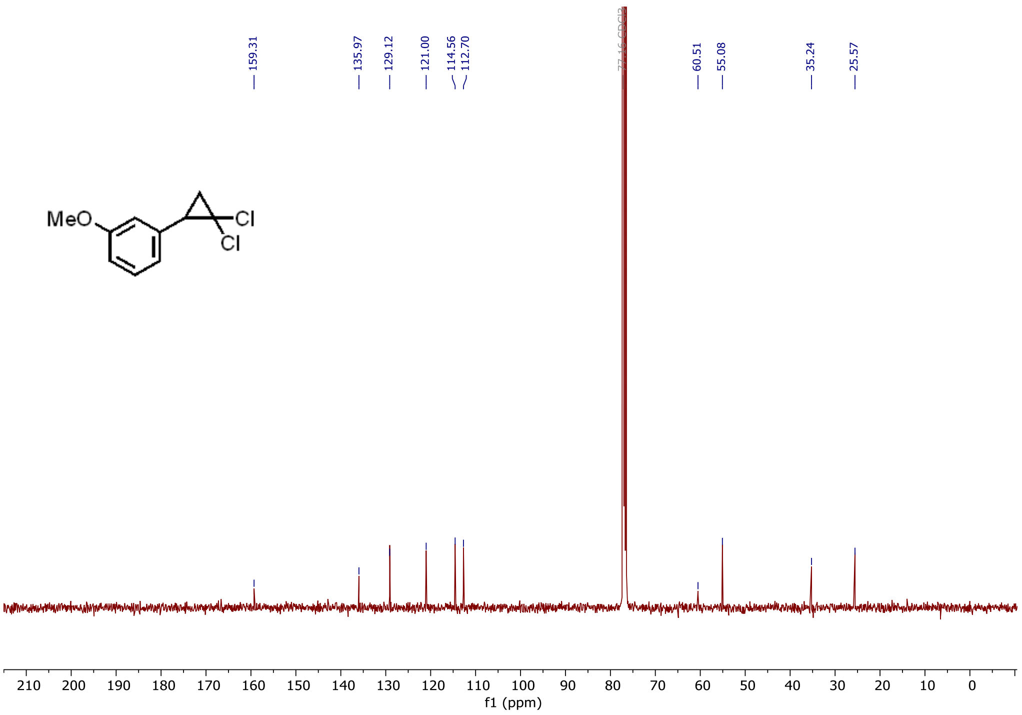

Figure S35: The ^{13}C NMR (101 MHz, $CDCl_3$) spectra for compound **5d**.

(5e): 1-chloro-4-(2,2-dichlorocyclopropyl)benzene

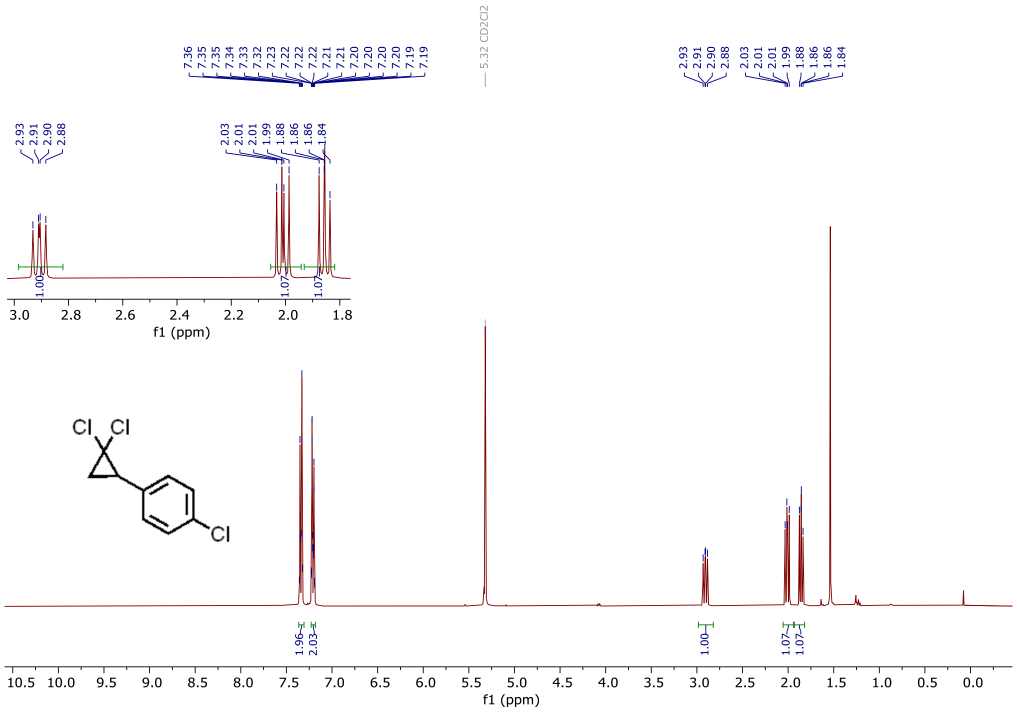

Figure S36: The ^{1}H NMR (400 MHz, CD_2Cl_2) spectra for compound **5e**.

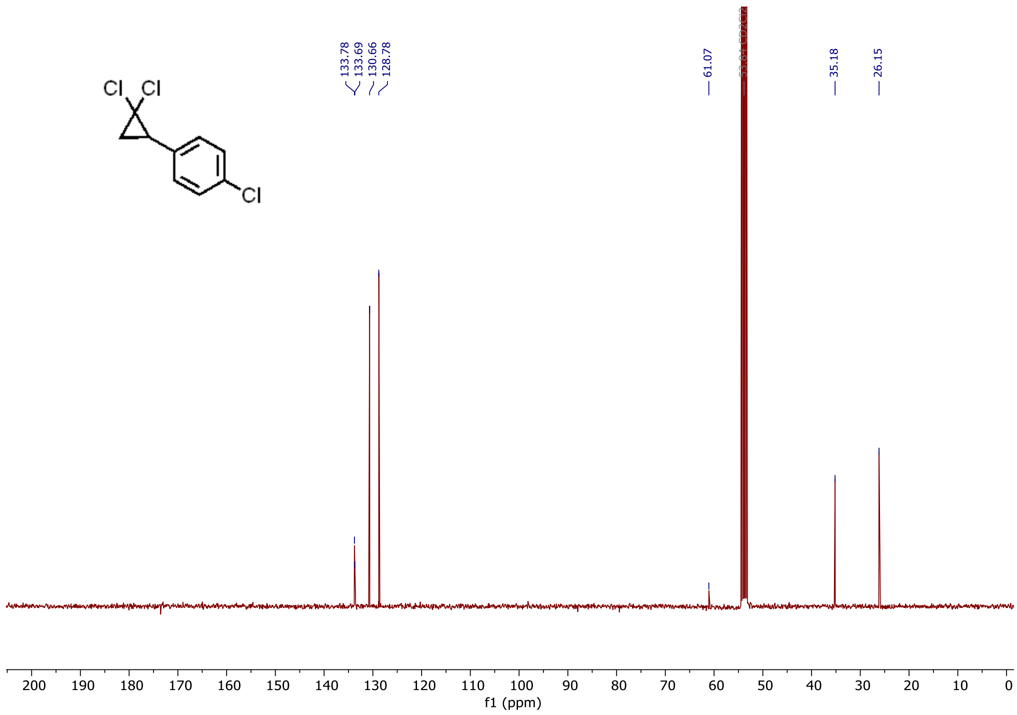

Figure S37: The ^{13}C NMR (101 MHz, CD_2Cl_2) spectra of **5e**.

(5f): (2,2-dichlorocyclopropyl)benzene

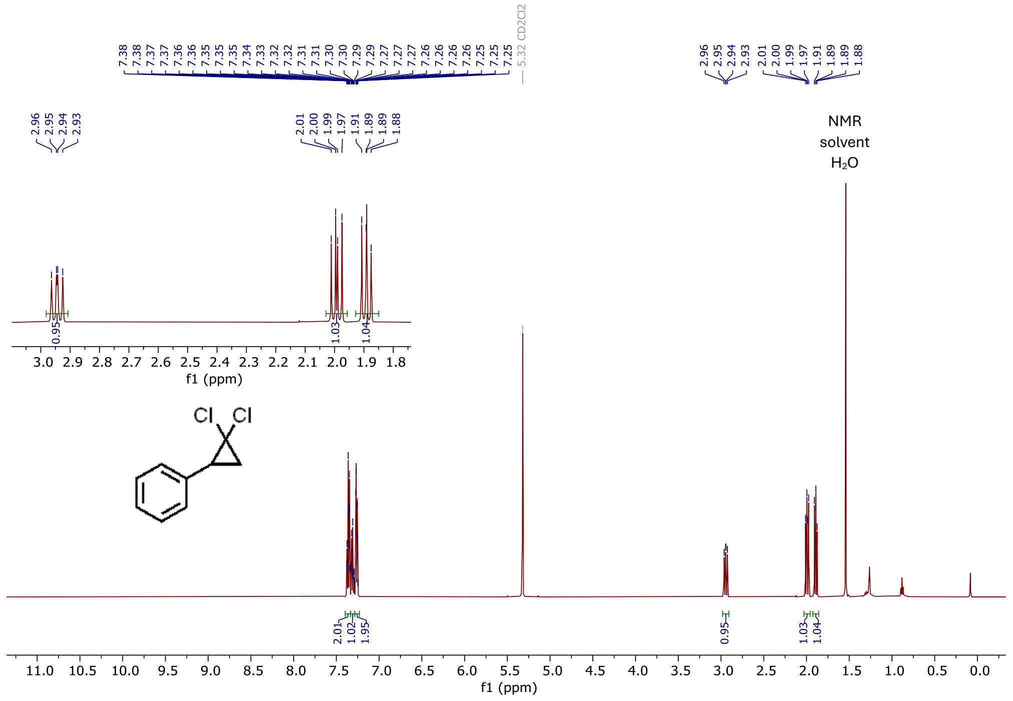

Figure S38: The ^{1}H NMR (500 MHz, CD_2Cl_2) spectra for compound **5f**.

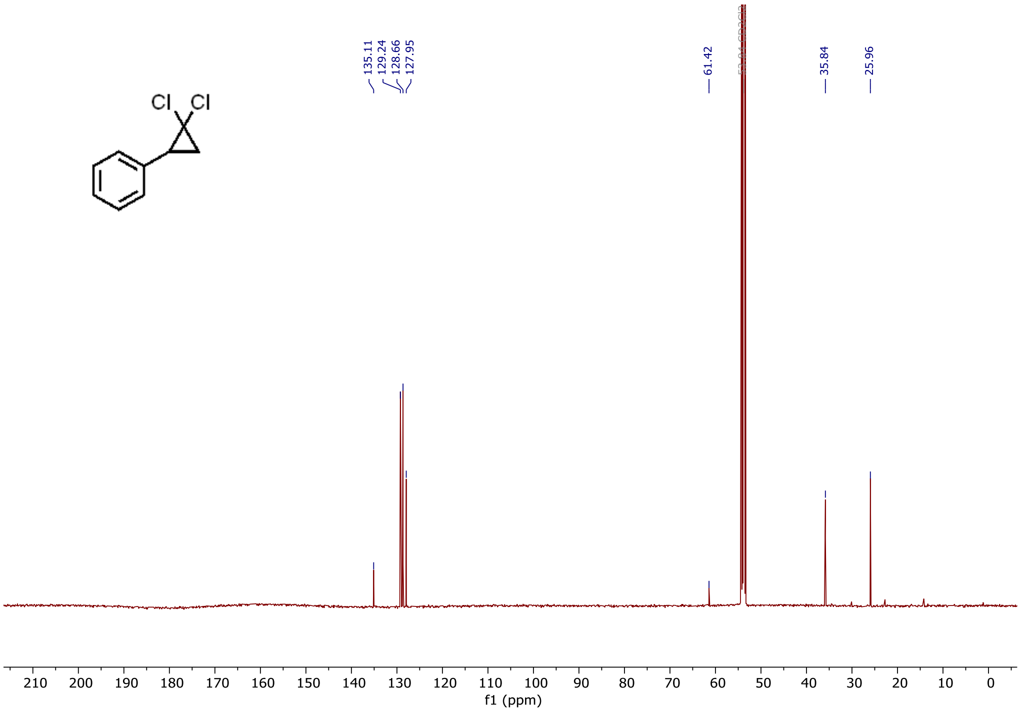

Figure S39: The ^{13}C NMR (126 MHz, CD_2Cl_2) spectra for compound **5f**.

(5g): 2-(2,2-dichlorocyclopropyl)naphthalene

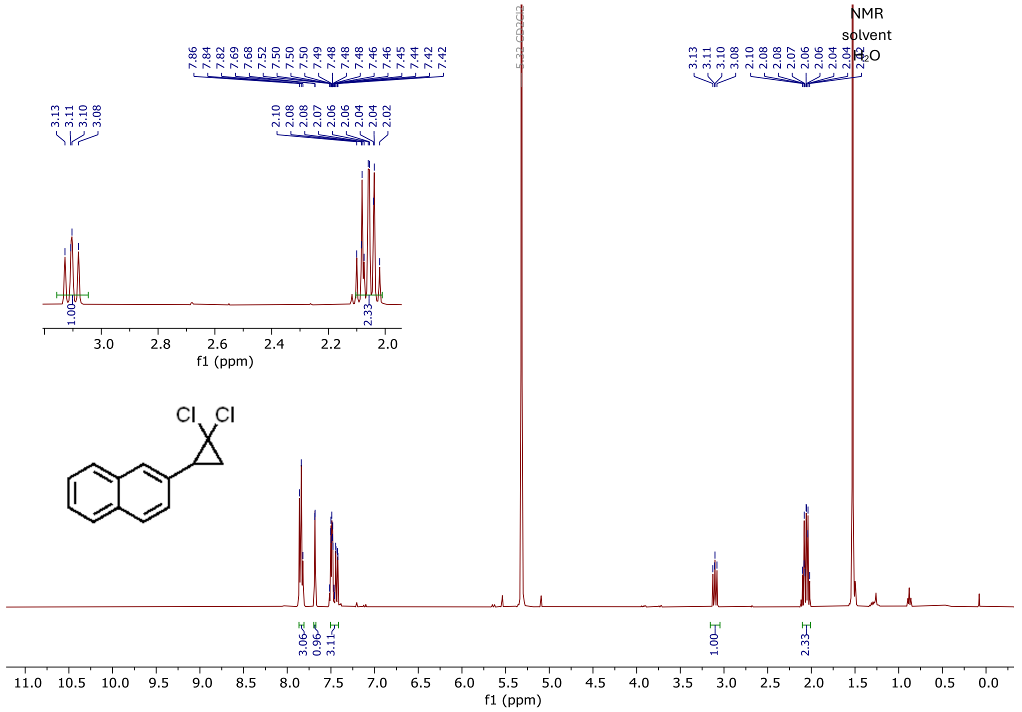

Figure S40: The ^{1}H NMR (400 MHz, CD_2Cl_2) for compound **5g**.

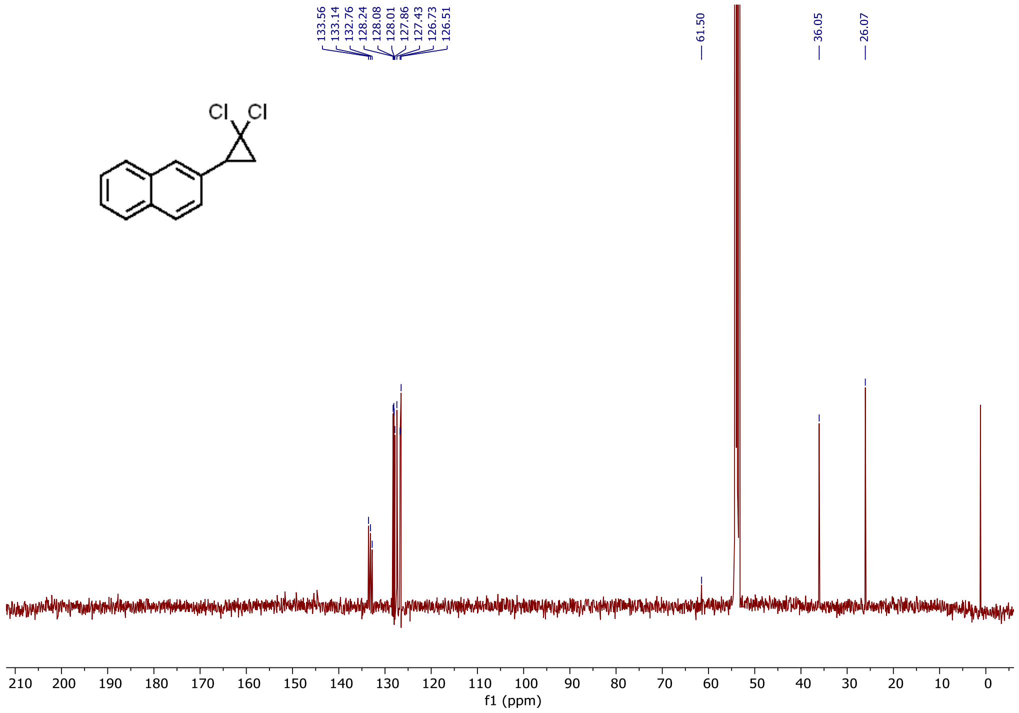

Figure S41: The ^{13}C NMR (126 MHz, CD_2Cl_2) spectra for compound **5g**.

(5h): 4-(2,2-dichlorocyclopropyl)phenyl acetate

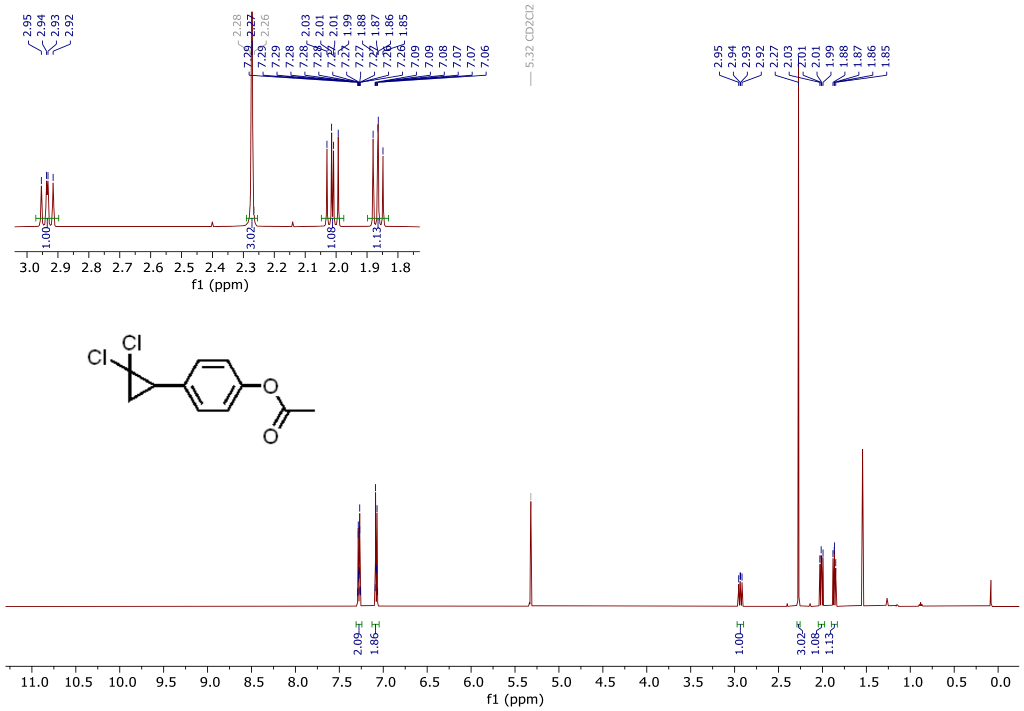

Figure S42. The ^{1}H NMR (500 MHz, CD_2Cl_2) spectra of compound **5h**.

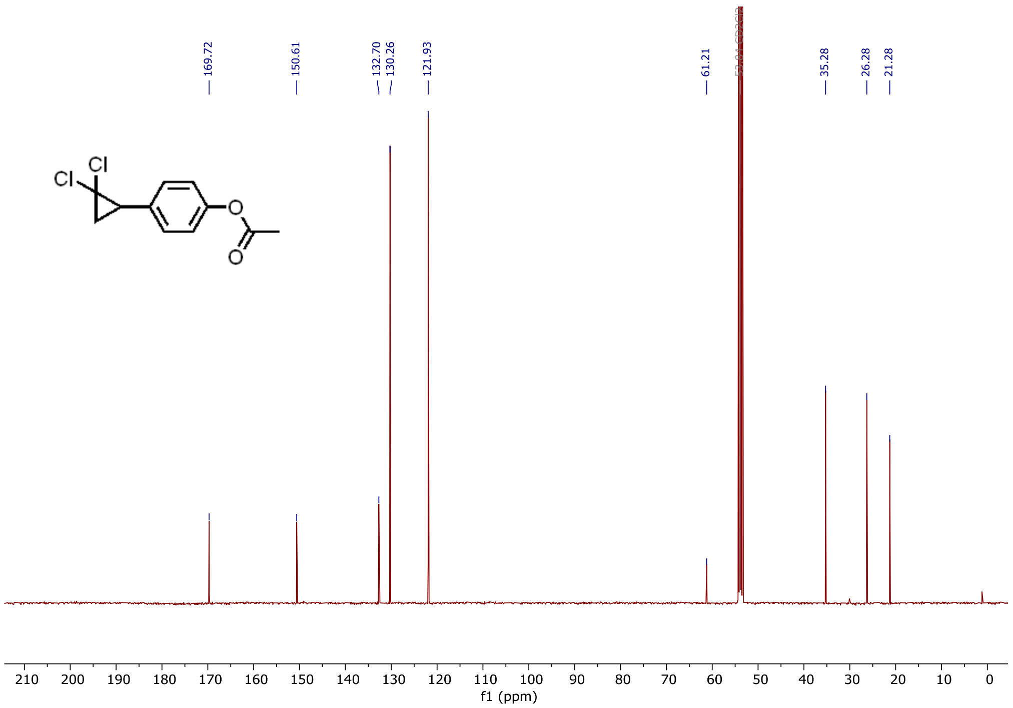

Figure S43. The ^{13}C NMR (126 MHz, CD_2Cl_2) spectra of compound **5h**.

(5i): *trans*-1,1-dichlorospiro[2.3]hexane-5-carbonitrile

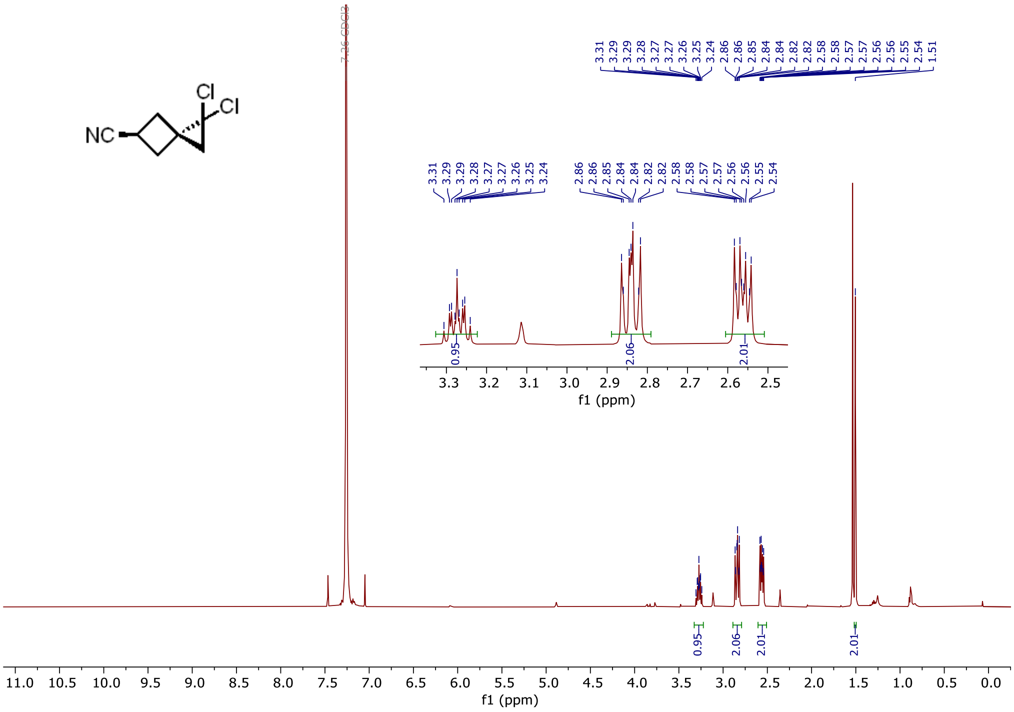

Figure S44: The ^{1}H NMR (500 MHz, $CDCl_3$) spectra for compound **5i**.

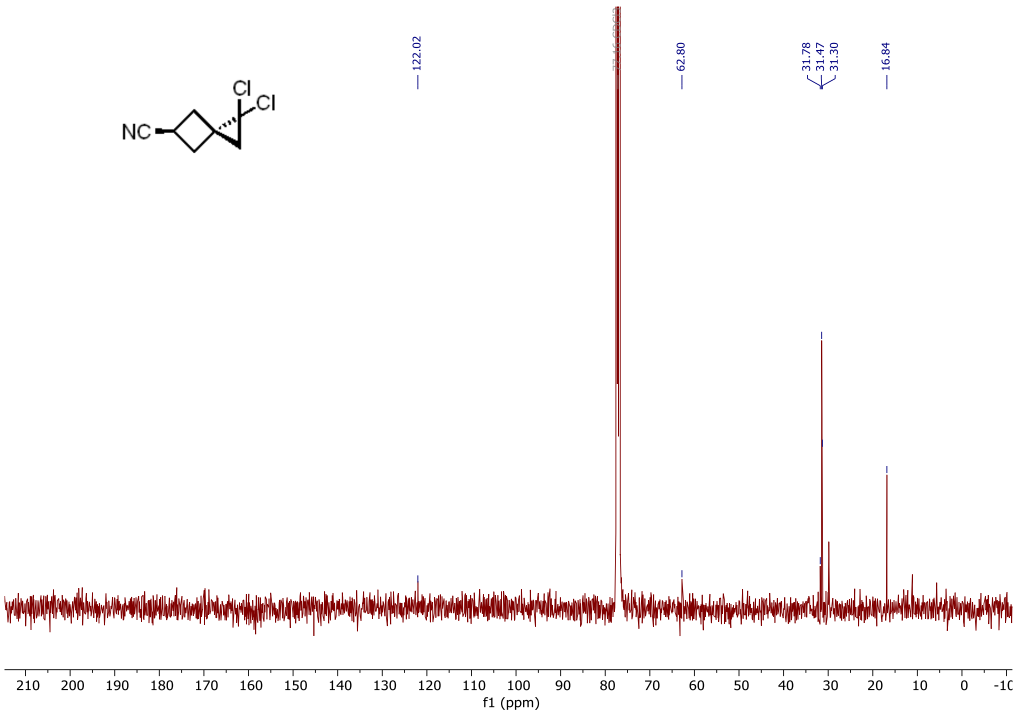

Figure S45: The ^{13}C NMR (101 MHz, $CDCl_3$) spectra for compound **5i**.

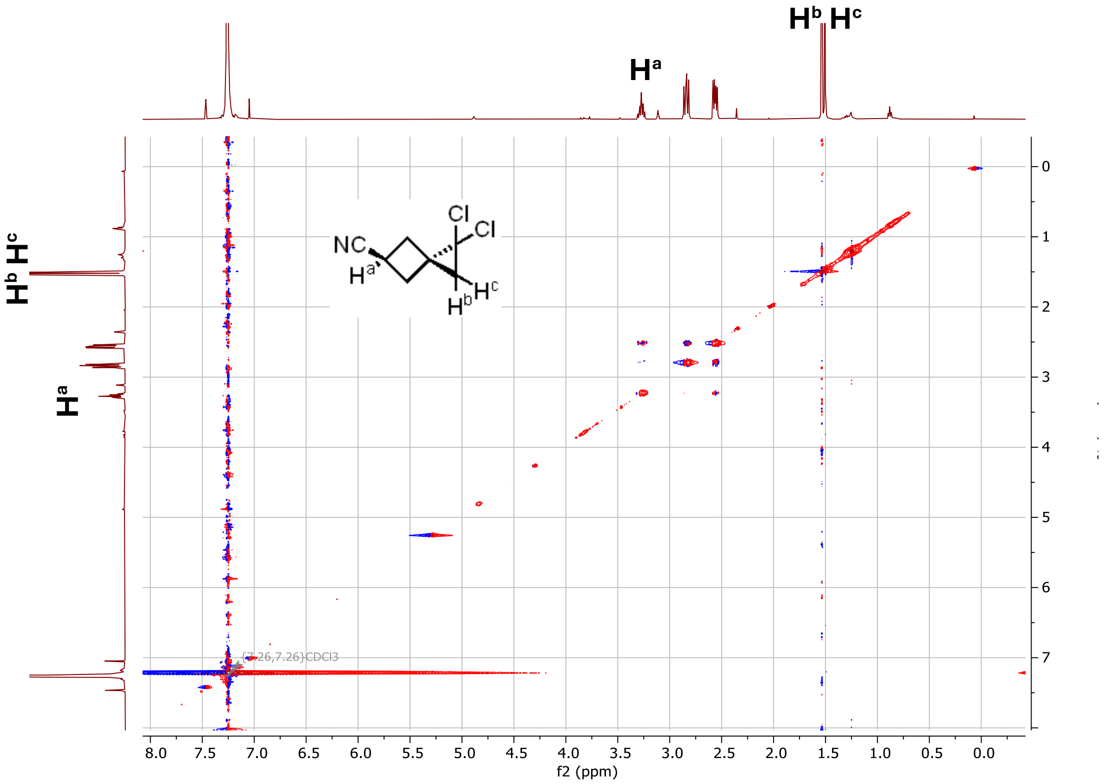

Figure S46: The NOESY NMR spectra for compound **5i** with $CDCl_3$ as the deuterated solvent.

(5j): *tert*-butyl 1,1-dichloro-6-azaspiro[2.5]octane-6-carboxylate

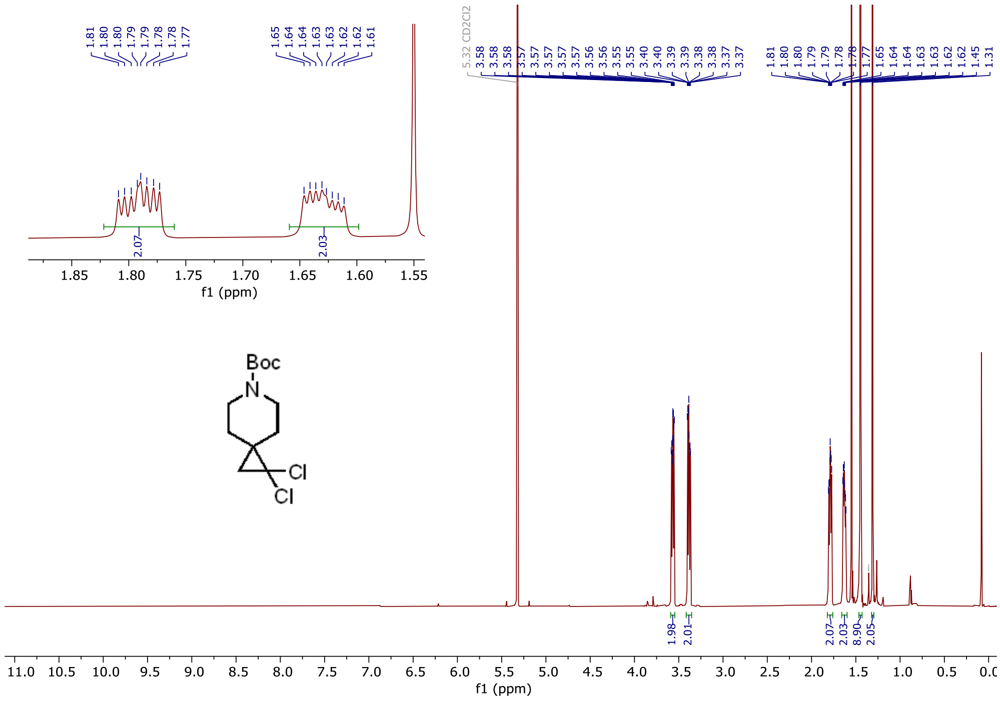

Figure S47: The ^{1}H NMR (700 MHz, CD_2Cl_2) spectra for compound **5j**.

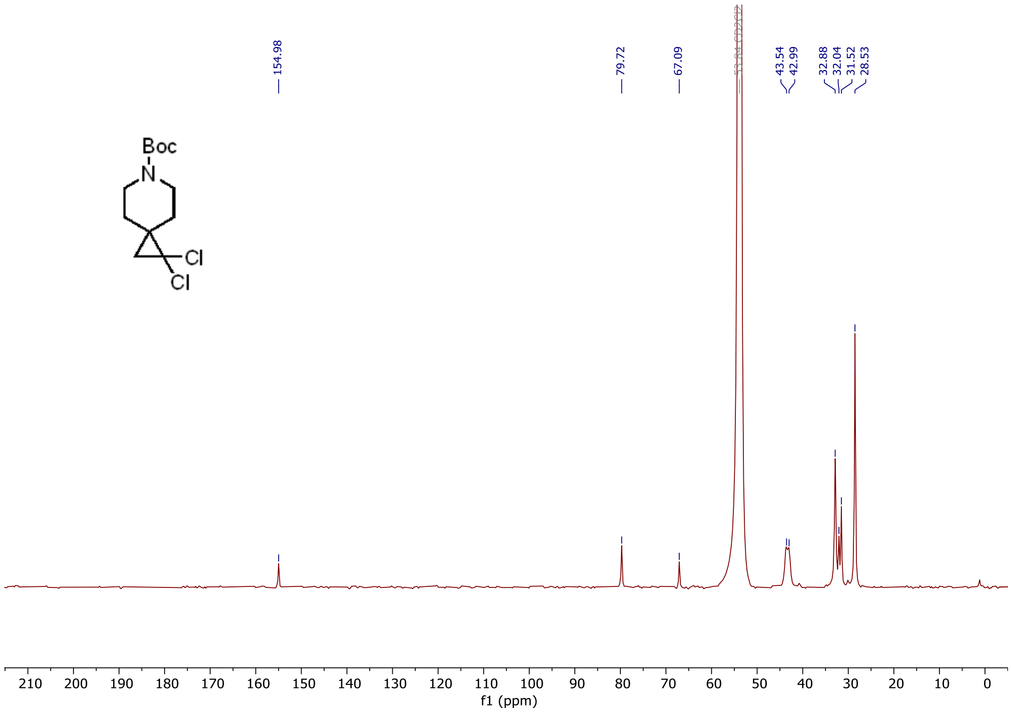

Figure S48: The ^{13}C NMR (176 MHz, CD_2Cl_2) for compound **5j**.

(5k): (*trans*-2,2-dichloro-3-methylcyclopropyl)benzene

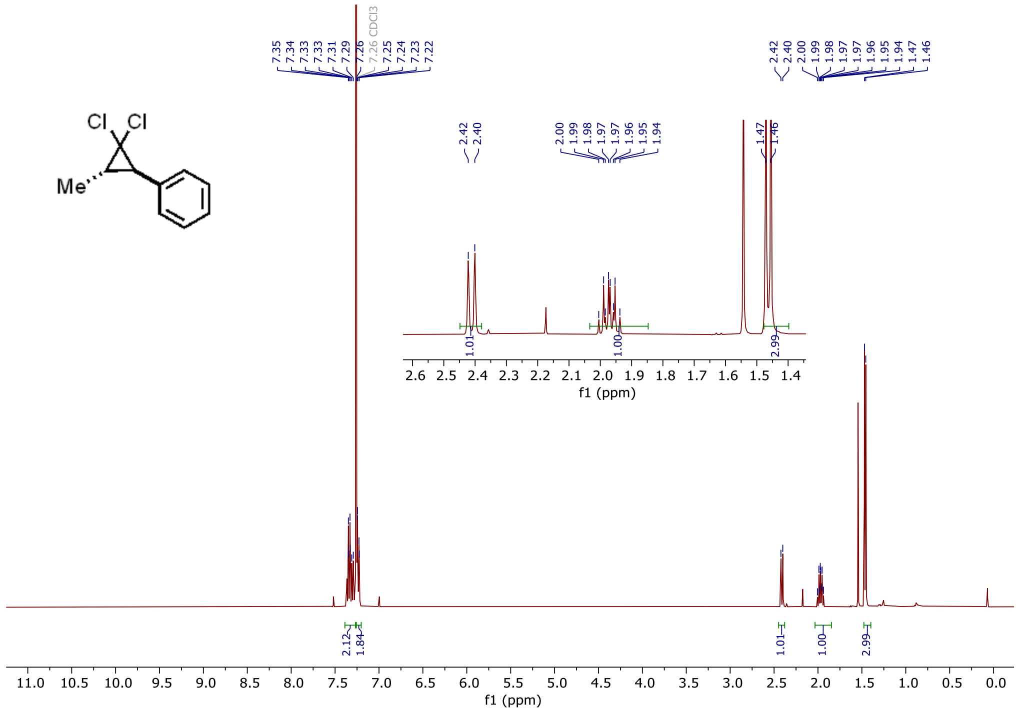

Figure S49: The ^{1}H NMR (400 MHz, $CDCl_3$) for compound **5k**.

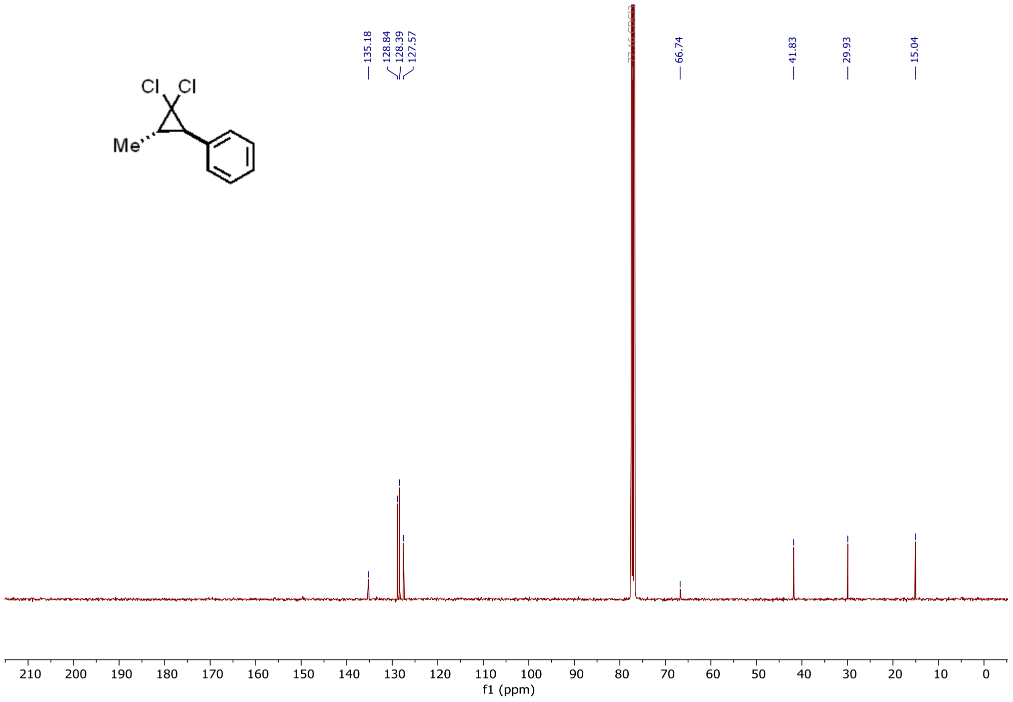

Figure S50: The ^{13}C NMR (101 MHz, $CDCl_3$) spectra for compound **5k**.

(5l): (*cis*-2,2-dichloro-3-methylcyclopropyl)benzene

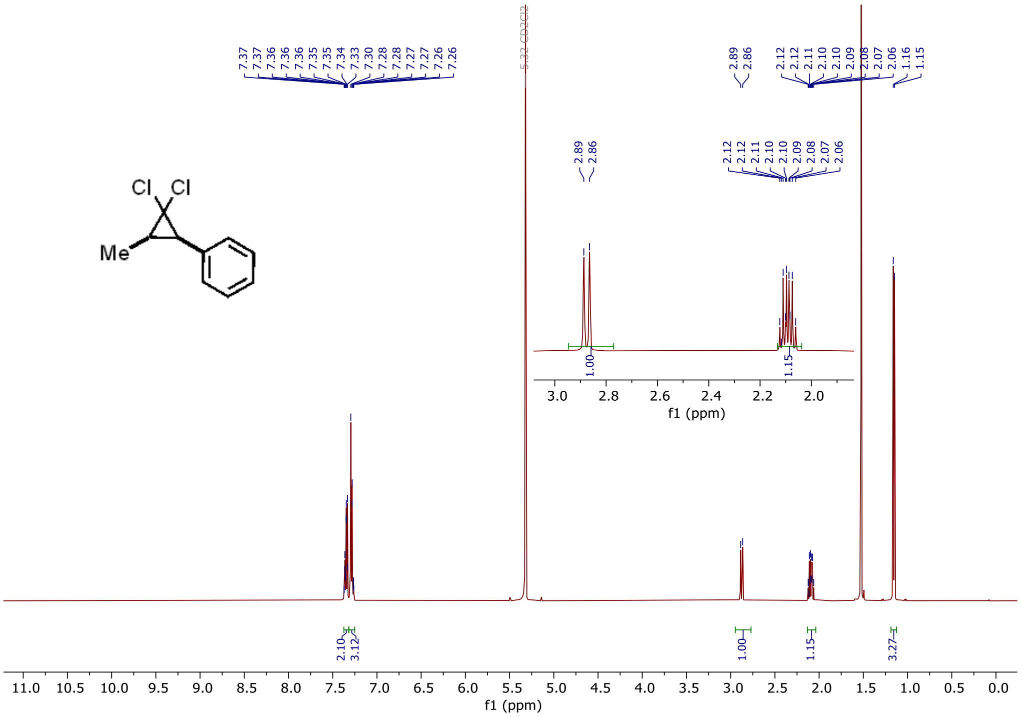

Figure S51: The ^{1}H NMR (400 MHz, CD_2Cl_2) for compound **5l**.

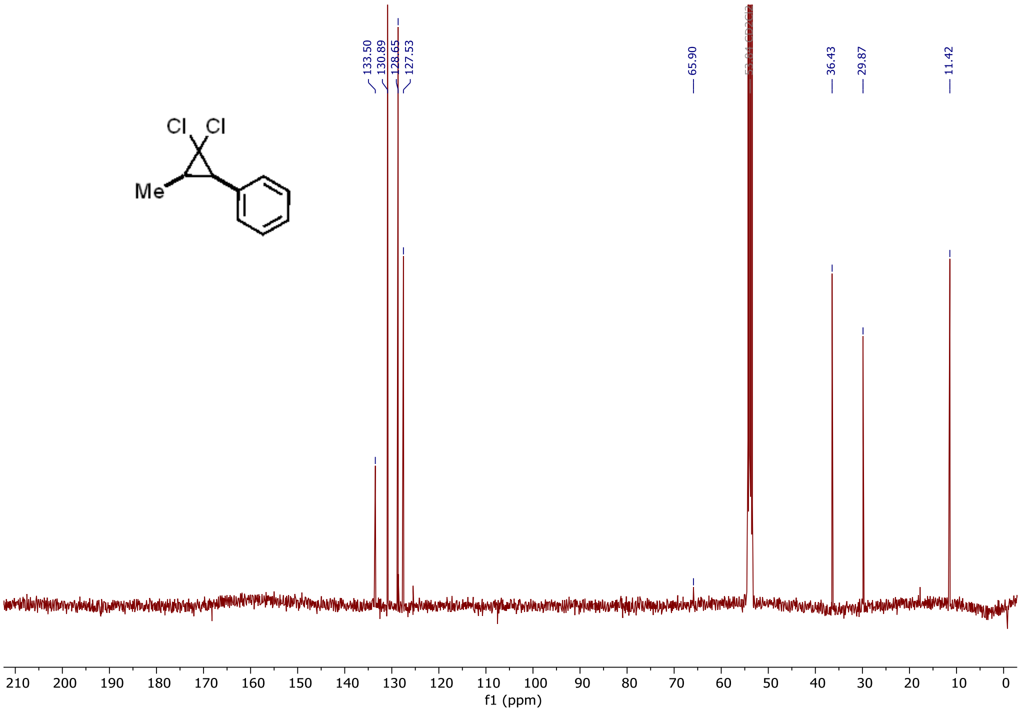

Figure S52: The ^{13}C NMR (126 MHz, CD_2Cl_2) spectra for compound **5l**.

(5m): 7,7-dichloro-1-methylbicyclo[4.1.0]heptane

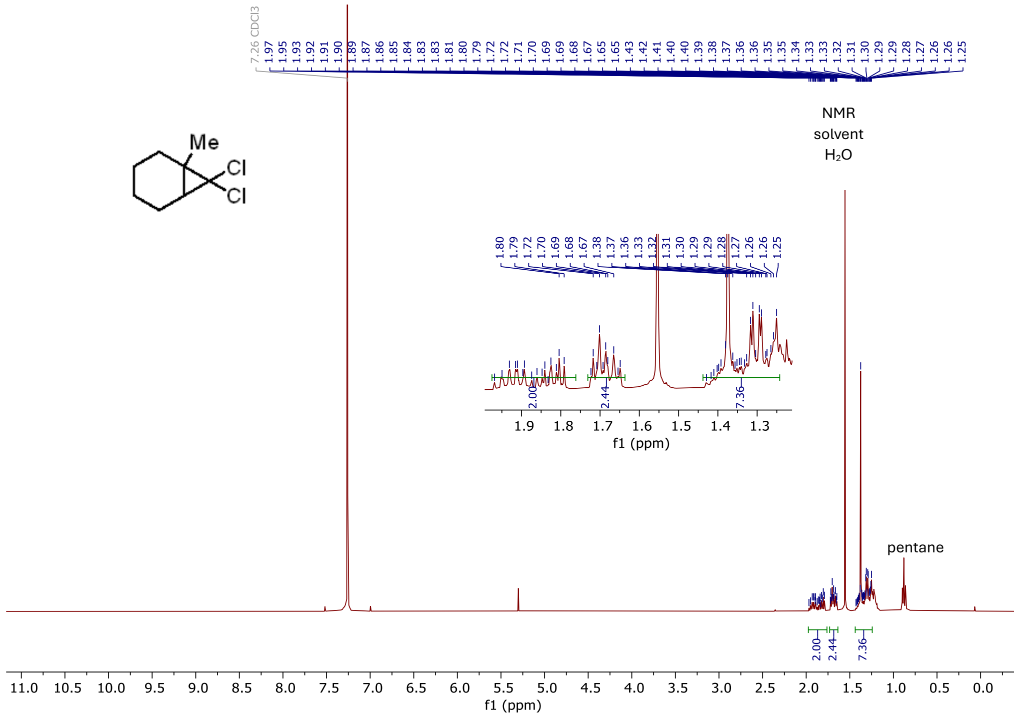

Figure S53: The ^{1}H NMR (400 MHz, $CDCl_3$) spectra for compound **5m**.

(5n): ((7,7-dichlorobicyclo[4.1.0]heptan-1-yl)oxy)trimethylsilane

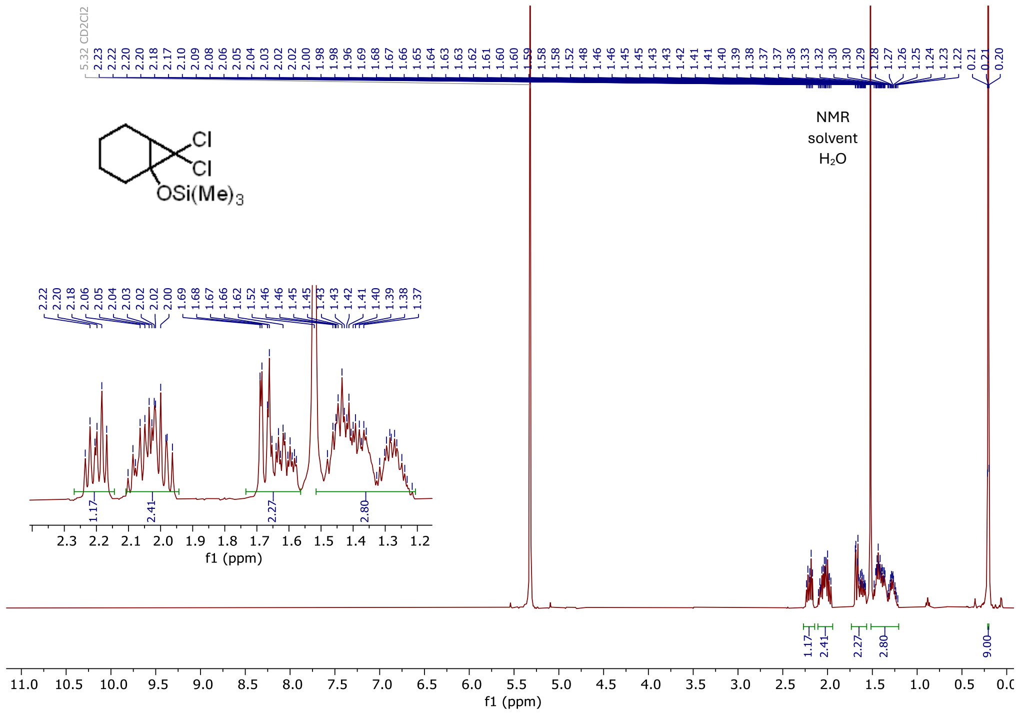

Figure S54: The ^{1}H NMR (400 MHz, CD_2Cl_2) spectra for compound **5n**.

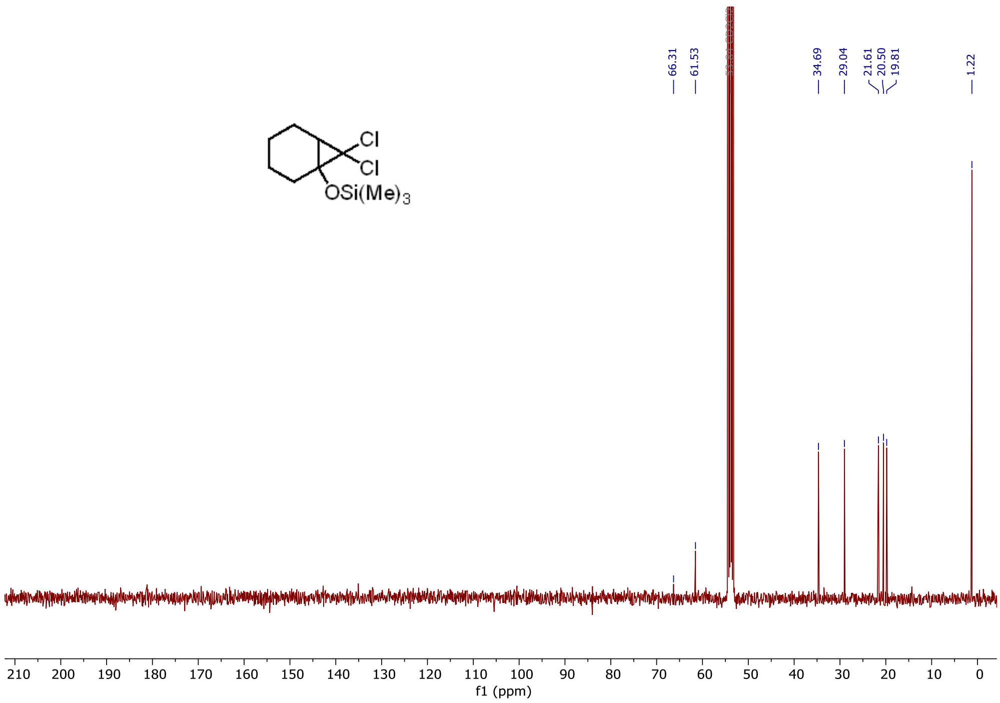

Figure S55: The ^{13}C NMR (101 MHz, CD_2Cl_2) spectra for compound **5n**.

(5o): 2-(2,2-dichloro-3,3-dimethylcyclopropyl)-4,4,5,5-tetramethyl-1,3,2-dioxaborolane

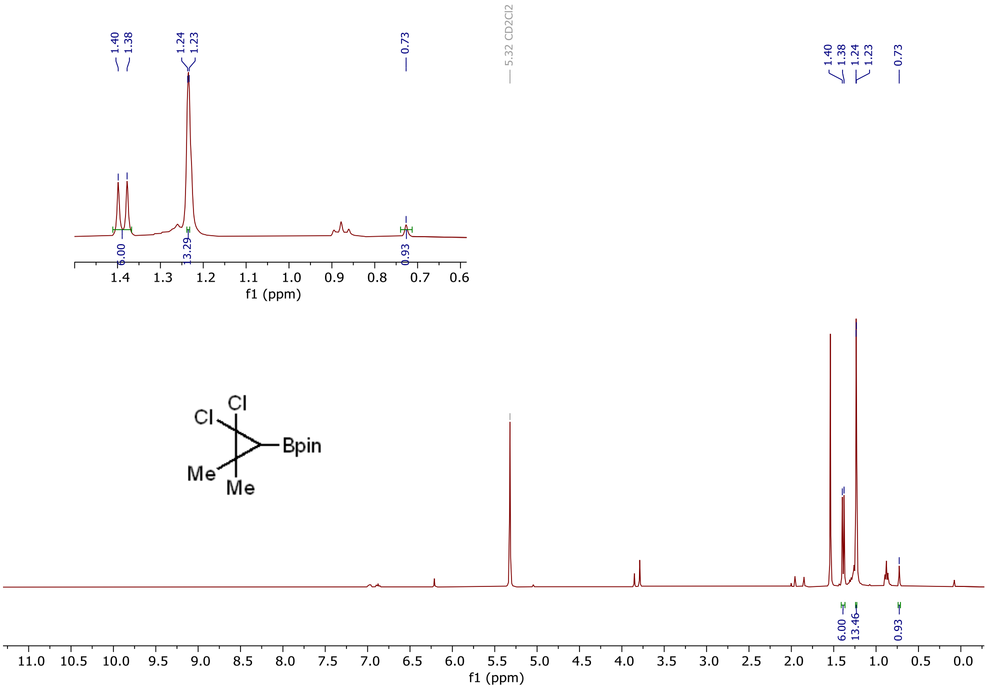

Figure S56: The crude ^{1}H NMR spectra (400 MHz, CD_2Cl_2) for compound **5o**.

(5p): 1,1-dichloro-2,2,3,3-tetramethylcyclopropane

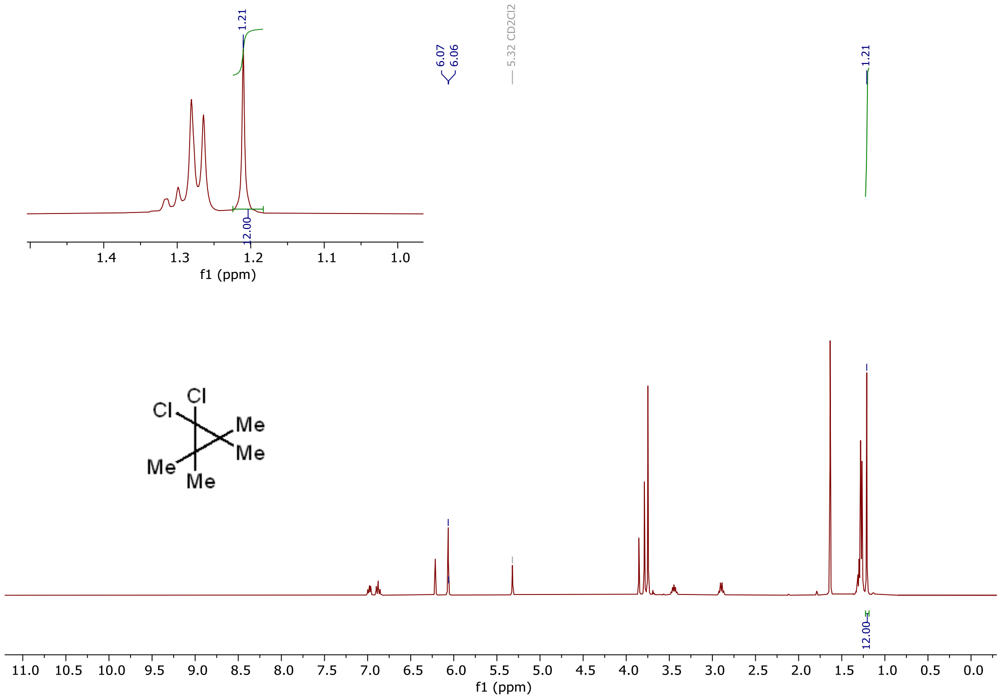

Figure S57: The crude ^1H NMR spectra (400 MHz, CD_2Cl_2) spectra for compound **5p**.
